# Project Earthrise: Proceedings of the Ninth Annual Conference of *in*VIVO Planetary Health

**DOI:** 10.3390/ijerph182010654

**Published:** 2021-10-12

**Authors:** Susan L. Prescott, Ganesa Wegienka, Remco Kort, David H. Nelson, Sabine Gabrysch, Trevor Hancock, Anita Kozyrskyj, Christopher A. Lowry, Nicole Redvers, Blake Poland, Jake Robinson, Jean-Claude Moubarac, Sara Warber, Janet Jansson, Aki Sinkkonen, John Penders, Susan Erdman, Ralph Nanan, Matilda van den Bosch, Kirk Schneider, Nicholas J. Schroeck, Tanja Sobko, Jamie Harvie, George A. Kaplan, Rob Moodie, Laura Lengnick, Isaac Prilleltensky, Yuria Celidwen, Susan H. Berman, Alan C. Logan, Brian Berman

**Affiliations:** 1 NOVA Institute for Health of People, Places and Planet, 1407 Fleet Street, Baltimore, MD 21231, USA; 2 School of Medicine, University of Western Australia, Nedlands, WA 6009, Australia; 3 ORIGINS Project, Telethon Kids Institute at Perth Children’s Hospital, Nedlands, WA 6009, Australia; 4 Department of Family and Community Medicine, Center for Integrative Medicine, University of Maryland School of Medicine, Baltimore, MD 21201, USA; swarber@med.umich.edu (S.W.); gkaplan@umich.edu (G.A.K.); sberman@tiih.org (S.H.B.); alanxlogan@gmail.com (A.C.L.); bberman@tiih.org (B.B.); 5 Department of Public Health Sciences, Henry Ford Hospital, Detroit, MI 48202, USA; gwegien1@hfhs.org; 6 Department of Molecular Cell Biology, Vrije Universiteit Amsterdam, De Boelelaan 1108, 1081 HZ Amsterdam, The Netherlands; r.kort@vu.nl; 7 ARTIS_Micropia, Plantage Kerklaan 38-40, 1018 CZ Amsterdam, The Netherlands; 8 Independent Researcher, Woodstock, ON N4S 6Y9, Canada; davidhplanet@gmail.com; 9 Heidelberg Institute of Global Health, Heidelberg University, 69120 Heidelberg, Germany; sabine.gabrysch@uni-heidelberg.de; 10 Potsdam Institute for Climate Impact Research (PIK), Member of the Leibniz Association, 14412 Potsdam, Germany; 11 Institute of Public Health, Charité—Universitätsmedizin Berlin, 10117 Berlin, Germany; 12 School of Public Health and Social Policy, University of Victoria, Victoria, BC V8W 2Y2, Canada; thancock@uvic.ca; 13 Department of Pediatrics, Faculty of Medicine & Dentistry, Alberta, Edmonton Clinic Health Academy, Edmonton, AB T6G 1C9, Canada; kozyrsky@ualberta.ca; 14 Department of Integrative Physiology, University of Colorado Boulder, Boulder, CO 80309, USA; christopher.lowry@colorado.edu; 15 School of Medicine and Health Sciences, University of North Dakota, Grand Forks, ND 58202, USA; nicole.redvers@UND.edu; 16 Dalla Lana School of Public Health, University of Toronto, Toronto, ON M5T 3M7, Canada; blake.poland@utoronto.ca; 17 Department of Landscape, The University of Sheffield, Sheffield S10 2TN, UK; jmrobinson3@sheffield.ac.uk; 18 Department of Nutrition, Faculty of Medicine, University of Montréal, Montreal, QC H3T 1J4, Canada; jc.moubarac@umontreal.ca; 19 Department of Family Medicine, University of Michigan, Ann Arbor, MI 48109, USA; 20 Biological Sciences Division, Pacific Northwest National Laboratory, Richland, Washington, DC 99352, USA; janet.jansson@pnnl.gov; 21 Natural Resources Institute Finland, Itäinen Pitkäkatu 4, 20520 Turku, Finland; aki.sinkkonen@luke.fi; 22 Department of Medical Microbiology, School for Nutrition & Translational Research in Metabolism (NUTRIM) and Care and Public Health Research Institute (CAPHRI), Maastricht UMC+ [University Medical Centre], 6202 AZ Maastricht, The Netherlands; j.penders@maastrichtuniversity.nl; 23 Division of Comparative Medicine, Massachusetts Institute of Technology, Cambridge, MA 02139, USA; serdman@mit.edu; 24 Sydney Medical School–Nepean, University of Sydney, Camperdown, NSW 2006, Australia; ralph.nanan@sydney.edu.au; 25 School of Population and Public Health, The University of British Columbia, Vancouver, BC V6T 1Z4, Canada; matilda.vandenbosch@ubc.ca; 26 Existential-Humanistic Institute, San Francisco, CA 94901, USA; Kschneider56@gmail.com; 27 Department of Humanistic and Clinical Psychology, Saybrook University, Pasadena, CA 91103, USA; 28 Teachers College, Columbia University, New York, NY 10027, USA; 29 School of Law, University of Detroit Mercy, 651 East Jefferson Ave., Detroit, MI 48226, USA; schroenj@udmercy.edu; 30 School of Biological Sciences, University of Hong Kong, Hong Kong, China; tsobko@hku.hk; 31 Institute for a Sustainable Future, Duluth, MN 55802, USA; harvie@isfusa.org; 32 School of Public Health, University of Michigan, Ann Arbor, MI 48109, USA; 33 School of Population and Global Health (MSPGH), University of Melbourne, Parkville, VIC 3010, Australia; r.moodie@unimelb.edu.au; 34 Cultivating Resilience, LLC, Asheville, NC 28805, USA; laura@cultivatingresilience.com; 35 School of Education and Human Development, University of Miami, Coral Gables, FL 33124, USA; isaacp@miami.edu; 36 Independent Researcher, New York, NY 10017, USA; celidwen@hotmail.com

**Keywords:** planetary health, grand challenges, Anthropocene, Symbiocene, collaboration, interdependence, social and economic justice, interdisciplinary research, resilience thinking, the great transition, biodiversity losses, climate change, environmental degradation, public health, ecology, anthropology, political/social/environmental sciences, philosophy, geography, spirituality, human culture, history and tradition, architecture and design, arts, ethics, wisdom, and Indigenous governance

## Abstract

The “Earthrise” photograph, taken on the 1968 Apollo 8 mission, became one of the most significant images of the 20th Century. It triggered a profound shift in environmental awareness and the potential for human unity—inspiring the first Earth Day in 1970. Taking inspiration from these events 50 years later, we initiated *Project Earthrise* at our 2020 annual conference of *in*VIVO Planetary Health. This builds on the emergent concept of planetary health, which provides a shared narrative to integrate rich and diverse approaches from all aspects of society towards shared solutions to global challenges. The acute catastrophe of the COVID-19 pandemic has drawn greater attention to many other interconnected global health, environmental, social, spiritual, and economic problems that have been underappreciated or neglected for decades. This is accelerating opportunities for greater collaborative action, as many groups now focus on the necessity of a “Great Transition”. While ambitious integrative efforts have never been more important, it is imperative to apply these with mutualistic value systems as a compass, as we seek to make wiser choices. *Project Earthrise* is our contribution to this important process. This underscores the imperative for creative ecological solutions to challenges in all systems, on all scales with advancing global urbanization in the digital age—for personal, environmental, economic and societal health alike. At the same time, our agenda seeks to equally consider our social and spiritual ecology as it does natural ecology. Revisiting the inspiration of “Earthrise”, we welcome diverse perspectives from across all dimensions of the arts and the sciences, to explore novel solutions and new normative values. Building on academic rigor, we seek to place greater value on imagination, kindness and mutualism as we address our greatest challenges, for the health of people, places and planet.

## 1. Introduction—Setting the Scene for *Project Earthrise*

There could not be a more important time to imagine a better world, and fundamentally question the way we choose to live on our planet. How we see ourselves. How we treat others. How we care for our place, our communities, and our ecosystems. Even the *way* that we approach our problems. In this sense, our greatest challenges may also create our greatest opportunities to change how we do things, and how we work together on a global scale—in ways we never have before [1].

The grand challenges of the Anthropocene are of our making, and ultimately stem from human attitudes and relationships to each other and to our environment—with war and destruction, poverty and inequality, disease and despair, biodiversity loss, climate change, famine, pandemics, increasing polarization, and social unrest, as a consequence. The future of human and environmental health is enmeshed with politics, economics, public policies (or lack, thereof), and social values. Concepts of planetary health recognize these interwoven complexities but must also confront the underlying worldview that created and continue to perpetuate these interconnected problems.

Cross-sectoral, transdisciplinary approaches are therefore fundamental to address these entwined issues [2], and to place a higher value on shared guiding principles that encourage altruism and deeper awareness of interdependence [3]. It is increasingly critical to remove artificial lines of distinction between disciplines, and create more opportunities for specialists in all professions, to share and integrate information. Moreover, if we are to realistically find, evaluate and implement meaningful solutions, vigorous melding of transdisciplinary thought must also be applied with deeper ethical considerations, as an urgent necessity—addressing the underlying value systems that created our greatest challenges in the first place. 

It was with these many notions that we launched *Project Earthrise* at our ninth annual conference of *in*VIVO Planetary Health in 2020. In an age of convergence, this initiative promotes awareness of interdependence on all scales—across the continuum of people, places and planet—and creates opportunities through connectivity by nourishing a diverse community of change. In addition to our established history of robust science [4], *Project Earthrise* aims to normalize more mutualistic, creative approaches to positively influence normative value systems—placing greater importance on spiritual perspectives from all cultures, including Indigenous traditions and practices [5]. It is vital to redefine “progress” and “growth” in more meaningful ways, recognizing that not all innovation is improvement, and placing greater value on deeper wisdom and happiness. While technology may be a vital part of the solutions, it is best applied with more mutualistic value systems as a compass.

*Project Earthrise* seeks to equally consider our social and spiritual ecology as it does natural ecology; to address “broken spirit” as well as “broken systems” manifest in mounting social unrest, hopelessness, and unparalleled adversity [5]. Our agenda seeks to place a higher value on self-development, creativity, and imagination in solving challenges at all scales. This includes greater emphasis on positive assets in health and resilience on all scales (awe, wonder, joy, love, compassion). Normalizing empathy, kindness, hope, creativity, and mutualistic values—the deeper values that unite, empower, and refocus priorities of individuals and groups—also mediates greater social responsibility and environmental concern. Too often, these qualities are devalued or dismissed in scientific discussions, and doing so, we fail to recognize this is part of the problem, despite these being arguably our greatest assets in overcoming our most difficult challenges.

## 2. Reinvigorating Past Inspirations—As We Step Forward

*Project Earthrise* is inspired by one of the most profound moments in modern history. The “Earthrise” photograph, taken on the 1968 Apollo 8 mission, became one of the most significant images of the 20th Century, triggering a profound shift in environmental awareness and the potential for human unity. It provided a “wake up” call on every level, from personal awareness to planetary consciousness. 

Apollo 8 left behind a world in turmoil, war, riots, a pandemic (H3N2), famine, social upheaval, and environmental destruction—with many parallels to our current challenges 50 years later. While focus of the journey was largely on the promise of the moon, it was the unscheduled photograph of the Earth that unexpectedly changed our world. “*We came all the way to the moon to discover Earth*”, reflected astronaut Bill Anders who took the photograph, on the moment that shifted collective human consciousness [6]. This has an utterly profound effect on personal and planetary awareness, and “*…began to bend human consciousness*” [7]. In other words, it was argued that the 19¢ film became the most important part of the multi-billion-dollar project [8], stirring collective imagination, awe, wonder, and a new sense of unity. The resulting frameshift in perspective led to large-scale environmental and social movements and inspired the first Earth Day in 1970. This event had a far broader agenda than it has today, addressing manifold issues ranging from growing poverty, blight, racism, and war, to broad-ranging environmental decay—with exploitative economic systems as the common denominator, stemming from a culture of profit over people, and self-interest over the beauty and mystery of nature [9]. In short, it was a call to address fundamental value systems [10].

This remarkable outward journey had the even more profound effect by turning awareness inward to our own condition and that of our troubled planet [11]—arguably the same kind of shift in consciousness that we need to reignite now, employing all of our “resources” by valuing our humanity at least as much as our technology. 

In an era that also holds much promise, as many groups now focus on the necessity of a “Great Transition”, it is even more important to revisit these values and principles as we seek wiser choices. *Project Earthrise* is our contribution to this important process.

“*We have today the knowledge and the tools to look at the whole earth, to look at everybody on it, to look at its resources, to look at the state of our technology, and to begin to deal with the whole problem. I think that the tenderness that lies in seeing the Earth as small and lonely and blue is probably one of the most valuable things that we have now.*”

Margaret Mead, anthropologist, Earth Day, 1970 [9].

*Project Earthrise* was launched in December 2020, as an ongoing and evolving initiative to reinvigorate and advance this planetary health perspective. Held virtually because of the COVID-19 pandemic, we had the opportunity to record discussions as a resource for others to share on an ongoing basis [12]. Here, we share the initial phase that formed the basis of our ninth annual conference of *in*VIVO Planetary Health.

Building on the diverse experience and foundations of our global network, our agenda underscores the imperative for creative ecological solutions for the challenges we face in all systems and all scales with advancing global urbanization in the digital age—for personal, environmental, economic, and societal health alike. We brought together diverse perspectives from across many dimensions of the arts and the sciences, to explore novel solutions and new normative values.

This reflects our ongoing focus on understanding and improving the complex relationships between human health and planetary health. We seek to emphasize the socio-eco-biological interactions in our living environment (including urbanization, food systems, education, social inequity, climate change, biodiversity loss, and microbial ecology) that impact physical, mental, social, and spiritual wellbeing, together with the wider community and societal factors that govern these. We continue to have a long-range vision that includes trans-generational and “life-course” approaches to disease prevention and environmental restoration.

The meeting celebrates a tremendous network of like-minded people from diverse fields whose interests span from planetary/population/environmental health to microbial ecology/systems biology and the deep biological mechanisms—all aiming to work “symbiotically” to connect traditional “silos” through a more integrated systems framework as we seek to improve personal, environmental, economic and societal health alike. Our emphasis on meaningful collaborations and productive friendships is as important as the data and opportunities we generate. In this way, *Project Earthrise* provides a space for kindred spirits whose work is pathbreaking enough for them to feel at times isolated in their own immediate academic (or other) environments—and enables the kind of global convergence that exemplifies our goals.

Together, we have begun to create a mosaic narrative of diverse perspectives, not just for a clearer picture of possibility, but for the spirit of hope and connectivity we have generated in the process. As we each connect our work to the bigger picture with shared purpose, we empower and magnify potential for change. Below, we share the submitted abstracts, and an open invitation for all readers to watch the on-line proceeding, available through our virtual proceedings [12], and to join in ongoing discussions and activities.

**Figure 1:**
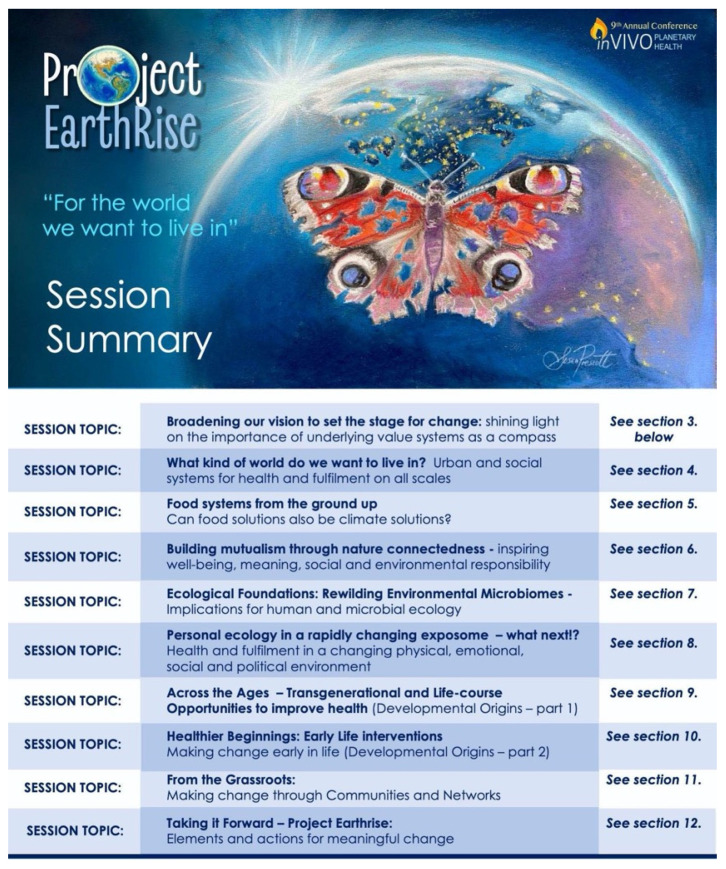
Overview of the sessions presented within the initial *Project Earthrise* discussions at our annual conference (and the corresponding sections of these proceedings).

Our program was designed to capture this mosaic—as an evolving story to underscore interconnections between diverse fields and encourage novel perspective for new collaborations. The overall map for the conference sessions is shown in Figure 1, to assist the reader in navigating and searching the content presented here.

This diverse agenda continues to unfold through ongoing conversations and opportunities for collaborative action—on an evolving basis. We welcome new ideas and new collaborations and hope that the content below and the on-line discussions provide inspiration and opportunity for many.

We encouraged all contributors, no matter the topic, to consider the ways in which their work is relevant to the larger agenda, and to imagine how they would like to see their field evolve in an ideal world. Recognizing that imagining the future is the first step to getting there, we encouraged all to draw on the inspiration of “Earthrise” and consider the fundamental question: “*What kind of world do we want to live in*?”

## 3. Broadening Our Vision to Set the Stage for Change

**Figure 2:**
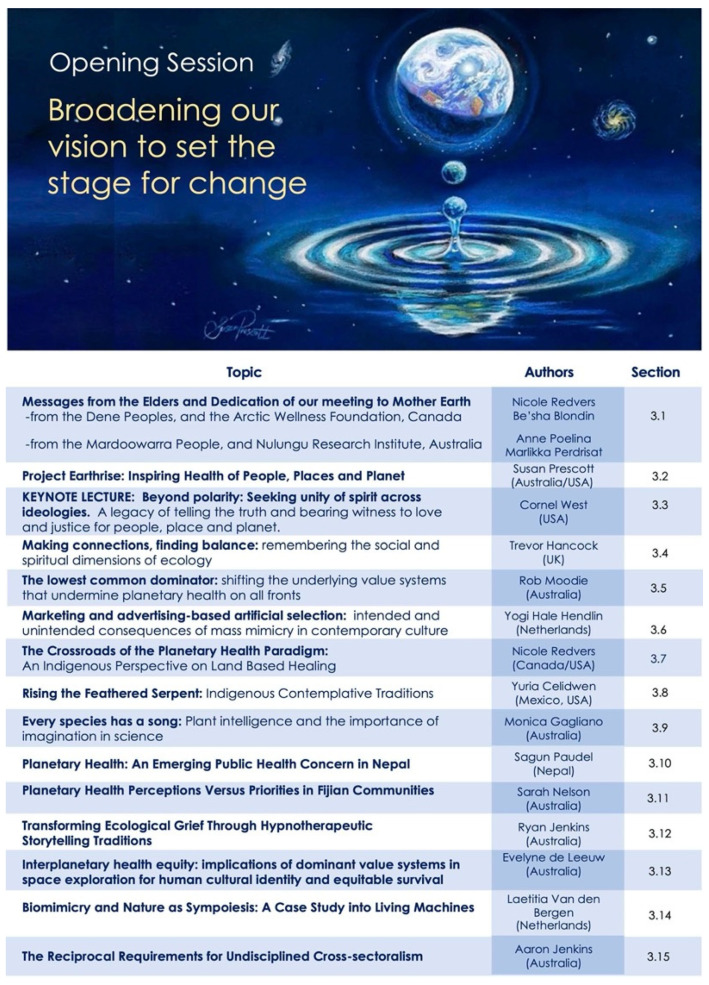
Broadening our vision to set the stage for change. Overview of topics and speakers in the opening session.

Our opening session sets the scene and scope for the meeting and introduced *Project Earthrise* as both the theme of the meeting, and an ongoing collaborative venture for change—for creating convergence of ideologies, actions and inspirations. The abstracts presented below (summarized in Figure 2) capture some of these core elements, and video recordings are also available online [12].

### 3.1. Messages from the Elders and Dedication of Our Meeting to Mother Earth—From Both Sides of Her Equator

**Be’sha Blondin ^1^, Nicole Redvers ^1,2^, Marlikka Perdrisat ^3^ and Anne Poelina ^4^** (^1^ Arctic Indigenous Wellness Foundation, Box 603, Yellowknife, NT X1A 2N5, Canada; ^2^ School of Medicine & Health Sciences, University of North Dakota, 1301 N Columbia Rd Stop, Grand Forks, ND 58202, USA; ^3^ Sydney Law School, The University of Sydney, New Law Building, Eastern Avenue, Camperdown Campus, Sydney, NSW 2006, Australia; ^4^ Nulungu Research Institute, University of Notre Dame Australia, P.O. Box 2747, Broome, WA 6725, Australia).

We were privileged to share wisdom from the Elders of both the Arctic (Dene Peoples) and Western Australia (the Mardoowarra Peoples). As we celebrate the unique beauty and Natural or First Laws of each place, we can also appreciate the shared deeper resonance within the science and values from our very different regions of the world. Each of our collective traditions underscores the importance of balance in all things—including using our minds, heart and spirit in making wise decisions that ensure a healthy environment for the generations that follow.

Natural or First Law stories from all regions teach about ethics, values, codes of conduct and how to live in a civil society. They teach the next generations to work with one mind and one spirit, and the importance of taking responsibility for the way that we live, and work to overcome separation and division. Natural or First Law provides a timeless perspective of our relationship with the Earth—as the past, present and future are fused in this moment—teaching us to care about things beyond what is human, and to respond to protect Mother Earth. Our deep and harmonious relationships within Nature shows that there must also be balance in society and removal of hierarchy. This helps promote accountability, compassion, and responsibility in a life-long journey of meaning and discovery.

We come from a world of “we” not “me” that considers the collective wellbeing of communities, not just for Indigenous Peoples, but all citizens of the world. The oldest living cultures in the world hold ancient wisdom and stories that will greatly benefit many societies in dealing with complexity, and in codesigning solutions going forward—but only when our rights and the rights of Mother Earth are acknowledged.

Wisdom, love, and ethics of care can be gifts from Indigenous Peoples to the world with a deep respect for our self-determination and sovereignty—symbolizing that we must walk hand in hand together as one people for the health our planet.

### 3.2. Project Earthrise: Inspiring Health of People, Places and Planet

**Susan Prescott** (School of Medicine, University of Western Australia, the ORIGINS Projects at Telethon Kids Institute and Perth Children’s Hospital, Perth, WA, Australia; Department of Family and Community Medicine, Center for Integrative Medicine, University of Maryland School of Medicine, Baltimore, MD, USA; and The Institute for Integrative Health, Baltimore, MD 21231, USA).

COVID-19 has precipitated sudden and unprecedented change, underscoring the interdependence of all systems and the direct connections between personal and planetary health. This acute event has drawn new attention to many chronic problems and revealed how rapidly priorities and behaviours can change. It has also created a greater imperative to shift our trajectory towards a more sustainable and resilient future. Drawing on the same sense of awe and unity that shifted human consciousness when we first saw our home across the void of space in the “Earthrise” image, *Project Earthrise* provides an opportunity to reassess collective values, priorities, our sense of self and community on all scales—in the same spirit of the first Earth Day in 1970.

The *Project Earthrise* agenda creates a health narrative across all scales, emphasizing multidimensional interactions and bi-directional relationships for flourishing of people, places and planet. It seeks to place a higher value on self-development, creativity, and imagination in solving challenges at all scales. It recognizes that the future of health depends not only on reducing adversity, but restoring positive and protective effects of nature, biodiversity, traditional foods, community, and purpose—the elements that are often neglected, undervalued and underestimated. This underscores the importance of grassroot efforts in every sense—from restoring foundational ecosystems, such as microbial ecology that underpins all life (including our own), to community solutions that bring out the best of human “nature” for multi-dimensional personal and collective benefits. Starting “at the roots” also means starting at the foundations of life, recognizing the fundamental importance of a developmental and transgenerational vision to life-long physical, emotional health, attitudes and behaviours.

For this, we encourage work in the space “between siloes” of expertise, and harness the spirit of connectivity, community, to promote collaboration, purpose and belonging. We aim to bring together diverse expertise and rich perspectives to create a shared change narrative, recognizing that seeing a problem from more angles brings solutions into clearer focus. Together with robust science, this agenda emphasizes the importance of cultural expression, artistic creations and narrative in linking the health of people, places and planet.

### 3.3. KEYNOTE LECTURE: Beyond Polarity: Seeking Unity of Spirit across Ideologies. A Legacy of Telling the Truth and Bearing Witness to Love and Justice for People, Place and Planet

**Cornel West** (Professor of the Practice of Public Philosophy, Harvard Divinity School, Cambridge, MA 02138, USA; Professor Emeritus at Princeton University, Princeton, NJ 08544, USA).

No abstract available: a recording of Dr. Cornel West’s inspirational Keynote Lecture is available for viewing through our online proceedings [12].

### 3.4. Making Connections, Finding Balance: Remembering the Social and Spiritual Dimensions of Ecology

**Trevor Hancock** (Retired Professor, School of Public Health and Social Policy, University of Victoria, Victoria, BC V8W 2Y2, Canada).

Perhaps the central problem of our time is that we have lost both our connection to nature and our sense of perspective with respect to nature. We face massive and rapid human-created global ecological changes—The Anthropocene—driven by the combination of population growth, economic growth and the growth in the power and ubiquity of our technology. We have come to see ourselves as both separate from and superior to nature and other living things, over which we have a (God-given) dominion. We can no longer see the stars at night, we do not know our place in the universe. 

Of course, it was not always so. Until comparatively recently—and still in many Indigenous cultures—the Earth was a living being, our Mother, and other species were our relations. Interestingly, James Lovelock’s Gaia Theory and Earth system science tells us the same thing: The Earth “behaves as a single, self-regulating system comprised of physical, chemical, biological and human components”. We are part of the Earth system; in fact, E.O. Wilson’s Biophilia hypothesis says that because we evolved in nature, we have an innate need for nature and subconsciously seek connections with the rest of life.

We are also connected to all other life through our DNA. Not only do we share 99.9% of our DNA with other humans but 99% with chimps and bonobos and 98% with gorillas. We share 84% with dogs, 60% with the chickens we eat and with fruit flies, and even 26% with yeast and 15% with mustard grass. Moreover, we are connected to all the people, animals and plants that came before us through the atoms we inhale or ingest, and which once were part of their bodies. In short, we are all deeply connected to and entirely dependent upon the great web of life which sustains us in many ways.

The ultimate connection, perhaps, is to the universe. All our atoms—yours, mine, all other living things, all the materials of the Earth, the solar system and beyond—have a common origin: We are all star stuff, forged in the heart of collapsing and exploding stars. I find it a comforting thought that when my time is done, I will become recycled star stuff, my borrowed atoms living on as part of the great web of life, but if you cannot see the stars at night, you cannot see where we came from. 

Suggested reading: Amsterdam Declaration on Earth System Science, 2001 (http://www.essp.org/ accessed on 1 August 2021).

### 3.5. The Lowest Common Dominator: Shifting the Underlying Value Systems That Undermine Planetary Health on All Fronts

**Rob Moodie** (Professor and Deputy Head of School of Population and Global Health (MSPGH), University of Melbourne, Parkville, VIC 3010, Australia).

The problems I deal with are the problems of unhealthy commerce. These are the “other” twenty-first century pandemics—of preventable cancers, heart attacks and strokes, diabetes and lung diseases across the globe. They are driven by the Supra National Corporations that produce, promote and market unhealthy commodities. The values that underly unhealthy commerce are the accumulation of money, power and influence derived from the exploitation of consumers and resources. This produces giant power asymmetries where governments struggle to find any forms of national economic and social balance. Of the world’s 100 largest economies, more than 70 are now corporations. Power is shifting away from nation states to the Supra National Corporations. Yet, we know that what produces the best social, economic, health and wellbeing outcomes are balance and equity:

- *Balance* in the way we lead our lives and the way we use resources;
- *Equity* resulting in increasing life expectancy, literacy and numeracy and trust, and decreasing infant mortality, mental illness, obesity, imprisonment levels and homicide rates.

Those interested in planetary health will inevitably be in conflict, particularly when we know how dramatically some of these corporations are harming our planet.

How do we resolve conflict? We can choose collaboration—where we have a very high level of assertiveness (in this case about planetary health) combined with a very high level of cooperation (in this case for-profit enterprises making sustainable profits). We can support, encourage, and work with for-profit companies that want to balance profit and purpose. There are some! But this approach will not work in many cases. This is where we have to assert ourselves and compete. This is where evidence, relentless advocacy, community mobilisation and policy change work synergistically. 

And back to the theme of *balance*. We need to balance ourselves.

To achieve the values that drive a healthy planet—loving kindness, compassion, empathy, sympathetic joy and liberation from our egos, we need to commit time, effort and practice to nurture ourselves. We need to look after ourselves. This generates hope—and hope is an essential element we require for a healthy planet.

Can we imagine a future where it becomes the norm for those born with advantage, privilege and entitlement to become major drivers of equity, fairness, love and respect, rather than further entrenching inequity? Healthy commerce can and must play a central role in planetary health. Imagining is a wonderful way we give structure to our hope. It gives us the scaffold on which we can generate and nurture our hope.

### 3.6. Marketing and Advertising-Based Artificial Selection: Intended and Unintended Consequences of Mass Mimicry in Contemporary Culture

**Yogi Hale Hendlin** (Erasmus University College, Burgemeester Oudlaan 50, 3062 PA Rotterdam, The Netherlands).

From the perspective of an environmental philosopher and public health policy scientist, this talk examines the intended and unintended consequences of advertising in contemporary culture. The thesis holds that too much advertising based on short-term private profit, rather than the common good, is (de)sensitizing us in ways leading to both human devolution and environmental degradation.

“Artificial selection” (advertising) *prescribes* selection according to vested interests in a particular end goal. This type of selection can be contrasted with the processes of natural selection which *proscribe* without a predetermined endgame—it is open-ended, but limits excess through natural constraints to allow beauty to flourish. Artificial selection capitalizes on the heuristics of the human brain (cognitive shortcuts) to allow automatic processes and avoid cognitive overload in daily function. These instinctive behaviours are triggered by shapes, colours, and objects, which may suggest threat, security or status. Advertising creates artificially pumped-up “supernormal stimuli” which play on these heuristics to promote unreflective behaviours which lead to product consumption.

The environmental fallout of excessive consumption is obvious and are the health consequences, with many of the unhealthiest products (such as soda drinks and ultra-processed foods) specifically targeted to the poorer members of societies. Advertising in general amplifies dissatisfaction and resentment, particularly by those who cannot afford more elite products—as 80% of advertising is targeted at the top 10% of earners. This leads to a “blackhole of self-doubt” and growing mistrust of external stimuli. People start to doubt the world around them, and eventually inside them, in growing existential insecurity. There is growing isolation from world and from ourselves—as we forget who we are without these commodities. It spreads ignorance, inculcates separation and gaslights our own bodily wisdom. In all this, those least equipped to defend against their tactics are the most susceptible. 

The undermining catch of this “catch-22” is that “by trying to *seem*, we get further away from where we want to *be*”. Thus, advertising is a form of “soft pollution” that is appropriating our attention and canalizing our experiences—but the wider consequences of the resulting changes in mass behaviour incur a mounting personal and planetary toll. We have the cognitive ability to step back and see what is really going on and begin to deconstruct these artificially constructed norms that are affecting the health of people, places and planet. 

### 3.7. The Crossroads of the Planetary Health Paradigm: An Indigenous Perspective on Land Based Healing

**Nicole Redvers** (School of Medicine and Health Sciences, University of North Dakota, Grand Forks, ND 58202, USA).

The Planetary Health movement is gaining significant traction in and around both universities and organizations in North America and abroad. Planetary health is by no means a new innovative discipline, as often is suggested in academic circles, but a deeply rooted connection that all of our Indigenous ancestors had to the land as a medicine and healing place. To understand the planet and its functions is to understand oneself in the Indigenous worldview.

Our Elders predicated the awakening of hidden diseases trapped in permafrost, to the tilting of the planet’s axis due to the polar cap melt, to the sinking of the ground due to sumps forming from permafrost melt and talked specifically about these changes that were coming. From our Elders having a pure reverence for the land, reality became clear through the lessons given by the water, animals, the sky, and through ceremony passing the responsibility to us to learn from those lessons and enact change for the progression of humanity. 

We are at a crossroads as a human species in regard to the state of our mother earth. There has been increasing calls for Indigenous involvement and leadership around the globe at the grassroots level to support the health and stewardship of our planet. Indigenous peoples have a large role to play in this leadership process. With organizational structures being put in place to ensure the next seven generations have the opportunity for a healthy life, we must ensure the traditional ways of knowing of health and disease are honoured in a good way which starts from our original teachings. The land healing relationship is our connection back to balance.

### 3.8. Rising the Feathered Serpent: Indigenous Contemplative Traditions

**Yuria Celidwen** (Indigenous Scholar, Speaker, Advocate for Indigenous Rights and the Rights of the Earth, New York, NY 10017, USA).

The emergent field of contemplative studies-and its manifestation as the mindfulness movement-draws inspiration mostly from Buddhist-derived meditation practices, texts, and principles. These practices have been largely adopted in the West as a popular source for health-related benefits mainly related with stress reduction and focused attention. The benefits of these practices for only physical and psychological wellbeing do not represent the higher resolve of realization of spiritual development and the structure of ethical goals. On this note, more recently, after undergoing secularization, the popularized practices in the West have expanded their focus to include compassion and loving–kindness practices and the enhancement of prosocial behaviour. While these practices have been pertinent and effective, they only represent a very selected variety of practices across Asian traditions. Moreover, although the field has engaged with other religious traditions and their practices of contemplation, these studies have been scarce.

Indigenous religious traditions have likewise engaged in a diversity of contemplative practices with similar spiritual impact, and physical and psychological benefits. With the intention and motivation of expanding the circle of inclusion, I have been working to include Indigenous religious traditions in the conversation of contemplative science and practice. From my work on Indigenous contemplative science, I developed my thesis on the earth-based experience of the Ethics of Belonging. This ethos engenders conscious social responsibility for self, community, and environment. It implies a renewed sense of order (cosmos) through a system of integration of ecological and ethical awareness of individual and collective, material and subtle, adaptive and interactive, and meaningful relational purpose within a community. Within this work, I examine how our personal stories relate to cultural accounts that can transform our identities and the social and racial injustices of our times. This proposal is intended to invite dialogue among contemplative Indigenous religious practices and to reclaim Indigenous traditional wisdom and revitalize Indigenous voices as similar keepers of profound sophistication and variety. I am certain that this dialogue not only creates inclusive and diverse ways of knowing, but it will enrich the scope of impact of these contemplative practices to engender social change for social and environmental justice.

### 3.9. Every Species Has a Song: Plant Intelligence and the Importance of Imagination in Science

**Monica Gagliano** (Research Associate Professor in Evolutionary Ecology at Southern Cross University, Lismore, NSW 2480, Australia).

No abstract available.

### 3.10. Planetary Health: An Emerging Public Health Concern in Nepal

**Sagun Paudel** (President, Public Health Youth Society of Nepal, Kaski 33700, Nepal).

Background: Natural resource degradation, climate change, and global warming have become widespread concerns and Nepal is no exception. After a long decade of political instability, Nepal is in the transition phase of development and industrialization; the utilization of natural resources, expansion of roadways, construction of large hydro powers, the establishment of big industries are gradually increased. Nepal lies in between two larges economically developing countries; China and India. The industrial development of the neighbour country, directly and indirectly, effects on the ecosystem of Nepal.

Objective: The objective of this paper is to analyse the environmental situation, natural resources depletion and consequences in human health in the context of Nepal. Method: To prepare this paper, journals, articles and national reports were retrieved and analysed to prepare a manuscript. This paper was prepared by using various secondary data sources available on internet web pages, journals, government reports, and articles. 

Findings: More than 80% of Nepalese depend on forests for livelihoods and 66% of the total gainfully employed population is engaged in natural resource sectors. Eighty percent of the population are at risk with the VPDs and NTDs which are only endemic in the few districts, the population at risk will likely increase in the future due to the shifting of disease vectors into highland areas. The ambient air appears to be polluted with high levels of PM2.5 and NO_2_. These indicators reflect that there is a possible consequence to increase the mortality of respiratory illness mainly on COPD and pneumonia.

Conclusion: Nepal is at risk of suffering from the consequences of global warming, carbon emission, and other environmental threats. The protection of the environment and ecosystem should be initiated through collective action, awareness, and implementation of activities in the frame of planetary health. It should be introduced within the regular program.

### 3.11. Planetary Health Perceptions Versus Priorities in Fijian Communities

**Sarah Nelson, Joel Negin, Seye Abimbola, Jacqueline Thomas and Aaron Jenkins** (University of Sydney, Camperdown, NSW 2006, Australia).

Introduction: Rural Fijian communities have close relationships with land and water and depend on it for their livelihoods and security. Correct water management is a top priority for Fiji and planetary health at an individual community level and at the wider ecosystem health of the catchment level. Understanding the perceptions and priorities of water resource management for planetary health allows for action to build maintain climate-resilient planetary health systems and for communities to understand changing priorities and linkages.

Methods: A mixed methods approach was used. Semi-structured interviews at community, provincial and national were used to understand what individuals view as the water priorities for planetary health. Water quality testing and mapping were used to determine if their perceptions of planetary health priorities were correct.

Results: Planetary health perceptions were found to be to be different through qualitative and antiquate data. Water access was found to be intermittent; often dirty after heavy rain and communities were aware of these issues. Community practices were found to cause issues for ecosystem health particularly through alteration of water quality. Communities thought current practices and alternative water sources were usable, however, water quality testing revealed the impacts of land use, farming and water management practices lead to contamination of water. Community natural resource management varied amongst communities. Community practices impacted the wider ecosystem, at the catchment level and sub catchment level. 

Conclusion: Planetary health perceptions versus priorities are important to understand at the community level as the only one part of a nested system that and impacts the wider ecosystem. Creating appropriate understanding of community planetary health actions, can help create a healthier ecosystem, reducing disease, improving farming techniques, land use practices and improving water quality.

### 3.12. Transforming Ecological Grief through Hypnotherapeutic Storytelling Traditions

**Ryan Jenkins ^1^ and Aaron Jenkins ^2,3^** (^1^ Hypnotherapy in Barcelona, 08024 Barcelona, Spain; ^2^ University of Sydney, Camperdown, NSW 2006, Australia; ^3^ Edith Cowan University, Bentley, WA 6027, Australia).

Psychotherapists worldwide are increasingly documenting a sense of loss and grief attached to the systematic dismemberment of the natural world. This inner anguish is referred to as ecological grief, in relation to either experienced or anticipated ecological losses due to acute or chronic environmental change. Often unmeasured, and largely immeasurable, this mental health condition is a pivotal psychological reality of the Anthropocene. While we rely on modern science to shed light on our planetary condition and provide guidance on improving our relationship with the biosphere, our motivation to act is unlikely to stem from the largely uncomforting scientific mythos. Storytelling, however, is evolutionarily advantageous.

Stories with humans in a central role, preserve our mystery and dignity, helping pass on valuable information in memorable, repeatable and actionable forms thereby enhancing our chances of survival. We draw upon fields of Ericksonian hypnotherapy, ecopsychology, ethnomusicology and medical anthropology to create a novel therapeutic storytelling practice specifically designed to help acknowledge, release, legitimise and transform our diverse and often repressed emotional responses to our rapidly changing planet.

Our model therapeutic practice is derived from storytelling traditions of Papua New Guinean hunter–gatherers, West African and Amerindian shamanism, folkloric and contemporary dystopian fiction, Scandinavian cinema and modern musical traditions. We provide a framework for transforming ecological grief using therapeutic methods of indirect hypnosis and neuro–linguistic programming, narrated in a story and augmented by visual and audio media.

We contend that hypnotherapeutic storytelling can help transform natural feelings of ecological grief from a dulled, frustrated inertia, caused by forces of market capitalism and the Palaeolithic architecture of our minds, into the seeds of ecological awareness and social action.

### 3.13. Interplanetary Health Equity: Implications of Dominant Value Systems in Space Exploration for Human Cultural Identity and Equitable Survival

**Evelyne de Leeuw** (Centre for Health Equity Training, Research and Education CHETRE, Ingham Institute, 1 Campbell Street, Liverpool, NSW 2049 Australia).

Some believe that traveling to the stars (or, rather, moons and planets in our own solar system) is a futile endeavour, and that we should first and foremost focus on our own planet(ary health). As true as this is, and as depressing the hunt for interplanetary mining and colonisation may appear, it is very much on the books of global stakeholders. These not only include nation–states (such as the UAE, China, India, Japan, European Union and USA) but increasingly private industries. We should be concerned about the impact of these efforts on health and wellbeing equity on Planet Terra. Spending billions if not trillions (whether or not garnered from state sources through taxpayer schemes or from private capital through profit and philanthropy) on space exploration is at the detriment of investments in sustainable food systems, water management, healthcare and health protection, etc. A warning should be sounded. However, there is a phenomenon that is potentially even more challenging, even if planetary health equity is assured. That is interplanetary health equity. The Outer Space Treaty (entered into force in 1967) forbids colonisation or exclusive rights to anything beyond Earth’s atmosphere and regards space travellers as envoys of humankind. However, in a move that possibly further illustrates the Unites States’ retreat from the global order of nations (as much as its withdrawal from UN agencies such as WHO and bullying of others such as UNESCO), its National Aeronautics and Space Administration (NASA) has drawn up the “Artemis Accords”. The world has been invited to join NASA in this—as if UNOOSA is incapable of implementing globally agreed on treaties. Under these Accords space may be free, but anything that is mined and produced out there is not. This is a severe threat to humankind and our equitable survival.

### 3.14. Biomimicry and Nature as Sympoiesis: A Case Study into Living Machines

**Laetitia Van den Bergen** (Erasmus University College, 13011 HP Rotterdam, The Netherlands).

Providing water in acceptable quality while ensuring its future availability constitutes one of the major challenges of our century. While Western countries’ water infrastructures have solved sanitary issues, they are at odds with the contemporary sustainability paradigm. Designers and engineers have therefore turned to biomimicry, moved by the assumption that taking nature as a model, a measure, and a mentor will produce sustainable devices. However great is biomimicry potential for sustainable design, it is under-exploited because of the field’s lack of philosophical conceptualization. This work uses both philosophical speculation and scientific literature to investigate how biomimicry should be practiced to generate truly sustainable technologies, meaning technologies that create conditions conducive to Life. 

Philosophical speculation suggests that the paradigm opposing Nature to Humanity in which scientists currently operate restrains biomimicry ability to achieve sustainability and humans’ capacity to understand and respond to the ecological crisis. It was proposed that biomimicry should be performed according to a paradigm that conceives humans in their entanglement with non-humans. By following a bio-inclusive and bio-synergetic approach aimed at fulfilling our needs while serving the ones of other species and systems, biomimetists could work with Nature to solve challenges we have in common—i.e., polluted water.

Living Machines (technologies that emulate wetlands form, process, and system) were proposed as an admirable example of the application of biomimicry. My scientific literature review suggests that using constructed wetlands reduces not only the costs and outputs of wastewater treatment but also energy, material, and water demands. More, it favours social sustainability.

Overall, this study provides an interdisciplinary framework to inform a more sustainable practice of biomimicry. Propositions for further research include the application of bio-inclusive ethics as a framework guiding the transition to a circular, bio-based economy, and the clarification of constructed wetlands’ role in the latter.

### 3.15. The Reciprocal Requirements for Undisciplined Cross-Sectoralism

**Aaron Jenkins ^1,2^, Anthony Capon ^3^ and Pierre Horwitz ^2^** (^1^ University of Sydney, Camperdown, NSW 2006, Australia; ^2^ Edith Cowan University, Bentley, WA 6027, Australia; ^3^ Monash University, Clayton, VIC 3800, Australia).

Modern society is gradually appreciating that deep understanding of the ecological foundations of health can help guide global sustainability. Our current discourse and action, however, rarely engages meaningfully across sectors or allows the undisciplined space where (all) disciplines are involved but none takes control.

To facilitate a broad planetary agenda, we sought to understand the reciprocal requirements for undisciplined cross-sectoralism at individual, organizational and systemic levels. We convened leaders in indigenous health, infectious disease, urbanization, nutrition, immunology, aquatic and landscape ecology, in a roundtable discussion to explore collaborations to connect environmental change to human health outcomes. We focused discussion on the personal and organizational qualities needed to translate discourse into action.

We agreed that individuals who excelled at cross-sectoral and undisciplined approaches see themselves as life-long learners, usually with a particular interpersonal skill set and a specialist discipline to which they are not necessarily wedded; they are willing to learn the language of others, and are careful listeners, often at the expense of interpreting problems in the ways in which they have been (res)trained. They help link the individual to the organizational through place-based, empathetic, and iterative processes including those most affected, while addressing critical power dynamics in co-design of solutions. Organisations designed well for cross-sectoral collaboration have a shared vision, are influenced by other organisations in a reciprocal way, and more likely to adopt a horizontal business model, without being insular. Systems designed well for cross-sectoral collaborations provide incentives, promote attitudes and capabilities that have empathy for the whole, and encourage organisations and individual behaviours that are supportive not competitive.

The nature of transformative action is not about efficiency. It is metamorphic and participatory, where processes and outcomes are blurred and not predetermined. It is subversive of disciplines and sectors where these structures operate to exclude broader societal agendas.

## 4. What Kind of World Do We Want to Live in? Urban and Social Systems for Health and Fulfilment on All Scales

**Figure 3:**
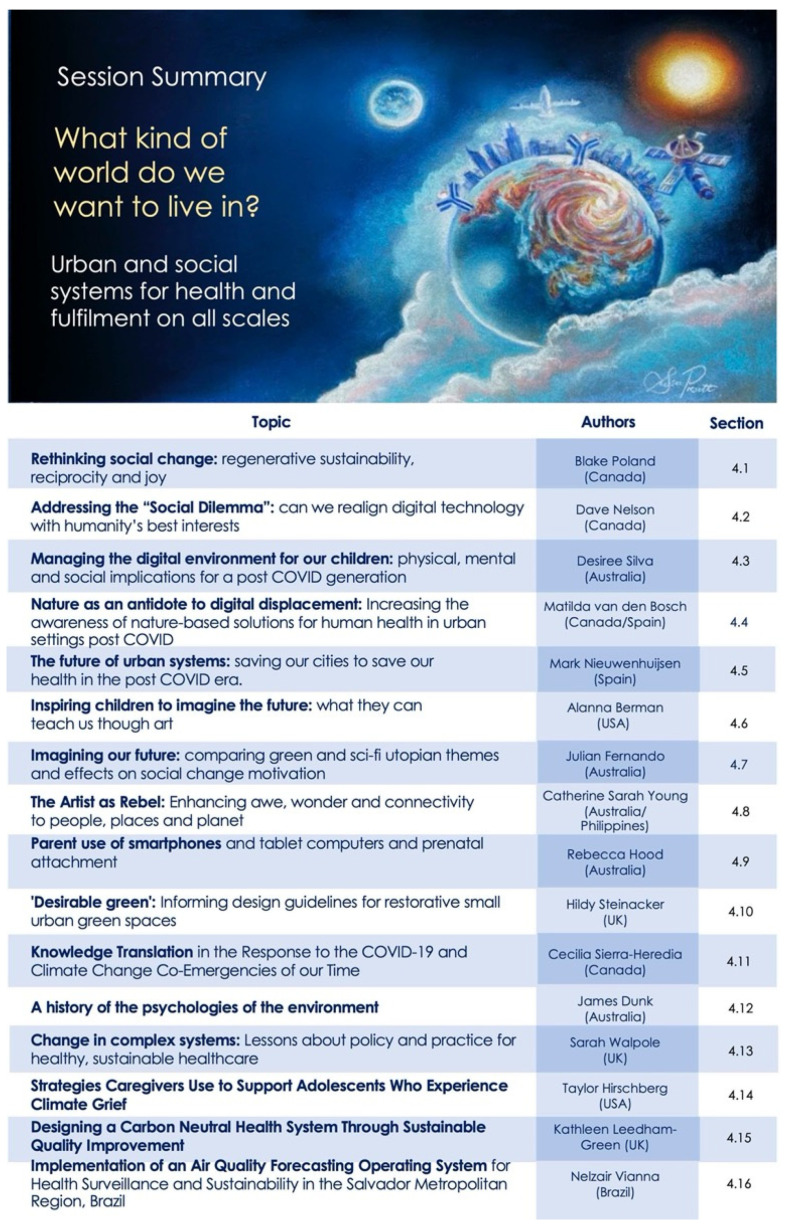
What kind of world do we want to live in? Overview of topics and speakers in the session on urban and social systems for health and fulfilment.

This session explores the challenges of changing social and urban environments—including increasing immersion and dependence on digital environments, as a rapidly emerging factor in “nature disconnection”, especially in younger generations. We also examine the social changes that may be needed to promote healthier, equitable, thriving systems that support flourishing across scales of people, places and planet. This includes pathways to efforts to promote social cohesion and connection to nature through artistic ventures and urban green initiatives. The topics are summarized in Figure 3, and recordings are also available [12].

### 4.1. Rethinking Social Change: Regenerative Sustainability, Reciprocity and Joy

**Blake Poland** (Dalla Lana School of Public Health, University of Toronto, Toronto, ON M5T 3M7, Canada).

Emerging threats to planetary health and health equity, including climate change, ecological degradation, resource depletion, energy insecurity, and widening socio-economic disparities, are converging in ways that some now predict could bring down civilization as we know it within the lifetimes of many alive today. Risk management, the dominant system response, is not up to the challenge, and indeed, by seeking to predict and control the negative outcomes of current systems and prevent or bounce back from adversity is tilted in favour of maintenance of the status quo. Something more “radical” (as in getting to the roots of the crisis) is required. In this presentation, three promising directions for reimagining sustainability are proposed, from a landscape of possibilities.

First, decolonizing ourselves from the dominant western paradigm that naturalizes inequality, “meritocracy”, competition, domination, scarcity mentality, a story of separation, and that imagines sustainability as a greening of business as usual. Fortunately, there are many other non-dominant knowledge traditions we can draw inspiration from to help us see that things can be other than we currently imagine them, from critical and progressive traditions at the margins in the Global North (degrowth, political ecology, ecofeminism, deep ecology) to Indigenous ways of knowing and Global South epistemologies. We are brought to the question: what if the sustainability crisis is not a technical problem (as we are often led to believe) but rather a relationship problem, in the sense that we’ve fallen out of right relationship with ourselves, each other, and the more-than-human world?

A second promising direction for reimagining sustainability follows: embracing animism and a relational world view, where all life is seen as animate, sentient and possessing agency and spirit, a world that is fully alive, in contrast to one that modern Western culture sees as a world of “things” and “resources” at our disposal.

Third, we are invited to “unleash a pandemic of positivity”, to conjoin a clear-eyed realism about the current state of affairs with a clear vision of “the more beautiful world our hearts know is possible”, reflecting the power of vision and dreams to call a better future into being (not only railing against what we do not want). What if, to quote Rob Hopkins from the Transition movement, “if it’s not fun, it’s not sustainable”? We are invited into the sweet spot at the intersection of what you love, what you are good at, and what serves the world, as your unique contribution to realizing the momentous transition underway that Joanna Macy calls The Great Turning.

### 4.2. Addressing the “Social Dilemma”: Can We Realign Digital Technology with Humanity’s Best Interests

**David H. Nelson** (Independent Researcher, Woodstock, ON N4S 6Y9, Canada; *in*VIVO Planetary Health, West New York, NJ 07093, USA).

Over the last decade, international researchers have been investigating the relationships between excessive screen time (e.g., television, computer, smartphone) and social media use (e.g., Facebook, Twitter, Instagram, TikTok) and human health, particularly in children. The global pandemic has been associated with massive increases in screen time—up by over 50% in children in North America. This has increased the urgency with which researchers must understand the consequences of screen time, and the ways in which it might promote, or detract, from health at scales of person, place and planet. Here, the discussion focuses on the ways in which screen time, and social media in particular, influences arousal within the limbic system and the brain’s reward pathways, and compromises sleep. Behavioural solutions, those promoting judicious use of screen time, are offered.

### 4.3. Managing the Digital Environment for Our Children: Physical, Mental and Social Implications for a Post-COVID Generation

**Desiree Silva** (University of Western Australia, Nedlands, Western Australia 6009, Australia; Joondalup Health Campus, Western Australia 6027, Australia).

There has been rapid change in digital technology and use since the iPhone was first developed in 2007. The use of devises at an early age are related to parental use. In Australia, 9% of infants (<2 years) and 33% of preschool children (3–6 years old) own their own mobile/tablet device and use it 2 h and 4 h/day respectively. This has led to increasing concerns around how this may be influencing patterns of early childhood development in this crucial period of life. 

In the early postnatal period, there are already concerns that increasing parental screen time is affecting eye contact and bonding. Similarly, there is also an increasing tendency to use mobile devises as “babysitters” to occupy, distract and quiet behaviour of babies and children for prolonged periods. This reduced personal social contact may be contributing to social and emotional immaturity—including increasing anecdotal observations of delay in infant social smiling by paediatricians and child health nurses. 

While there have been some benefits of early electronic devise use for family connectedness, especially during the COVID-19 pandemic, long-term studies are needed to determine if this has been out-weighed by the delayed speech, motor and social development identified during this period. In adolescents, the need to be connected to the digital world is also increases, as families struggle to deal with the associated adverse effects of excessive electronic use—including obesity, sleep insomnia, irritability, poor self-regulation, inattention, anxiety, depression and “digital displacement” of traditional in-person socialisation. Excess digital use is likely to displace outdoor play and relationships with nature, with additional adverse—also essential for physical, mental and social wellbeing. 

While there are Australian guidelines for screentime use, few families achieve this. There is still a confusion, lack of awareness, or denial of potential detrimental effects of excessive electronic use on social and emotional development. Those families struggling to control device experience guilt as this becomes increasingly challenging.

This is one important focus in The ORIGINS project. This is a landmark birth cohort in Western Australia aimed at addressing many of these contemporary challenges. The study commenced in 2017 and will follow 10,000 families for 5 years. Embedded in this cohort platform, there are a number of specific studies, including the impact electronic use and interventions to address this—including strategies to increase nature connectedness in families. We are working with local groups, such as Nature Play WA to promote a new approach which includes ways to reduce, replace and balance digital devices especially in children who have sleep and behaviour issues.

We hope the results of this study will help shape policy on mobile technology use in young children especially in the post COVID-19 period.

### 4.4. Nature as an Antidote to Digital Displacement: Increasing the Awareness of Nature-Based Solutions for Human Health in Urban Settings Post COVID

**Matilda van den Bosch** (The University of British Columbia, Vancouver Campus, V6T 1Z4, Canada).

Over the last decade, research on human health impacts of urban natural spaces has rapidly developed. An increasing number of epidemiological studies demonstrates direct and indirect health benefits by exposure to urban green and blue spaces. While these studies have significantly contributed to confirming associations between nature exposure and various health outcomes, a number of challenges remain before evidence on mechanisms and causality can be established. These challenges range from uncertainty about optimal exposure measures to what physiological effects can be expected from nature contact. Nevertheless, the side effects of “urban green interventions” are limited. By also taking co-benefits, such as increased biodiversity and climate change mitigation from urban natural spaces, into account there seems to be little to lose from a policy perspective by advocating for more natural spaces in our cities, although the evidence is still insufficient. This presentation provides an outline of the current evidence level and present research challenges in on-going studies around urban nature and human health. It will also discuss the prevailing discourse around pathways and mechanisms. Finally, the goal is to initiate a debate around opportunities and risks with taking a “nothing-to-lose” approach to urban green initiatives across the world. 

### 4.5. The Future of Urban Systems: Saving Our Cities to Save Our Health in the Post-COVID Era

**Mark J. Nieuwenhuijsen** (Director of Urban Planning, Environment and Health, ISGlobal, 08003 Barcelona, Spain).

Over 50% of the world’s population is living in cities and this is expected to rise to 70% over the next few decades. Cities are society’s predominant engine of innovation and wealth creation, but also main sources of pollution, and disease. Partly due to poor urban and transport planning, or the lack thereof, we have cities that are too car dominated. All the urban planning in the world seems for cars; people do not matter. This has led to high air pollution and noise levels, heat island effects and lack of green space and physical activity that are all detrimental to health. For example, a recent health impact assessment in Barcelona estimated that 20% of premature mortality was due to urban and transport related exposures. 

The COVID-19 has shown the importance of public space in cities and cities have started to transform their public spaces by for example increasing cycling lanes and creating low traffic or car free areas. Furthermore, more green space is being introduced as there is a real need. COVID-19 has been an accelerator in many ways. This talk will review the latest developments on urban and transport planning pathways leading to low carbon, liveable and healthy cities. 

Electric cars and/or autonomous vehicles have been mentioned as possible solutions, but they are unlikely to be, and solutions need to be sought elsewhere. A new long-term visioning of healthy urban future is needed that bring health, sustainability and liveable at the forefront of urban and transport planning. Systemic approaches to the current problems and a shift away from our grey car centric cities towards cities with more public and active transportation and green space are urgently needed. New urban models such as car-free cities, the 15 min city and the Superblocks are urgently needed. Collaboration between e.g., urban and transport planners, environmentalists and public health professionals is essential to create carbon neutral, liveable and healthy cities.

### 4.6. Inspiring Children to Imagine the Future: What They Can Teach Us through Art

**Alanna Berman** (The Institute for Integrative Health, Baltimore, MD 21231, USA).

Art provides a valuable avenue for children, and people of all ages, to be part of global conversations that celebrates our planet, as well as efforts to overcome our challenges. Creativity is also an invaluable tool in expressing feelings, seeing the “bigger picture” and finding personal and/or shared hope at times of stress. I am an artist and art teacher working in a public elementary school just outside of Baltimore, Maryland in the United States, and this was an opportunity for me to share an art project designed to help our students cope with the events of 2020—including their work and their thoughts.

With the COVID-19 pandemic, we, as many other schools, transitioned to a virtual learning environment, and many students and families faced the challenges of social isolation. Our school is highly diverse, with students’ families from all around the world across a wide socioeconomic range. Anxiety mounted as the pandemic continued and events, such as the death of George Floyd, amplified social unrest across the USA and the world. Artistic creativity was an important avenue for my students to process these significant events and share their feelings.

Inspired by the concepts behind *Project Earthrise* (Logan et al., 2020), I created an inspirational video to spark the students’ imaginative thinking and to help them express their emotions and aspirations. Drawing on the awe and wonder of the Earthrise photograph taken by the Apollo 8 astronauts, and how this had inspired new awareness and new possibilities at the time, I encouraged them to draw from their imaginations to create a picture of what a better world might look like—and what matters most to them. 

A number of key themes emerged from work of our young artists and their explanations—ranging from environmental and social justice concerns, to sharing toys and playing with their sister. Even at the age of 8 years, many artists expressed deep caring for nature. Along with frequent depictions of vibrant nature scenes, they included explanations of why trees are important, with comments such as “grow trees, save the world”. Even those who depicted the negative impact of humans on the planet also depicted hope and desire for change, with comments that we need to “change how humans and animals live together and how animals are going extinct due to the population taking over their homes and taking down forests”. Another emergent theme was the desire for more caring communities—a general desire for more “love” and “no bad people”; a wish for more unity as “people of all colours and nations, standing together, working together proudly” and kindness “if someone gets knocked down you help them back up”. Importantly, the very process of reflection, creation and sharing also generated hope and positivity with comments such as “drawing makes me happy”.

Significantly, both children and their families (many experiencing great hardship) expressed profound gratitude in the knowledge that their art and their thoughts would be shared with the international *in*VIVO community. It was meaningful and empowering for them to feel connected to something bigger in this way.

While anecdotal, this illustrates the personal and collective value of creativity and imagination in sharing inspirations and creating hope, and wonderful opportunities to give children a voice in important conversations—because it is ever true to say that children are the future.

Suggested reading:

Alan C. Logan, et al. Project Earthrise: Inspiring Creativity, Kindness and Imagination in Planetary Health. Challenges 2020, 11 (2).

### 4.7. Imagining Our Future: Comparing Green and Sci-Fi Utopian Themes and Effects on Social Change Motivation

**Julian Fernando** (School of Psychology, Deakin University, Melbourne, School of Psychology, Deakin University, Melbourne Burwood, VIC 3125, Australia)

Utopian thinking is an emerging topic of research in social psychology and related areas. Early work has shown that thinking about one’s ideal society tends to predict greater criticism of one’s existing society and great motivation to change it. Here, I discuss two prominent contemporary utopian visions—a Green utopia and a Sci-Fi utopia—and some experiments designed to assess the motivational capacity of those utopian visions. These studies showed that when asked to think and write about a Green utopia people (1) ascribed to that society greater warmth, positive emotions and other positive characteristics (e.g., peace, democracy), and (2) were more motivated to change their society and perform pro-environmental behaviours. When asked to think and write about a Sci-Fi society, however, no such effects were observed. It was also observed that the motivational capacity of the Green utopia could be partly accounted for by an increased sense of self-efficacy (the feeling that one can make a difference in bringing about that kind of society). These results completement those of other research showing that people see future societies as becoming more competent and technological, but less warm and moral. Thus, people may view the Sci-Fi utopia as a fulfilment of the current societal trajectory (requiring no action), but the Green utopia as requiring a diversion of the current society trajectory. My colleagues and I are continuing our work to understand the motivational function of utopian thinking, in particular, the effects of different kinds of environmentally friendly utopian visions.

### 4.8. The Artist as Rebel: Enhancing Awe, Wonder and Connectivity to People, Places and Planet

**Catherine Sarah Young** (Obama Leader, Asia Pacific and Scientia scholar, Art and Design, University of New South Wales, Sydney, NSW 2052, Australia).

How can art affect connection to the planet? The artist and scholar explores this through the lens of original artworks that have been created and exhibited internationally and recontextualised through the years, specifically through two artistic bodies of work: The Apocalypse Project (2013–present), which explores climate change and our environmental futures; and Wild Science (2018–present), which investigated the relationships between science and society. The Projects include, but are not limited to: The Ephemeral Marvels Perfume Store, an olfactory installation of things we could lose because of climate change; The Sewer Soaperie, soaps created from raw sewage that will further exacerbate urban flooding that is caused by stronger typhoons; Climate Change Couture, potential garments of the future under specific environmental impacts; Experiments in Nature, a video performance series that depicts scientific experiments in the wild to interrogate the role of science in society; and Letters for Science, a participatory project that asks the public to write letters to science denialists to ask them to reconsider their views, etc. Finally, the artist discusses at length her latest piece, The Weighing of the Heart, a sculptural installation series that casts the ashes and remains from the Australian bushfire crisis into human hearts as an invitation to reflect on the human toll that this crisis has wrought.

### 4.9. Parent Use of Smartphones and Tablet Computers and Prenatal Attachment

**Rebecca Hood** (Curtin University, Bentley, WA 6102, Australia).

This Prenatal attachment (the relationship between a parent and their baby during pregnancy) is of great importance, as evidence suggests it leads to secure attachment in early childhood and to better child developmental outcomes in the future. Many potential influences on prenatal attachment have previously been explored. However, one possible influence on prenatal attachment yet to be considered is the use of mobile touch screen devices such as smartphones and tablet computers. The advancement of screen technology in recent years has led to devices having a pervasive impact on expecting parents’ lives during their transition to parenthood.

This study aimed to investigate how the use of mobile touch screen devices influences parents’ thoughts, feelings and behaviours towards their baby during pregnancy. Interviews of 27 expectant parents/parents of newborns found that all used devices for a variety of purposes, and all described secure prenatal attachment. Many parents indicated they had not previously considered the influence of device use on their relationship with their baby. On reflection, parents described both negative and positive influences on their relationship with their baby. For example, some described feeling distracted while using their device and feeling more anxious due to reading information online. Others described feeling closer to their baby due to reading about their baby’s development and playing music for their baby on their device while pregnant.

These findings show how devices can be used by parents to feel more connected to their baby during pregnancy while being aware of potential downsides, which may lead to better parent–child relationships after birth as well as better future child outcomes.

### 4.10. “Desirable Green”: Informing Design Guidelines for Restorative Small Urban Green Spaces

**Hildy Steinacker** (Department of Landscape Architecture, University of Sheffield, Sheffield S10 2TN, UK).

The positive mental health implications of exposure to green space and its potential to reduce stress, i.e., to be restorative, has attracted much interest, but does immersion in a green environment systematically lead to a restorative experience or is the pathway more complex? What is perceived as “desirable green” could be an important factor that either enables or hinders this restorative effect. This research project explores to what degree the restorative experience made possible in green spaces is mediated by perceived attractiveness, or preference.

The approach taken in this thesis is to view these aspects as outcomes of processes of socialization and culture. Although research on restoration does incorporate socio-demographic variables, to what degree these influence the restorative experience of nature has not yet been explored systematically. This is what this thesis aims to do. 

The theoretical framework uses theories from sociologists such as Pierre Bourdieu, Pierre Moscovici, or Henri Lefebvre, incorporating a constructionist approach where both the landscape experience of the viewer and the landscape itself are expressions of societal processes. 

At the *in*VIVO 2020 annual meeting, I presented findings from a questionnaire taken by around 450 participants representative of various sociodemographic groups. The questionnaire examines the green space characteristics and features considered by the participants to be conducive of restoration and stress relief. The questionnaire was open-ended, analysed with thematic coding, and the correlation of the answers with the social categories statistically examined. The results provide precious insights into the degree to which representations, expectations and preferences regarding green spaces vary between respondents from different social categories, and the similarities and differences between these groups. To examine whether the preferences of each group moderate their restorative experience, the emotional responses to experimental park designs will be visually tested in the second stage of the research.

### 4.11. Knowledge Translation in the Response to the COVID-19 and Climate Change Co-Emergencies of Our Time

**Cecilia Sierra-Heredia ^1^, Perla Araiza-Viramontes ^1^, Kerri Klein ^2^, Trevor Hancock ^3^, Mira Ziolo ^4^, Maya Gislason ^1^, Christopher Buse ^5^ and Tim Takaro ^1^** (^1^ Faculty of Health Sciences, Simon Fraser University, Burnaby, BC V5A 1S6, Canada; ^2^ Shift Collaborative, Surrey, BC V4A 2H9, Canada; ^3^ University of Victoria, Victoria, BC V8P 5C2, Canada; ^4^ University of British Columbia, West Mall Vancouver, BC V6T 1Z4, Canada; ^5^ Centre for Environmental Assessment Research, University of British Columbia, 3247 University Way, Kelowna, BC V1V 1V7, Canada).

UN Secretary General Antonio Guterres claimed early on in the pandemic that the social and economic devastation caused by climate change will be greater than that of COVID-19 without appropriate actions from multiple sectors (Fearnow, 2020). As part of the broader set of global ecological changes that constitute the Anthropocene, climate change exacerbates existing health inequalities which are being further compounded by the pandemic. However, climate change has also been described as “the greatest global health opportunity of the 21st century”. It is important that public health professionals and students are involved in the design of mitigation and adaptation strategies in partnership with other organizations and sectors. 

The webinar series “Learning for Planetary Health: Lessons from a Pandemic” is a collection of session recordings and files from the SFU Planetary Health research group and other scholars and practitioners. In eleven sessions, the presenters covered a range of topics regarding the relationship between COVID-19 pandemic, planetary health, climate change and human health. The webinars explored policy options to “bounce forward and not back” into a post-COVID future focusing on actions that transform the way we live and improve health for all as we face the overarching threat of climate change. Knowledge Translation is the process of sharing research findings to multiple stakeholders and practitioners in order to inform policy decisions. In order to broaden the learning of stakeholders, such as non-academic audiences, we have created a toolkit with policy briefs, infographics, and op-eds that expand the reach of knowledge presented by these webinars to two specific audiences, grade 12 students and policy makers. The toolkit is a platform for work produced by researchers and presented in webinars to communicate climate action opportunities and strategies emerging from the COVID-19 pandemic.

### 4.12. A History of the Psychologies of the Environment

**James Dunk** (Research Fellow, Department of History, University of Sydney, Camperdown, NSW 2006, Australia).

This paper will briefly sketch the history of the psychologies of the environment, from environmental psychology to psychologies of survival and conservation psychology. Environmental psychology emerged in the middle of the twentieth century in urban contexts, focusing on the built environment and the various risks that it posed to human health. As the field came to encompass the physical environment, it retained the risk matrix. “Global” psychologies or psychologies of survival appeared from the 1980s, integrating methodology and insights from diverse psychological schools to meet the perceived global crises represented by ecological and other crises at a global scale, while conservation psychology, modelled on conservation biology and conservation medicine, addressed problematic aspects of the human–animal relationship with a view to stemming those still-developing crises. The paper will trace through lines across these approaches, including the constitution and scale of the “environment” which each addresses, and the relationship between the human psyche and this environment which each approach theorises. It will conclude with a discussion of global mental health and planetary mental health in light of this psychological terrain.

### 4.13. Change in Complex Systems: Lessons about Policy and Practice for Healthy, Sustainable Healthcare

**Sarah Walpole** (Royal Victoria Infirmary, Newcastle upon Tyne NE1 4LP, UK).

Healthcare, as other sectors, has a duty to reduce its environmental impacts; and an opportunity to do this as part of a positive transformation, benefiting both those working in the sector and the public. 

Healthcare systems are complex, containing multiple sub-systems and comprising multiple, varied relationships with external actors, environments and systems. Through her research on transition to more sustainable healthcare systems, Charlesworth (2016) highlighted six key elements to facilitate this: clear vision, innovation to redesign systems and processes, staff and patient engagement, effective information management, good performance management and good leadership. 

This presentation considered the goals of healthcare systems and outlines a “whole system” perspective. It draws on the SusQI model to consider resource inputs and social, environmental and financial impacts. It draws on case studies of healthcare systems around the world that are already modelling healthy and sustainable practices, and explores how different healthcare system structures, financing and governance may facilitate or inhibit positive change. 

Good leadership at every level is essential to motivate and facilitate good practice. Both numbers and narratives have a role to play in articulating, modelling and stewarding a sustainable healthcare system. Measurable outcomes are important, but less measurable outcomes may even be more important.

A health system requires both shared goals and responsiveness and respect for diverse views, perspectives and values. Being inclusive of diversity of experiences and perspectives can enhance responsiveness (including to the needs of patients), adaptability, dynamism and resilience of the health system (Murray and Frenk, 2000), all of which are essential during transition.

Suggested reading:

- Charlesworth, K. et al. (2016) “Transformational change in healthcare: An examination of four case studies”, AHR, 40 (2), pp. 163–167;
- Murray, C. and Frenk, J. (2000) “A framework for assessing the performance of health systems”, B WHO, 78 (6), pp. 717–731.

### 4.14. Strategies Caregivers Use to Support Adolescents Who Experience Climate Grief

**Taylor Hirschbergand Chris Lowry** (University of Colorado-Boulder, Boulder, CO 80309, USA).

Climate change poses a great threat to all living species. As people come to understand the urgency and the depth of the multi-layered problems associated with the climate crisis, they may come to experience what some scholars have come to label “climate grief” or “climate distress”. Here, this refers to a deep sense of unease, fear, sadness, and sense of loss associated with the changing climate.

The wide-ranging impacts of climate change on adults’ emotional wellbeing has been an increasing focus of scholarly concern over the past decade. Children, however, remain an understudied population, although some anecdotal reports suggest that they are especially prone to experiencing climate grief. As children grapple with how climate change will affect their lives, it is important to understand how they process climate-related grief and how their caregivers are supporting them to cope with future environmental realities. To fill a gap in the literature, this research explores climate grief through the lens of caregivers as well as their adolescent child. It also examines how caregivers are supporting young people, and what, from the perspective of adolescents, is helping them to cope. This project involves open-ended interviews with 20 caregivers and 20 adolescents. It also draws on data obtained through the 21-item Nature Relatedness Scale (NR), which uses a five-point Likert scale to assess human connections to the environment. This study will test the hypothesis that children and caregivers with higher nature relatedness scores experience more climate anxiety, potentially due to a greater sense of Solastalgia.

Improving understanding of the dimensions of climate grief and strategies to support coping provides an opportunity to inform responsive programming and early interventions that can positively impact life course trajectories for both children and their families.

### 4.15. Designing a Carbon Neutral Health System through Sustainable Quality Improvement

**Kathleen Leedham-Greenand Stefi Barna** (Centre for Sustainable Healthcare, Banbury Road, Oxford OX2 7JQ, UK)

Healthcare, as all human activities, takes place within a social and environmental context. Growing numbers of healthcare professionals express concern about the health implications of environmental challenges facing us, and national health bodies are calling for radical reductions in health sector carbon and waste. Sustainability is one of the domains of quality in healthcare. The sustainable quality improvement movement aims to create a sustainable health service whilst improving patient outcomes. Clinicians involved in sustainable quality improvement projects report greater job satisfaction when they engage work on the “triple bottom line” (financial, environmental, social) to improve patient outcomes, increase staff work satisfaction, and future proof the health service and the communities it supports. Sustainable quality improvement (SusQI) methods include health system changes to promote health, prevent disease, increase patient agency, and develop lean care pathways. This session examines how the perspective of sustainable value can enrich clinical teaching and practice while tackling real-life ethical issues in healthcare delivery. Practical cases are used to illustrate the key principles of sustainable practice and measure the triple bottom line, thus safeguarding the health system for the patients of today and of tomorrow.

### 4.16. Implementation of an Air Quality Forecasting Operating System for Health Surveillance and Sustainability in the Salvador Metropolitan Region, Brazil

**Nelzair Vianna ^1^, Larissa Zanutto ^2^, Fanny Velay-Lasry ^2^, Samya Pinheiro ^2^ and Andre Fraga ^3^** (^1^ Oswaldo Cruz Foundation, Rio de Janeiro 40296-710, Brazil; ^2^ Aria Tecnologies 92100; ^3^ Secretary of Sustainability, Innovation and Resilience, Brazilian government, Rio de Janeiro 40010-010, Brazil).

Air pollution is a threat to public health in the 21st century. In Latin America, more than 100 million people are exposed to air pollution. There is a gap in air quality monitoring in cities around the world. Environmental risk factor monitoring and forecasting systems are strategic for the adoption of preventive measures and health surveillance alerts. The engagement of stakeholders is crucial to develop strategies to control air pollution. Objective: To define air quality and health risk indicators relevant to health surveillance and civil defence, allowing the mapping of areas of higher risk and vulnerability for the population. Methodology: The SOPRAR project was built through an articulated intersectoral mobilization, scientific institutions, private and public sector, The project includes the configuration of emission, chemical, and transportation models to represent the local conditions. The system operates based on CHIMERE model runs (48 h forecast) forced by weather forecast simulations (WRF) performed by the local civil defence, integrating an updated emissions inventory. Model results, pollutants concentration maps and air quality index will be available by WEB service. A tailored health risk indicator will be developed to make the SOPRAR system. The project is committed with the technology and know-how transfer to local actors. Results: The emission inventory was updated based on available local data regarding traffic, road network, industries processes and land use. Total emissions in the Salvador Metropolitan Region corresponds to 3327.03 tons/year of particulate matter, 14,964.32 tons/year of sulphur dioxide, 75,572.28 tons/year of carbon monoxide, 24,756.30 tons/year of nitrogen oxides and 21,756.64 tons/year of volatile organic compounds. Conclusions: The system is as an innovative management tool based on urban environment air quality modelling and will provide support for the activation of air quality protection and communication service protocols for use in research, public policies for primary care and health surveillance.

## 5. Food Systems from the Ground up: Can Food Solutions Also Be Climate Solutions?

**Figure 4:**
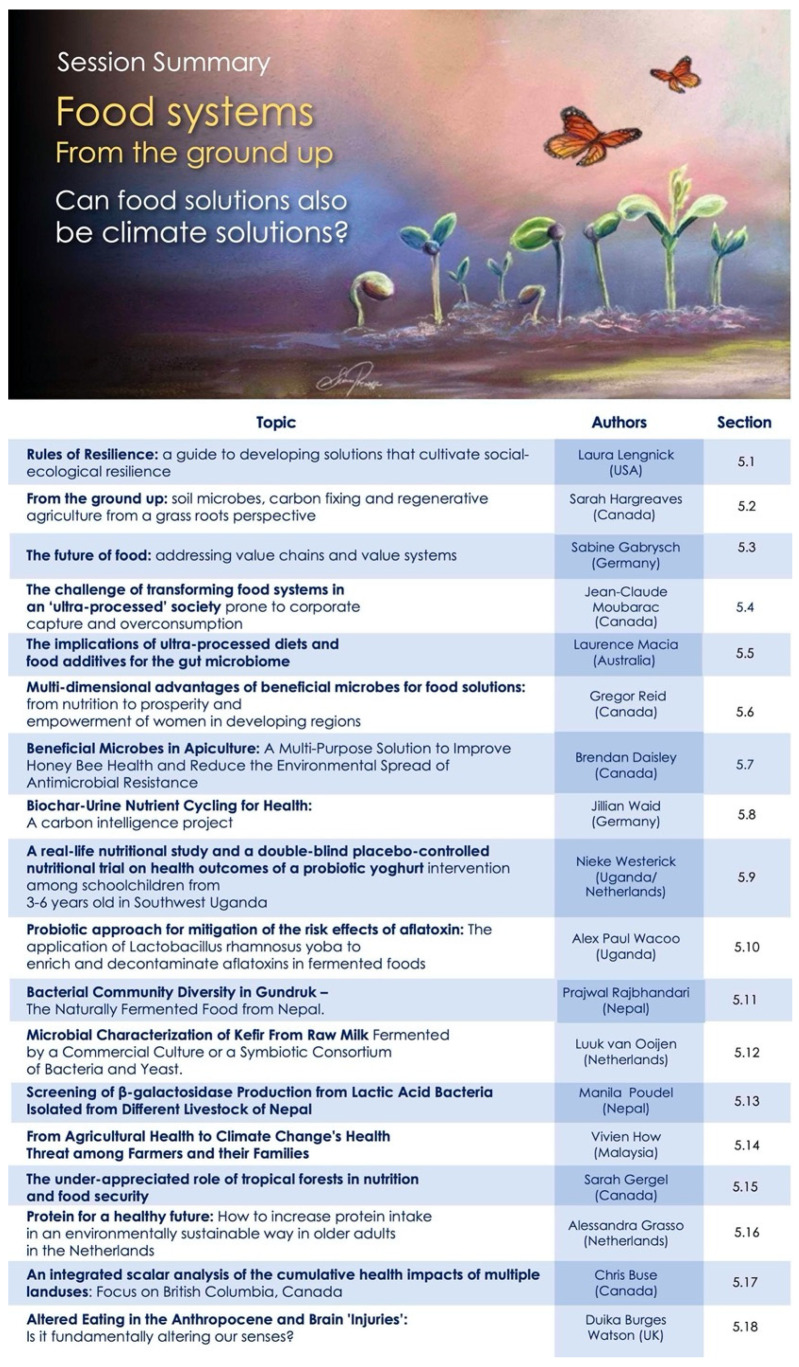
Food systems from the ground up: can food solutions also be climate solutions? Overview of topics and speakers in the session on food systems.

This session explores the complex challenges of global food systems, the adverse impact on natural ecosystems (soil and water contamination, waste and emissions), local communities and growers, and human health. Speakers consider aspects of the modern food environment, spanning from personal ecology to the social, economic and marketing forces that affect both food choices and access to healthy food. We consider the necessary shift to regenerative agro-ecological systems, which restore soils, retain water, increase biodiversity, cycle nutrients to produce more nutrient-rich foods, and fix carbon for climate solutions (see Figure 4).

### 5.1. Why Think Resilience: New Tools for Shaping Change

**Laura Lengnick** (Cultivating Resilience, LLC, Asheville, NC 28805, USA).

There is a growing sense in government, business and civil society worldwide that “business as usual” is no longer an option when it comes to food. It is widely acknowledged that the global industrial food system fuels the wicked challenges of our times—concentration of wealth, loss of biodiversity, energy use, population growth and climate change—that have put us on the path to planetary collapse. The question is not IF, but HOW we change the way we eat. Many different solutions—how do we choose among them? Resilience thinking can help. Social–ecological resilience thinking offers new concepts, a new language and an effective framework for decision making that is uniquely suited to the novel uncertainties of our times. Resilience thinking encourages us to remember that resilience is about much more than simply bouncing back. Research shows that investments that cultivate high response and transformation capacity in the food system and in other critical resource systems are less expensive and more effective ways to sustain community wellbeing over the long term. Resilience thinking identifies solutions that cultivate the social–ecological behaviours associated with high response, recovery and transformation capacity: networks of equitable relationship; regional self-reliance; and the local accumulation of community-based wealth, including natural, human, social, financial, and technological resources. Because these resilient behaviours are well aligned with the core principles of the US sustainable agriculture and food movements, sustainable food advocates have sown the seeds of our resilient food future through more than 40 years of work to develop, for example: cooperative processing, distribution and marketing networks such as *Shepherds Grains* in the Pacific northwest, *Co-op Partners Warehouse* in the Midwest, and *Hickory Nut Gap Meats* in the Southeast; regional food systems assessments such as *A New England Food Vision*; *Maine Harvest*, the first federal credit union in the US to focus investments on regional food system development; and *The Agriculture Resilience Act* which supports investment in regionally led research and development to promote climate-resilient agriculture and food systems.

### 5.2. From the Ground up: Soil Microbes, (Carbon Storage) and Our 3Rs from a Grass + Roots Perspective

**Sarah Hargreaves** (*farmer and educator*, Three Ridges Ecological Farm, Aylmer, ON N5H 2R4 Canada).

We summarize our guiding principles as ecological farmers in “3Rs”: Relationships, Regeneration and Resilience.

A vast network of relationships emerges when we promote a diversity of plants, animals, microbes and people on the farm. Myriad relationships facilitate nutrient cycling and energy flows, soil regeneration, pest and disease management and wildlife and pollinator health.

We use tools such as managed rotational grazing, silvopasture, water retention earthworks, and ecological forestry to design a landscape that captures, sinks and stores water. These tools also help us create a diversity of microclimates to grow a diversity of plants and move a diversity of animals on pasture. This diversity, in turn, stimulates a diversify of microbes to build soil organic matter in life (through decomposition and soil aggregation) and in death (through necromass, or dead microbial cells). Fostering diverse and strong relationships is how we grow nutrient-rich food and regenerate nature’s cycles and, ultimately, bring ecological and economic resilience back to this land. 

### 5.3. The Future of Food: Addressing Value Chains and Value Systems

**Sabine Gabrysch ^1,2,3^** (^1^ Potsdam Institute for Climate Impact Research (PIK), Member of the Leibniz Association, 14412 Potsdam, Germany; ^2^ Charité—Universitätsmedizin Berlin, 10117 Berlin, Germany; ^3^ Heidelberg Institute of Global Health, Heidelberg University, 69120 Heidelberg, Germany).

A “planetary health” perspective is required for transformative food solutions that are more beneficial to human health, other species and the environment. The current food system is failing on multiple levels, and is hugely wasteful, with at least one quarter of food lost or wasted. Poor nutrition is the leading cause of disease globally—two billion people are undernourished or deficient in micronutrients, and two billion are overweight or obese—driving the growing “double burden” of disease. Agriculture also has devastating impacts on natural systems, pushing us beyond several planetary boundaries. This includes (1) land use for monocultures of crops that replace natural ecosystems and their species, contributing to biodiversity loss, (2) fresh water overuse and contamination with nitrogen fertilisers, pesticides and plastic waste, and (3) greenhouse gas emissions, including from deforestation, degrading soils, energy use, methane from livestock and nitrous oxide from fertiliser, generating approximately one quarter of anthropogenic greenhouse gases, thus contributing substantially to climate change.

Our destructive model of progress is fundamentally driven by an obsession with economic growth and a wasteful consumerist ideology, failing to recognise finite systems, ecological interdependence or the value of life on our planet. The food system is geared to benefit multinational companies, including agribusiness and producers of ultra-processed foods and beverages, largely at the expense of consumers, communities, local farmers, and the environment (see Raj Patel’s Stuffed and Starved).

Jared Diamond describes in “Collapse” that survival of societies depends on their capacity for long-term planning, willingness to reconsider core values and beliefs, and wisdom to change trajectory. There is a pressing need to revisit our current values, and to recognise and appreciate our interdependence with the web of life, with our planet. This also means changing policies and practices to prevent predatory behaviour and place greater value on the health of people, communities and environments on all scales.

We are currently faced with a “syndemic” of multiple global challenges with common systemic drivers. Understanding that these are interrelated means we do not need to solve each separately. Indeed, we can learn from nature in terms of multi-solving strategies and shared solutions. For food production, this involves nutrient cycling, multi-functionality, diversity and resilience through regenerative agroecological systems that restore soils, retain water, increase biodiversity and produce nutrient-rich foods, with low greenhouse gas emissions, even carbon negative. Shifting to a healthier and sustainable diet (with more vegetables and less meat and dairy) will release land, reduce emissions and be better for animal and human health. Many such win–win–win strategies are already occurring at the local “niche” level.

Sudden events, such as the pandemic, and megatrends can open windows of opportunities that makes these previously niche-level initiatives more mainstream, to alter the dominant norms, but societal change will not happen by itself, or from above. It will depend on alliances of grassroots change-makers to empower themselves and work together to build vision into action for healthier people on a healthier planet. 

Suggested reading:

Raj Patel. Stuffed and Starved: The Hidden Battle for the World Food System. 2012, Melville House, Brooklyn, USA.

Jared Diamond. Collapse: How Societies Choose to Fail or Succeed. 2004, Viking Press. New York, USA.

Boyd A Swinburn et al. The Global Syndemic of Obesity, Undernutrition, and Climate Change: The Lancet Commission report. The Lancet 2019; 393 (10173): 791–846.

### 5.4. The Challenge of Transforming Food Systems in an “Ultra-Processed” Society Prone to Corporate Capture and Overconsumption

**Jean-Claude Moubarac** (Department of Nutrition, Faculty of Medicine, University of Montréal, Montreal, QC H3T 1J4, Canada).

Transforming food systems such that they become healthy, just and sustainable is a political project that requires a common set of values and beliefs about food, nutrition and health. Food are commons goods and food practices should aim to nourish and protect human and planetary health, while benefiting local communities and livelihoods. However, there exists an inherent tension between public health objectives to protect and promote health, and commercial interests of corporations to produce and promote ultra-processed products that are highly profitable but unhealthy and destructive (known as “commercial determinants of health”). Indeed, these products are formulations of refined substances and additives that are nutritionally poor, linked to severe adverse health outcomes and cause harm to the planet, but they are extremely profitable, and they are designed to favour overconsumption. Food corporations that make ultra-processed products are, however, legally obliged to create growth and therefore cannot be held responsible for the health consequences associated with them. Indeed, in front of the law, corporations are given the status of “moral persons” with the rights and freedom to buy and sell food, regardless of the consequences on society. In this situation, changing food systems will require changing the nature of corporations in order to favour the development of a private sector that is in harmony, not in conflict, with public health goals to promote and protect human and planetary health.

### 5.5. The Implications of Ultra-Processed Diets and Food Additives for the Gut Microbiome

**Laurence Macia** (The Charles Perkins Centre, School of Medical Sciences, Faculty of Medicine and Health, The University of Sydney, Sydney 2006, Australia).

The interaction between gut microbiota and host plays a central role in health. Dysbiosis, detrimental changes in gut microbiota and inflammation have been reported in non-communicable diseases. While diet has a profound impact on gut microbiota composition and function, the role of food additives such as titanium dioxide (TiO_2_), prevalent in processed food, is less established. We investigated the impact of food grade TiO_2_ on gut microbiota of mice when orally administered via drinking water. While TiO_2_ had minimal impact on the composition of the microbiota in the small intestine and colon, we found that TiO_2_ treatment could alter the release of bacterial metabolites in vivo and affect the spatial distribution of commensal bacteria in vitro by promoting biofilm formation. We also found reduced expression of the colonic mucin 2 gene, a key component of the intestinal mucus layer, and increased expression of the beta defensin gene, indicating that TiO_2_ significantly impacts gut homeostasis. These changes were associated with colonic inflammation, as shown by infiltration of CD8^+^ T cells, increased macrophages as well as increased expression of inflammatory cytokines. These findings collectively show that TiO_2_ is not inert, but rather impairs gut homeostasis which may in turn prime the host for disease development.

### 5.6. Multi-Dimensional Advantages of Beneficial Microbes for Food: From Nutrition to Prosperity and Empowerment of Women in Developing Regions

**Gregor Reid** (Lawson Health Research Institute and Western University, London, ON N6A 3K7, Canada).

We are born into a microbial world having no say in the country or living standards we enter into. In time, we learn from what is around us. For some, it is easy to forget that we are equal in terms of species if not the imprint we can make on the planet, but equality is a term with many features. Disease can strike everyone, except if a person is poor, malnourished, homeless, uneducated and without access to good healthcare, the consequences can be very different. While a single 1 g sachet containing a fermentative Streptococcus thermophilus and a probiotic *Lactobacillus rhamnosus* strain cannot address all these issues, it can create a surprisingly important dent. Shown to ferment milk, cereal, fruits and vegetables, the organisms can enhance immunity, counter pathogens, detoxify certain pollutants and produce nutritious good tasting foods. Moreover, one sachet can produce 100 L of yoghurt, form the basis of a profitable microenterprise and value chain, and empower women, men and youth in communities without their challenges to seek. In Uganda, Tanzania and Kenya this initiative reached over 260,000 consumers, helped create hundreds of small businesses and improved school food programs. Now led by Western Heads East and Maimuna Kanyamala in Tanzania and Yoba-for-life in Uganda and other countries, the opportunities to grow locally are continuing. Meanwhile, our lab has expanded the research to use probiotic strains to reduce honeybee colony collapse caused by pathogens and pesticides. As these pollinators are critical to the food chain, efforts are needed to save them. In addition, we are assessing whether probiotic strains can reduce death and failure to thrive in Chinook salmon. This application is challenging because of water temperature and the absence of a permanent gut microbiota. Nevertheless, fish are a critically important staple for many people around the world and while salmon are not found in Lake Victoria, Tilapia and other fish there, as well as off the coast of China, are exposed to an excessive range of pollutants that certain probiotic strains might help to counter. Feeding probiotics to farm fisheries is logistically easier than lakes and oceans, but humans have never been deterred by challenges. If researchers in developed countries can partner with researchers and communities in developing countries, the least it will teach us is that we are one species, and the colour of our blood is the same. We have much to learn from each other—this is not a one-way street. Beneficial microbes can be the tools through which humanity and the ecosystem can grow and learn together. Let us encourage the next generation of talented students to make this area fulfil its potential.

### 5.7. Beneficial Microbes in Apiculture: A Multi-Purpose Solution to Improve Honeybee Health and Reduce the Environmental Spread of Antimicrobial Resistance

**Brendan Daisley, Andrew Pitek, John Chmiel, Shaeley Gibbons, Anna Chernyshova, Kait Al, Kyrillos Faragalla, Jeremy Burton, Graham Thompson and Gregor Reid** (Western University, London, ON N6A 3K7, Canada).

Antibiotic administration in apiculture is intended to prevent bacterial disease but inadvertently contributes to the global dissemination of antimicrobial resistance and can harm honeybee (*Apis mellifera*) symbionts via broad-spectrum activities. For example, we find that routine administration of oxytetracycline increases tetB (an efflux pump-based resistance gene) abundance in the gut microbiota of nurse-age worker bees and at the same time depletes key symbionts known to regulate immune function and nutrient metabolism, such as *Frischella perrera* and *Lactobacillus* Firm-5 strains. Functionally, these microbial changes are associated with a ~50% decrease in capped brood (marker of hive nutritional status and productivity) and a ~30% reduction in antimicrobial capacity of adult hemolymph (indicator of immune competence). To assess how probiotic lactobacilli might be used to counter these negative effects on hives and potentially reduce the need for antibiotics, we fed three probiotic strains of lactobacilli (2 strains exogenous and 1 strain endogenous) to bee colonies using an edible BioPatty. The findings demonstrate that combination therapy with probiotics can: (i) rescue brood count deficits during antibiotic recovery, (ii) mitigate antibiotic-associated microbiota dysbiosis via host-mediated immunoselective regulation of core microbiota members, and (iii) maximize the intended benefit of oxytetracycline by suppressing larval pathogen loads to near-undetectable levels. We conclude that microbial-based therapeutics may offer a simple but effective multi-purpose solution to reduce honeybee disease burden, environmental pollution by xenobiotics, and spread of antimicrobial resistance.

### 5.8. Biochar-Urine Nutrient Cycling for Health: A Carbon Intelligence Project

**Jillian Waid ^1,2,3^** (^1^ Heidelberg Institute of Global Health, Heidelberg University, Heidelberg, Germany; ^2^ Helen Keller International, Bangladesh Country Office; ^3^ Research Department 2, Potsdam Institute for Climate Impact Research (PIK), Member of the Leibniz Association, 14473 Potsdam, Germany).

The Food and Agriculture Approaches to Reducing Malnutrition (FAARM) cluster-randomized trial evaluates the impacts of a homestead food production program in Sylhet Division, Bangladesh. During formative research, low soil fertility was found a production constraint by the majority of farmers. The intervention sought to test and scale the use of a locally produced, low-cost, urine-enriched biochar-based fertilizer to increase yields. This fertilizer combines liquid organic nutrients and biomass transformed into biochar. Urine is a highly efficient fertilizer but underused because of odour. Biochar can be produced from crop waste in soil-pit kilns at the village level. It is a porous material that can soak up urine and transform it into an odourless solid fertilizer. This intervention was tested for acceptability and scaled through two projects (BUNCH 2016–2017, BUNCH2Scale 2017–2020) nested within the FAARM trial (FAARM website: https://bit.ly/2NJud9y accessed on 1 August 2021).

The fertilization system’s performance was measured through four rounds of participatory farmer trials piloted in 2017 and scaled in 2018. The use of the technology was examined through multiple rounds of the FAARM surveillance system, including all registered trial participants (early areas from May to June 2017, all areas from July to October 2018, and sustainability assessment from November 2018 to August 2019).

By July 2018, 94% of farmers in the intervention group had heard of urine–biochar fertilizer, 87% had attended training, and 81% had tried the new fertilizer. Reported benefits included better yields, healthier plants, fewer pests, lower input costs, and better-tasting vegetables. During each growing season following project scale-down, well over one-third of farmers continued to use biochar-based fertilizer. In farmer field trials, where one plot fertilized with usual practice was compared to another plot fertilized with nutrient-enriched biochar, biochar-based fertilizers increased yields from 28% to 68%, depending on the crop type.

Biochar–urine fertilizer was thus found to be an acceptable and effective technology for increasing home garden yields in the FAARM project area in Sylhet, with the potential to scale this technology further within Bangladesh and similar settings. Biochar is a win for yields, soil fertility, and the planet. These fertilizers increase soil organic matter, biological activity, and water-holding capacity, in contrast to commercial mineral fertilizers that allow soluble nutrients to leach into groundwater. While the production of commercial mineral fertilizers is energy intensive and contributes to greenhouse gas emissions, biochar functions as a carbon sink.

Further reading: Sutradhar, I., Jackson-deGraffenried, M., Akter, S. et al. Introducing urine-enriched biochar-based fertilizer for vegetable production: acceptability and results from rural Bangladesh. Environ Dev Sustain (2021). https://doi.org/10.1007/s10668-020-01194-y.

### 5.9. A Real-Life Nutritional Study and a Double-Blind Placebo-Controlled Nutritional Trial on Health Outcomes of a Probiotic Yoghurt Intervention among Schoolchildren from Three to Six Years Old in Southwest Uganda

**Nieke Westerick ^1,2^, Wilbert Sybesma ^1^ and Remco Kort ^1,2,3^** (^1^ Yoba for Life foundation, Amsterdam, The Netherlands; ^2^ ARTIS-Micropia, Amsterdam, The Netherlands; ^3^ Department of Molecular Cell Biology, VU University Amsterdam (VUA), De Boelelaan 1085, 1081 HV Amsterdam, The Netherlands).

Children in Southwestern Uganda have poor growth indicators and a high incidence of common childhood diseases such as respiratory tract infection, skin disease and diarrhoea. In response to these challenges, the consumption of locally produced milk and probiotic yoghurt containing *Lactobacillus rhamnosus* yoba 2012 is currently promoted at pre-primary and primary schools in seven districts in Southwestern Uganda. Parents are encouraged to pay approximately USD 3 per school term of 3 months, for their child to take 125 mL of probiotic yoghurt daily, as produced by one of the local probiotic yoghurt producers. Currently over 20,000 children participate in this program. The health impact of probiotic yoghurt consumption in this program versus milk was assessed in a real-life interrupted time series study. A total of 1116 children from three to six years old (probiotic yoghurt n = 584, milk n = 532) were assessed on daily basis with regards to incidences skin disease and common cold during three weeks of baseline and eight weeks of intervention. The study found a significant reduction over time in the incidence of skin diseases (RR 0.62, CI: 0.43–0.88) and a trend indicating a reduction of the common cold symptoms (RR 0.85, CI: 0.52–1.41) in the intervention group as compared to the control group after eight weeks of intervention. In a second phase of the research, a double-blinded placebo-controlled interrupted time series study was conducted to confirm the results of the first study, in which a total of 195 children from three to six years old (probiotic yoghurt n = 100, placebo dairy product n = 95) participated. The study showed a reduction over time in the incidence of common cold (*p* value = 0.0032) and showed a trend for a reduction in the incidence of skin conditions (*p* value = 0.2950) for the probiotic yoghurt group versus the placebo group. However, at baseline, the incidence of skin conditions as well as common cold were higher in the intervention group, hence despite the significant relative decrease in the incidence of the disease, the final risk ratios (RR) of respectively 1.0 (CI: 0.87–1.2) and 1.0 (CI: 0.71–1.4) did not show a significant effect of the intervention on either condition.

### 5.10. Probiotic Approach for Mitigation of the Risk Effects of Aflatoxin: The Application of Lactobacillus rhamnosus Yoba to Enrich and Decontaminate Aflatoxins in Fermented Foods

**Alex Paul Wacoo ^1,2,3^, Nieke Westerik ^1,2^, Wilbert Sybesma ^1^ and Remco Kort ^1,2,4^** (^1^ Yoba for Life Foundation, Hunzestraat 133-A, 1079 WB Amsterdam; ^2^ Department of Molecular Cell Physiology, VU University Amsterdam, De Boelelaan 1085, 1081 HV Amsterdam; ^3^ Department of Medical Biochemistry, School of Biomedical Sciences, College of Health Sciences, Makerere University, Kampala, Uganda; ^4^ ARTIS-Micropia, Amsterdam, The Netherlands).

Background and rationale: Food safety require urgent attention especially in developing countries where regulation of local food is neglected. In the 2018–2019 financial year, Uganda lost USD 52.4 million due to rejection of maize resulting from high aflatoxin contamination. There was no record of the rejected maize being destroyed, therefore it could have been sold and consumed locally in Uganda. Exposure to aflatoxins is linked to liver cancer contributing to approximately 25,200—155,000 of 550,000 to 600,000 new cancer cases yearly. A total of 83% of these deaths occur in Sub-Saharan Africa and East Asia due to synergistic contribution between aflatoxins and highly endemic hepatitis B infection. Since maize is a major food crop in East Africa, the development of technologies that can reduce aflatoxins is highly relevant. *Lactobacillus rhamnosus* GG, under the generic name *Lb. rhamnosus* yoba 2012, successfully ferments milk to make yogurt, maize porridge (*Uji*), and *Obushera*, but the precise mechanism behind the reduction of aflatoxins during fermentation has not yet been studied. The effect of administered *L. rhamnosus* yoba 2012 in minimizing the dangerous effect of ingested aflatoxin is unknown.

Objective: The objective of our research is to evaluate the prevalence of aflatoxins in maize flour in five major markets and selected households within Kampala, Uganda. The second objective is to assess the effect of the yoba starter culture bacteria on aflatoxins reduction during fermentation of maize porridge and to understand the mechanism of action. The impact of consuming probiotic fermented foods on the reduction of aflatoxin ingested in contaminated food was assessed.

Methodology: 60 maize flour samples from five major markets of Kampala and 72 samples from households were analysed for aflatoxins contamination using a novel immunosensor for point-of-care measurements that has been developed in our laboratory. *Kwete* spiked with 120 µg/Kg of total aflatoxin was produced by fermenting a suspension of maize flour in water at 37 °C for a period of 24 h. The aflatoxin level in fermented *Kwete* was monitored using HPLC-fluorescence measurements.

Results: A total of 31.7% maize flour samples from the markets were contaminated with aflatoxins and 16.7% of the positive samples had concentration higher than 10 µg/Kg allowable level for East Africa. For household samples, 43% of the samples had aflatoxins which were higher than acceptable limits with the highest concentration registered being 233 µg/Kg. During fermentation of contaminated maize, *Lb. rhamnosus* yoba 2012 increased from log 6 to log 8 cfu/g in 24 h at 37 °C. Simultaneously, the aflatoxins levels in the porridge were completely eliminated.

Conclusions: The study demonstrated that fermentation with the *Lb. rhamnosus* yoba 2012 starter culture offers a practical approach to reduce aflatoxins B1, B2, G1 and G2 in maize porridge during a period of 24 h.

### 5.11. Bacterial Community Diversity in Gundruk—The Naturally Fermented Food from Nepal

**Prajwal Rajbhandari ^1^ and Remco Kort ^2^** (^1^ President and co-founder, Research Institute for Bioscience and Biotechnology, Lalitpur 44600, Nepal; ^2^ Department of Molecular Cell Physiology, VU University Amsterdam, De Boelelaan 1085, 1081 HV Amsterdam).

Naturally fermented foods contain complex and diverse microbial communities, which are a source of beneficial bacteria and vitamins such as B [12]. Different countries have their own traditional fermented foods, including Kimchi in Korea, Sauerkraut in Germany, Miso in Japan and Gundruk in Nepal. Gundruk is one of the national dishes of Nepal and made by fermenting green leaves of mustard, cauliflower or radish and used as pickles, soup or as salads. According to UN FAO, approximately 2000 tons of Gundruk are made locally in households annually but its nutritional information and its health benefits are poorly described. Therefore, this study will isolate, identify and characterize naturally occurring bacterial compositions of Gundruk made by local communities in different geographical regions of Nepal. Along with that, the project aims to explore the variation in microbial communities, its dynamics throughout the production process and characterize the metabolites produced during fermentation with potential health benefits.

### 5.12. Microbial Characterization of Kefir from Raw Milk Fermented by a Commercial Culture or a Symbiotic Consortium of Bacteria and Yeast

**Luuk van Ooijen ^1^, Ton Baars ^2^ and Remco Kort ^1,3^** (^1^ Department of Molecular Cell Biology, Vrije Universiteit Amsterdam, 1081 HZ Amsterdam, The Netherlands; ^2^ Department of Immunopharmacology, Utrecht University, 3584 CL Utrecht, The Netherlands; ^3^ ARTIS-Micropia, 1018 CZ Amsterdam, The Netherlands).

The modern western lifestyle resulted in improvement of personal hygiene and thereby reduction of the prevalence of communicable diseases. Food preservation techniques further reduced the incidence of foodborne pathogens. These developments led to reduced environmental exposure of humans to microbes with adverse effects. Reduced microbial exposure, particularly in early life, is associated to an increased risk for inflammatory diseases. The consumption of raw milk has been associated with a reduced incidence of asthma and allergies. These properties could be explained trough immunomodulatory properties of raw milk believed to be caused by specific peptides, proteins and microorganisms. Consumption of raw milk is not without health risks, zoonotic organisms including Salmonella, Campylobacter and Listeria can be present. Preventive measurements in the form of proper milking and zoonosis detection techniques can limit the health risk for consumers. Fermentation of raw milk could be the solution in consuming raw milk with a further reduced risk of exposure to harmful microorganisms. Kefir is a dairy beverage traditionally fermented by a consortium of bacteria and yeasts. The high microbial load of kefir might aid in restoring the dietary intake of microbes. Knowledge is limited regarding the presence of beneficial raw milk microbes in kefir. Therefore, this study will use amplicon 16s and ITS sequencing of several stages of raw milk fermentation in order to determine which bacteria and yeast are present. By using a genomic spike-in the ratio between yeast and bacteria could be quantified. Furthermore, comparisons will be made between the microbial diversity of raw milk kefir and pasteurized milk kefir based on a commercial culture or an in-house SCOBY. The outcome of this research will provide additional insight for strategies to enhance dietary uptake of microorganisms in order to diversify and improve the human microbiome. This can potentially assist in tackling the non-communicable diseases pandemic.

### 5.13. Screening of β-Galactosidase Production from Lactic Acid Bacteria Isolated from Different Livestock of Nepal

**Manila Poudel, Shyam Suwal and Prajwal Rajbhandari** (Research Institute for Bioscience and Biotechnology, Lalitpur 44600, Nepal).

The dairy industry is one of the most dynamic sectors of Nepal where production of cheese and its market is in constant growth. As a starter for the production of dairy products, Gram-positive Lactic acid bacteria (LAB) are generally regarded as safe. In addition, some of the LAB can produce β-galactosidase (β-GAL) that are responsible for the breakdown of lactose into its simpler forms and form galactooligosaccharides (GOS). A large quantity of cheese whey produced as a by-product from the dairy processing consists of lactose (80–85% dry mass) which can be fermented by the β-GAL into GOS. GOS are prebiotic compounds that enhance the performance and function of gut microflora which could potentially improve human health. The main purpose of this study is to isolate industrially useful lactic acid bacteria producing β-GAL enzyme from milk of various livestock found in varying altitude in Nepal. The milk samples were collected from various livestock (cow, buffalo and goat) at different altitudes (Bishnupurkatti—80 m, Sindhuli—1273 m, Khokana—1450 m and Chitlang—1750 m) in Nepal. LAB present in milk were isolated on selective media (MRS agar for *Lactobacillus* spp. and M17 agar for *Lactococcus* spp.). A total of 85 bacteria isolated from samples were identified via morphological and biochemical characterization. Among the isolates, 40 LAB strains were screened for β-GAL production by using X-gal/IPTG assay which showed blue colonies.

### 5.14. From Agricultural Health to Climate Change’s Health Threat among Farmers and Their Families

**Vivien How, Raihanah Chokeli, Nurul Syazani, Yuswir and Zailina Hashim** (Universiti Putra Malaysia, Serdang, 43400 Seri Kembangan, Selangor, Malaysia).

While chemicals have played a key role in agriculture, they also pose potentially significant threats to healthy ecosystems and human health. A five-year study on environmental, occupational and genetic factors on the health of farmers and farm families explores the potential links between agricultural exposures and chronic diseases. Farm workers performing hand labour tasks in pesticide-treated areas have increased exposure to direct spraying, aerial drift, or contact with pesticide residues in the crop and soil via skin and inhalation. These environmental residues have cross-contaminated the farm children and affected their neurodevelopment. The risk of genotoxicity, on the other hand, has been found to cause farm children’s cells to experience early cell damage that leads to uncontrolled cell proliferation during their adulthood. Since pesticides could bioaccumulate in the human body, pesticide residual was found in the urine of children who live near to this pesticide-treated farmland. Our work also found that heavy metals as impurities that soil receives from agricultural practices bioaccumulated in the fish collected from the paddy trench. The dietary health risks from the low-level accumulative consumption of these contaminated fish have found to increase with ages and body mass index among farming villagers. Exposure to extreme heat stress is yet another growing concern for farming communities due to global climate change, particularly in tropical developing countries. Our recent study also found that there is a disparity in the physiological health status among conventional and agroecology farmers, where the former bears the increased physiological health risk burden under extreme climatic effects. For years, our work provides the evidence-based agricultural health study. Today, it supports the action of sustainable healthy agricultural practices that can lead to lasting change for both individual and environment.

### 5.15. The Under-Appreciated Role of Tropical Forests in Nutrition and Food Security

**Sarah Gergel ^1^, Bronwen Powell ^2^, Laura Rasmussen ^3^ and Frédéric Baudron ^4^** (^1^ University of British Columbia, Vancouver, BC V6T 1Z4, Canada; ^2^ Pennsylvania State University, State College, PA 16801, USA; ^3^ University of Copenhagen, 1165 København, Denmark; ^4^ CIMMYT [International Maize and Wheat Improvement Centre], Mount Pleasant, Harare, Zimbabwe).

Malnutrition impacts at least two billion people. Yet, the role of forests as a source of nutrition is underappreciated. Here, we explore the role of landscape diversity (the combined patterns of forests and fields) in supporting dietary diversity by examining linkages between nutritious diets and forests within the rural tropics. To do so, we synthesize and merge long-term and high spatial resolution imagery of landscape change with household diet surveys across a range of sites in Africa and Southeast Asia to uncover associations between primary forests, forest patches, as well as disturbed and edge habitats in bolstering dietary diversity. This approach helps illuminate direct and indirect pathways (agro-ecological, energy, and market pathways) connecting forested landscapes to diet diversity. Improved evaluation of the role of land cover complexity in food security and nutrition can help avoid overly simplistic views of food security and uncover nutritional synergies with forest conservation and restoration.

### 5.16. Protein for a Healthy Future: How to Increase Protein Intake in an Environmentally Sustainable Way in Older Adults in The Netherlands

**Alessandra Grasso ^1^, Margreet R. Olthof ^1^, Corné van Dooren ^2^, Roline Broekema ^3^, Marjolein Visser ^1^ and Ingeborg A. Brouwer** (^1^ Vrije Universiteit Amsterdam, 1081 HV; ^2^ Voedingscentrum, 2594 AC; ^3^ Blonk Consultant, 2805 PJ, Amsterdam).

Protein intake greater than the recommended amount is suggested to improve physical functioning and wellbeing in older adults, yet it is likely to increase the climate footprint of the diet if environmental sustainability is not considered. Therefore, there is a need to delineate ways to increase protein intake in an environmentally friendly manner in older adults. This study aimed to identify dietary changes needed to simultaneously increase protein intake and reduce greenhouse gas emissions (GHGE) in the diet of Dutch community-dwelling older adults. Mathematical diet optimization was used to model high-protein diets with minimized departure from the habitual intake of 1354 Dutch older adults. First, a high-protein diet defined as one providing >1.2 g protein/kg body weight/day was developed isocalorically while maintaining or improving nutritional adequacy of the diet. Second, adherence to the Dutch food-based dietary guidelines (FBDG) was imposed. Third, a stepwise 10% GHGE reduction was applied. Achieving a high-protein diet aligned with the FBDG without considering GHGE resulted in 5% increase in GHGE in men and 9% increase in women. When a stepwise GHGE reduction was additionally applied, total meat stayed constant until a 50–60% GHGE reduction, with a stepwise increase in poultry and pork (mainly for women) and stepwise decrease in beef/lamb and processed meat, and increases in whole grains, nuts, and meat/dairy alternatives and decreases in discretionary products were needed. A high-protein diet aligned with FBDG can be achieved in concert with reductions in GHGE in Dutch older adults by consuming no more than the recommended 500 g meat per week while replacing beef and lamb and processed meat with poultry and pork and increasing intake of diverse plant–protein sources.

### 5.17. An Integrated Scalar Analysis of the Cumulative Health Impacts of Multiple Land Uses: Focus on British Columbia, Canada

**Chris Buse** (University of British Columbia, Kelowna, BC VIV 1V7, Canada).

Natural resource extraction and development activities interact with a host of land uses that can leave lasting consequences for environmental, community and human health values. Novel research methods are required to account for past, present and future “cumulative impacts” of resource extraction and development across broad geographic areas, and the multiple nested ecological scales they comprise. Developing an integrated understanding of the interrelationships between environments, communities and health is therefore increasingly required to develop next generation research and policy action that can improve health equity and sustainability for all in the face of the grand ecological challenges of the 21st century. Leveraging insights from environmental, social and health impact assessment, this presentation utilizes the case study of British Columbia, Canada to introduce a suite of research methods to conceptualize a novel integrated assessment method merging quantitative and qualitative data to inform an understanding of the cumulative environmental, community and health impacts of natural resource development. These include qualitative “data-driven story-telling” approaches with local stakeholders and Indigenous rightsholders comprising over 1000 person hours of community participation, collaborative quantitative indicator development, and a resulting geospatial analysis of environmental, community and health impacts by applying the CalEnviroScreen method—a novel environmental health justice screening tool. By merging statistical representations with narratives that articulate the lived experience of cumulative impacts, this presentation comments on the current state of cumulative impacts assessment, and contemporary policy approaches to natural resource development in relation to the pursuit of health equity. Implications for exploring cumulative impacts across multiple temporal and geographical scales (e.g., watersheds, airsheds, multiple governing jurisdictions) and their implications for driving healthy public policy are discussed. 

### 5.18. Altered Eating in the Anthropocene and Brain “Injuries”: Is It Fundamentally Altering Our Senses?

**Duika Burges Watson ^1^ and Vincent Deary ^2^** (^1^ Newcastle University, Newcastle upon Tyne NE1 7RU, UK; ^2^ Northumbria University, Newcastle upon Tyne NE1 8ST, UK).

Many people live with an altered sense of taste as a result of illness, life course transitions or environmental disruptions. For instance, the 2018 SHEFBIT cohort study of altered eating in traumatic brain injury suggests that 43.5% of people with severe injury and 9.55% with mild have complete smell loss. However, almost no studies in any population, included brain injury, have examined the extent or impact of diminished or altered (as opposed to completely lost) olfaction on taste, eating preferences and pleasure. Part of the reason for this lack of data appears to be a general lack of interest in olfactory function in relation to health, eating and wellbeing. Yet smell largely governs our overall experience of the “flavour” of food and a diminished or altered sense of smell can have major impacts on flavour, food choice and wellbeing. In this presentation we argue that the modern food environment has fundamentally altered our smell and taste through “injuring” the brain structures that subtend these senses. There is a high degree of neuroplasticity of the brain in relation to what tastes “good to eat”. Consequently, researchers have raised concerns that a diet of ultra-processed food products, typically high in salt fat and sugar, is rewiring neural networks in a way that shapes preferences for foods that are neither good for health nor for the environment. In short, a diminished sense of smell may be as much the result of the spread of ultra-processed foods and the rising dominance of transnational food as it is the result of direct physical injury. Drawing on the work of the Altered Eating Research Network with traumatic brain injury and other illnesses, we reflect on the potential for olfactory training and multi-sensory awareness to “rewire”, repair and restore consumers pleasure in foods.

## 6. Building Mutualism through Nature Connectedness: Inspiring Wellbeing, Meaning, Social and Environmental Responsibility

**Figure 5:**
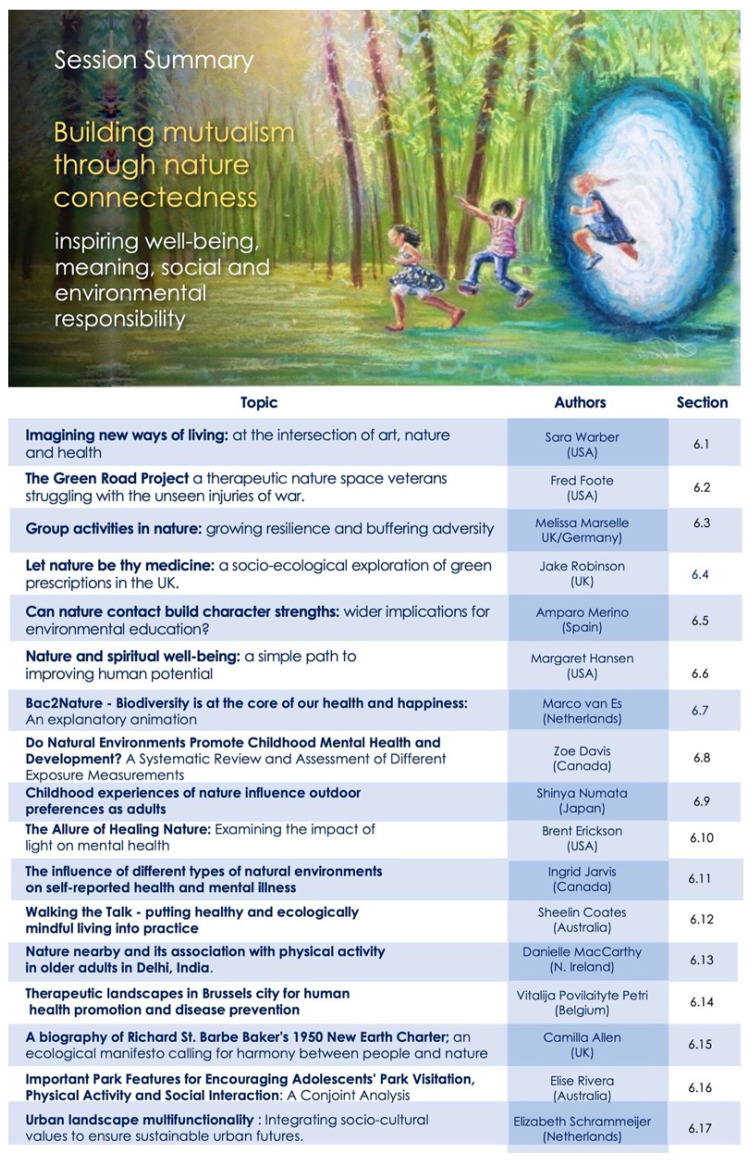
Building mutualism through nature connectedness. Overview of topics and speakers in the session on nature and wellbeing on all scales.

Human connection to nature is known to be a basic psychological need: emerging research shows relationships between nature connectedness as a psychological asset and pro-environmental and pro-social behaviour, as well as human wellness. This session explored the value of nature connection in promoting creativity and mutualistic value systems (as summarised in Figure 5).

### 6.1. Imagining New Ways of Living: At the Intersection of Art, Nature and Health

**Sara Warber** (Clinical Professor Emerita, Family Medicine, University of Michigan, Ann Arbor, MI USA, Scholar, The Institute for Integrative Health, Baltimore, MD 21231, USA).

The health of humans and the health of the planet are interconnected, yet humans perpetuate destruction in denial of this truth. Expressive arts and creativity speak to us in ways that can touch our hearts and motivate future action for planetary health. My overall goal is to inspire others to imagine anew our human ways of living, such that we have a template for transformative action through multiple pathways, all supporting an expanded view of health including both humans and the environment. I present here visual images, predominately by women, as a way to unpack significant human-environment issues and to elicit deep emotion among viewers. The curated images explore what art does for us, ecological grief, art as activism, new inspirations for living, interconnectivity and reciprocal healing, and finding our new map to the future. 

Going forward, my transdisciplinary team and I have identified ways in which art and other image-based forms of communication bring our awareness to the natural world and the predicament of humans in relationship to environmental catastrophe. However, few existing two-dimensional works focus on the known salutogenic aspects of our relationship with nature, nor do they link the impending environmental catastrophe explicitly to its effects on human health. Our plan is to build on this work by soliciting new works that will bring an experience of these reciprocal benefits and perils to ordinary people, encouraging and empowering them towards action as individuals and in communities of varying scales. We invite you to engage with us and follow our progress at www.mutualreawakening.org accessed on 1 August 2021.

### 6.2. The Green Road Project a Therapeutic Nature Space Veterans Struggling with the Unseen Injuries of War

**Fred Footeand Brian Berman** (The Institute for Integrative Health, Baltimore, MD 21231, USA).

Completed in 2018, the Green Road is the Nation’s largest wild-type healing garden. It was designed to provide healing and stress relief to the 12,000 military personnel at Naval Support Activity, Bethesda, MD (NSA/B), home of the Walter Reed National Military Medical Center (WRB), especially for those with traumatic brain injury and post-traumatic stress disorder (TBI/PTSD). The Green Road serves as a source of holistic healing in TBI/PTSD, as a model for the use of green space in urban environments, and as a national laboratory for studying the health effects of nature.

The Green Road consists of a half-mile long ADA-compliant path (along an existing natural stream) and a central woodland healing garden. Along with access from the Walter Reed hospital, the path allows users to avoid traffic and other stressors while moving about the Base. The two-acre central garden sits in an eight-acre woodland ravine, surrounding the stream. The pedestrian path traverses the garden, with branches leading to a variety of forest and stream encounters. The garden site was modified to give enhanced exposure to trees, water, stone, and wild animals (deer, aquatic life, and other fauna). A Communal and a Commemorative Pavilion were also constructed.

Adjacency to the National Institutes of Health and the Uniformed Services University of the Health Sciences (USUHS) has facilitated research into the health benefits of the Project—including a recent comparison of the Green Road with a parallel experience of walking in an urban environment (Ameli R, et al., 2021). The Green Road was unanimously rated as positive (100%). The Green Road yielded mainly positive themes such as enjoyment of nature, relaxation, and feelings of privacy and safety. The Urban Road produced significantly more negative themes. Quantitative assessment showed that a walk on the Green Road significantly decreased distress and increased mindfulness compared to a walk on the Urban Road. Further funded research focused on stress biometrics will be reported in 2021–2022.

This Project is a public/private partnership between the Navy Base and the Institute for Integrative Health (Baltimore) with substantial funding from TKF Foundation (Annapolis, MD), the friends of Shockey Gillet, Capital Funding, LLC, and other donors.

Further reading:

Rezvan Ameli, Perry Skeath, Preetha Abraham et al., 2021. A nature-based health intervention at a military healthcare center: a randomized, controlled, cross-over study. PeerJ 9: e10519.

### 6.3. Group Activities in Nature: Growing Resilience and Buffering Adversity

**Melissa Marselle** (University of Surrey, Environmental Psychology Research Group, Guildford GU2 7XH, UK; De Montfort University, Leicester LE1 9BH, UK).

We currently live in a time with significant stress and risk of poor mental health. Nature-based activities have been used as therapeutic interventions for those experiencing stress and mental ill health. 

This study investigates whether nature-based group walks could be a public health intervention to foster resilience and protect against adversity. An observational research design with propensity score-matched samples compared the mental health of individuals who partook in nature-based group walks once a week or more (frequent nature-based Group Walkers, n = 631) to a comparison group (n = 435). Results showed that nature-based group walks did not buffer the effects of stressful life events on mental health. However, nature-based group walkers had better mental health—both when experiencing stressful life event and not—than the comparison group. This suggests that nature-based group walks at least once per week may be a good treatment to lessen the impact of a stressful life events on mental health. In another study, we investigated whether the type of natural environment for a group walk matters for mental health. Compared to group walks in an urban environment, group walks in the countryside and urban green corridors were associated with significantly less stress and negative emotions—suggesting that contact with these spaces can boost resilience. Our research shows how nature-based group walks could be a public health intervention for mental health and coping with adversity.

### 6.4. Let Nature Be Thy Medicine: A Socio-Ecological Exploration of Green Prescriptions in the UK

**Jake Robinson** (Department of Landscape, The University of Sheffield, Sheffield S10 2TN, UK).

Chronic, non-infectious diseases are on the rise and social isolation is emerging as a major risk factor for mortality. Spending time in natural environments, immersed in the sights, sounds, and smells can bring important health and wellbeing benefits to humans. Furthermore, we are thought to interact with an array of invisible health-regulating biogenic compounds in these natural environments. Indeed, a plethora of studies now support the nature-health nexus and several mechanistic pathways have been proposed. A green prescription is an emerging method of prescribing time spent interacting with nature, e.g., a nature walk, conservation volunteering, or therapeutic horticulture. Green prescriptions are typically provided by general practitioners and social care professionals in conjunction with environment-centric groups. Green prescribing is designed to be a holistic intervention with the potential to help prevent (proactive) and alleviate (reactive) noncommunicable diseases and social isolation. However, little is known about the perceptions and awareness of, and opportunities and constraints (e.g., social, spatial, ecological) associated with green prescribing in the UK. To explore these factors from the perspective of both green prescribing providers and prescribers, we formulated and distributed online questionnaires and collected data from across the UK. A total of N = 261 respondents were included in the analysis. The respondents consisted of general practitioners (n = 118) and nature-based organisations (n = 143). We also collected baseline data to provide an estimation of the spatial distribution of green prescribing activity in the UK. Using geographic information systems (GIS) we will conduct further analyses to explore the socio-spatial and ecological relationships associated with green prescribing, for example, using the Index of Multiple Deprivation and various landscape metrics. At the time of writing this abstract, the analysis for this study is only partially complete. Therefore, we discussed the preliminary findings of this study during the *in*VIVO Planetary Health 2020 annual meeting.

### 6.5. Can Nature Contact Build Character Strengths: Wider Implications for Environmental Education?

**Amparo Merino** (Department of Management, Comillas Pontifical University Calle de Alberto Aguilera, 23, 28015 Madrid, Spain).

The rupture between our self and nature is arguably one central reason behind the current environmental crisis (Liefländer et al., 2013). In fact, a large body of evidence shows that nature relatedness is an antecedent of pro-environmental concern and behaviour (Zelenski et al., 2015). Consequently, fostering interconnectedness with nature has become a central goal of environmental education. Exposing students to natural sites has been common practice in environmental education to nurture this connection among students. However, activities in nature are not always feasible in classroom-based programs nor is it clear that these activities equally accrue nature relatedness among students. Therefore, there are calls for greater understanding of psychological factors that may impinge into greater connectedness. Yet, only limitedly have studies examined the antecedents or correlates of connectedness with nature, which have prompted calls for more research.

Responding to these calls, in a sample of 967 students (Merino et al., 2020), we draw from positive psychology in order to examine whether character strengths (i.e., dispositions to thinking, feeling, and acting towards a moral goal) are antecedents of nature relatedness and which character strengths are more predictive of greater nature relatedness. Our results evidence that intellectual character strengths (i.e., appreciation of beauty, love of learning, and curiosity) are strongly associated with nature relatedness. In addition, they unveil that nurturing the character strength of appreciation of beauty might be the most effective route to increase nature relatedness among learners. These findings allow to suggest that training this strength is a particularly productive route to higher levels of nature relatedness; hence, it merits particular attention in environmental education. Moreover, as beauty is everywhere, it might be trained in a number of contexts, in a diversity of individuals, and through a wide range of resources, approaches and methods, either in nature/outdoor-based or in classroom-based programs. 

The evidence provided about the prominence of appreciation of beauty in nurturing our interdependence with nature supports the idea that developing a sense of wholeness, relatedness and interconnectedness is necessary such that we can deconstruct the frames of reference that lay the foundation of our unsustainable societies (Mochizuki and Fadeeva, 2010). In this sense, it helps to unveil a pathway for environmental (and other disciplines) educators towards the aim of cultivating such sense.

Further reading:

Lieflaänder, A.K., Fröhlich, G., Bogner, F.X., and Schultz, P.W. (2013). Promoting connectedness with nature through environmental education. Environmental Education Research, 19 (3), 370–384.

Merino, A.; Valor, C.; Redondo, R. (2020). Connectedness is in my character: the relationship between nature relatedness and character strengths, Environmental Education Research, 26: 12, 1707–1728.

Mochizuki, Y. and Fadeeva, Z. (2010). Competences for sustainable development and sustainability: Significance and challenges for ESD. International Journal of Sustainability in Higher Education, 11 (4), 391–403.

Zelenski, J.M., Dopko, R.L., and Capaldi, C.A. (2015). Cooperation is in our nature: Nature exposure may promote cooperative and environmentally sustainable behaviour. Journal of Environmental Psychology, 42, 24–31.

### 6.6. Nature and Spiritual Wellbeing: A Simple Path to Improving Human Potential

**Margaret Hansen ^1^and Reo Jones ^2^** (^1^ School of Nursing and Health Professions, University of San Francisco, California, USA; ^2^ School of Nursing, Oregon Health and Science University, Portland, Oregon, USA).

This presentation reviewed Shinrin-yoku (Forest Bathing) as an integrative practice, known to improve humans’ physiologic and psychologic health and wellbeing—by mindfully using the five human senses while relaxing in natural environments. In addition, it may be effective in enhancing or revealing human spirituality. The World Health Organization defines an individual’s wellbeing as an awareness of one’s fullest possible physical, psychologic, social, spiritual, and economic self. Recent evidence suggests that nature promotes spiritual wellbeing. Hence, we performed a scoping review of the literature with regard to the evidence of the interrelationship of Shinrin-yoku/nature and spirituality with an aim to identify gaps in knowledge and assist with furthering empirical research.

The PRISMA extension for Scoping Reviews (PRISMA-ScR) methodological approach was utilized by searching the electronic databases, CINAHL, Google Scholar, Scopus, PubMed, PsychInfo, and ScienceDirect separately, for authors using key terms shinrin-yoku, forest bathing, nature-based therapy, spirituality, health, wellbeing, awe, and wonder.

Of the 30 publications, 13 met the eligibility criteria and were included in the synthesis (see full report in suggested reading below). The findings revealed that, despite the different research methodologies and publications, nature may have a positive effect on human spirituality and, therefore, enriching individuals’ wellbeing.

Thus, Shinrin-yoku is an integrative practice that may enhance or actualize human spirituality. More research is needed to determine the interrelationship of Shinrin-yoku and human spirituality in achieving one’s fullest possible self.

Suggested reading:

Margaret Mary Hansen and Reo Jones. The Interrelationship of Shinrin-Yoku and Spirituality: A Scoping Review. J Alt Comp Med. 2020 Dec; 26 (12): 1093–1104.

### 6.7. Bac2Nature—Biodiversity Is at the Core of Our Health and Happiness: An Explanatory Animation

**Marco van Es ^1^,Erin Kiemeney ^2^andRemco Kort ^3,4^** (^1^ Bac2nature, Amsterdam; ^2^ Creative Business, University of Applied Sciences, Weesperzijde 190, 1097 DZ Amsterdam, The Netherlands; ^3^ ARTIS Micropia, 1018 CZ Amsterdam, The Netherlands; ^4^ Vrije Universiteit, De Boelelaan 1105, 1081 HV Amsterdam, The Netherlands).

Biodiversity is at the core of our health and happiness. Bac2Nature is an explanatory animation about our connection to nature and a call for modern recovery of biodiversity for our health. The animation explains the importance of exposing our immune system to microbial diversity for the benefit of our physical and mental health. It connects the care for our planet to the care for our health. Our immune system is at the core of our health. It has evolved in a highly diverse microbial environment. Therefore, the exposure to microbes is required for a proper development and functioning of the immune system. Since the industrial revolution we have reduced this exposure throughout our lives by decreasing biodiversity of our environment (urbanization), process our food and overuse antimicrobial cleaning agents and antibiotics. The consequence is a malfunctioning immune system leading to an increased risk of several physical and mental chronic diseases. The animation will explain that biodiversity connects our health to the health of our planet and inspires the public with this novel paradigm. It will show the reduction of microbial exposure in our modern society, and sparks the development of innovative solutions, leading to actions to regain our health and happiness.

### 6.8. Do Natural Environments Promote Childhood Mental Health and Development? A Systematic Review and Assessment of Different Exposure Measurements

**Zoe Davis ^1^, Martin Guhn ^1^, Ingrid Jarvis ^1^, Michael Jerrett ^2^, Lorien Nesbitt ^1^, Tim Oberlander ^1^, Hind Sbihi ^1^, Jason Su ^3^ and Matilda van den Bosch ^1^** (^1^ The University of British Columbia, Vancouver campus, V6T 1Z4, Canada; ^2^ University of California, Los Angeles, CA 90095, USA; ^3^ University of California, Berkeley, CA 94720, USA).

Background: Several studies have assessed the relationship between natural environments (NEs) and childhood mental health and development. However, results are inconsistent and association strength unclear, which may reflect the heterogeneity in NE-measurements. We conducted a systematic review to evaluate the relationship between NE-measurements and various indicators of childhood mental health and development, focusing on relative strength of association depending on type of NE-measurement.

Methods: We used a PRISMA protocol to identify eligible studies following pre-defined inclusion criteria. After searching four databases using a number of keywords, 55 articles were included. From these articles, we extracted data on NE-measurement and relative association to childhood mental health and development indicators. Additionally, to evaluate evidence level, a systematic assessment of study quality and risk of bias was conducted.

Findings: The most common NE-measurement was normalized difference vegetation index (NDVI) derived from Landsat (30m resolution). Other measurements included land use/land cover (LULC) datasets, expert audits, and surveys. NE-exposure was typically assigned to participants in various buffer sizes around their residential address, and we found the most consistent association to health outcomes for Euclidian distances of 250 m, 500 m, and within administrative polygons. We found sufficient evidence for an association between NDVI or LULC and improved birth outcomes, general behaviour and social functioning, and decreased ADHD/ADD symptoms. We found limited evidence for an association between LULC and academic achievement and insufficient evidence for an association between NE and mental disorders, memory function, and wellbeing.

Conclusions: This review suggests that several NE-measurements must be evaluated further and compared with each other. It also indicates that research efforts should be coordinated, using consistent NE-measurements to improve evidence level of associations and effect sizes for different health outcomes. Strengthened evidence could contribute to policies that benefit the mental health and development of children by promoting contact with NEs.

### 6.9. Childhood Experiences of Nature Influence Outdoor Preferences as Adults

**Shinya Numata** (Department of Tourism Science Graduate School of Urban Environmental Sciences Tokyo Metropolitan University, Minami-Osawa 1-1, Hachiouji, Tokyo 192-0397, Japan).

Individuals who frequently play in wild environments during childhood have more positive perceptions towards nature, outdoor recreation activities, and potential future occupations in outdoor environments. However, many people may have fewer chances to interact with nature due to the loss of available natural environments and changes in lifestyle, such as increasing indoor activity. Especially, the loss of childhood nature experiences could decrease people’s emotional connection to nature, including nature relatedness or likeability of living things. However, there is surprisingly limited information on how negative emotions towards nature influence people’s contact with nature. In the presentation, I introduced a study: how negative perceptions evoked from outdoor activities influence preferences for later outdoor activities. The results showed that childhood nature experiences decreased levels of disgust sensitivity and fear expectancy later in life. Disgust sensitivity influenced outdoor activity preferences, whereas fear expectancy did not. The SEM results suggest that childhood nature experiences are a strong predictor of perceptions of outdoor activities. Therefore, I concluded that increasing contact with nature during childhood would be useful for decreasing negative perceptions of nature later on.

### 6.10. The Allure of Healing Nature: Examining the Impact of Light on Mental Health

**Brent Erickson** (San Diego State University, San Diego, CA 92182, USA).

This thesis examines historical and contemporary attempts to utilize nature, specifically light, therapeutically. Incorporating research from an array of disciplines, I review and investigate the potential clinical utility of altering light regimens in support of mental health in humans. The mechanics of how light impacts mammalian health are established in a review of animal studies. Paired with epidemiological meta-analyses and experimental human studies evidencing strong correlation between light exposure regimens, health markers, and outcomes, recent evidence indicates a need for both further research and immediate action. One of the most compelling research areas covers the arousing effects of blue light exposure, occurring via human’s non-visual photo-receptive system. Cortisol release and arousing effects from blue light exposure are well documented in animal trials, meanwhile epidemiological meta-analyses show high correlation between the rapid adoption of fluorescent lighting, with the highest intensity in the blue spectrum, and worldwide incidents of depression. This relationship has been observed in experimental studies of human exposure to blue light in the evenings correlating with disturbed circadian rhythms, depression, and anxiety. Epidemiological meta-analyses connect regular sun exposure with positive cardiac health impacts as well as improvements in energy and mood, upending the current clinical doctrine which recommends largely avoiding direct sunlight and replacing Vitamin D with supplements. The extent of artificial indoor lighting and screen use has drastically altered humans’ light exposure. Sunlight’s spectrum changes throughout the day with the blue spectrum most prevalent early in the day shifting towards red in the evening. This appears to synchronize circadian rhythm with the daily cycle of the sun. Ubiquitous artificial lighting and screen use is being labelled a public health crisis. Adapting lighting design to more closely replicate sunlight’s natural rhythms of light spectrum, timing, and intensity may provide tangible, low-cost benefits to mental health.

### 6.11. The Influence of Different Types of Natural Environments on Self-Reported Health and Mental Illness

**Ingrid Jarvis, Sarah Gergel, Mieke Koehoorn and Matilda van den Bosch** (University of British Columbia, Vancouver, BC V6T 1Z4 Canada).

Background: Growing evidence suggests health benefits of natural environments. However, the effects of different types of natural environments (including water) and forms of human-nature contact (access versus exposure) remain relatively unexplored.

Methods: This study uses a cross-sectional design to analyse the relationship between self-reported health and both access and exposure to different types of natural environments in Metro Vancouver, Canada. Data was acquired from the 2013–2014 Canadian Community Health Survey on self-reported general health, mental health, and mood and/or anxiety disorder (weighted n = 1,960,575). Natural environments were estimated using local land use and land designation data and a high-resolution land cover dataset. Access was defined as living within 300 m of a public park (≥1 hectare) and exposure as the percentage of different land cover types within several buffer zones of residential postal codes. Separate logistic regression models were used to estimate associations between access and exposure to natural environments and the respective health outcomes.

Results: Exposure to water within 1000 m of residential postal codes was significantly associated with lower odds of self-reported poor general health, adjusted for confounders (OR = 0.98, 95% CI = 0.96, 0.99). A similar association was found for exposure to shrub and grass-herb vegetation types. Exposure to paved surfaces increased the odds of poor general and mental health, while exposure to buildings was associated with higher odds of mood and/or anxiety disorder. Neither access to public greenspace nor aggregated greenspace exposure were associated with self-reported health. 

Conclusions: Results suggest that type of natural environment should be considered in future research studying the health-promoting aspects of natural environments and that positive health effects appear to be more consistent for daily life exposure than for access to public greenspaces.

### 6.12. Walking the Talk—Putting Healthy and Ecologically Mindful Living into Practice

**Sheelin Coates** (Director, Optimal Ecology, Noosa Heads, QLD 4567, Australia).

In this paper, I outline my efforts to create and model an optimally healthy and sustainable environment at home and in a broader context. I have drawn upon the diverse range of experiences and insights gained from my rural Australian background, my veterinary training and experience in the US intensive animal sector, and subsequent work experience across a variety of industries including the pharmaceutical, construction, financial and natural health sectors, my husband’s prolonged illness and death due to brain cancer, widowhood and single parenthood, and more recently, further post graduate studies in food and sustainability, and the construction of a “healthy” sustainable house, lifestyle and community. In light of my life experience and variety of learnings, I have set out to create a living environment and lifestyle that incorporates as many aspects pertaining to sustainability and health as possible. I have built an optimally healthy, sustainable home in the Noosa UNESCO biosphere, that is adapted to the local environmental conditions and context, is constructed for longevity, utilises minimal toxic inputs, and that is designed to conserve resources (energy, water and other inputs). Food production, water storage and conservation, energy production, thermal regulation, electromagnetic minimisation and a seamless nature interface are all essential elements of the house design. In addition to the built environment, I also adopt many other sustainable and healthy practices with the aim of minimising as many toxic inputs and outputs as possible to create and model an optimally healthy and sustainable lifestyle. A variety of additional aspects have been taken into consideration, including but not limited to: the creation of circular systems, recycling and minimal wastage of resources, enhancement and rehabilitation of the broader ecological habitat, aspects of social sustainability including integration of surrounding community and actively creating and strengthening local and broader community and environmental networks.

### 6.13. Nature Nearby and Its Association with Physical Activity in Older Adults in Delhi, India

**Danielle MacCarthy ^1^,Suresh Rohilla ^2^, Geraint Ellis ^1^ and Deepti Adlakha ^1^** (^1^ David Keir Building, Queen’s University Belfast, University Rd, Belfast BT7 1NN, Northern Ireland; ^2^ Centre for Science and Environment 41, Tughlakabad Institutional Area, New Delhi 110062, India).

In the context of the rapid urbanisation taking place in many developing cities around the world, the impact on both people and nature can be profound. It has become urgent to understand the simultaneous benefit of both a greater ecological presence in urban environments and its relationship for people and health. With rising NCD’s and growing ageing societies, environments play a key role in facilitating health for this population, and in particular countering the effects of sedentary lifestyles. Less well understood are the psychosocial mechanisms which underpin physical activity and its relationship to attachment to place and nature connectedness, and in particular how these operate within the developing world context.

Based on surveys, (n = 228) we assessed people’s rates of nature dose, time spent in nature nearby—parks within four different neighbourhoods, varying in terms of socio-economic status, i.e., high and low levels of greenness, high or low vegetation, rates of physical activity as a whole, as well as measuring levels of place attachment and nature connectedness for each respondent. Preliminary results confirm that a relationship between greener neighbourhoods and physical activity may be witnessed and that nature–dose response may be mediated by nature connectedness. At the time of writing this abstract however, the analysis for this study is only partially complete. I propose to discuss the full findings of this study during the *in*VIVO Planetary Health 2020 annual meeting.

These findings could contribute to our understanding for the future planning of cities and ageing in place discourse, with ramifications for health but also health budgets and wider ecological benefits. The importance of green cities is increasingly recognised for their role in preventative health as well as the wide-reaching benefits for cities with regards to climate adaptation and the range of ecological services greenspaces provide.

### 6.14. Therapeutic Landscapes in Brussels City for Human Health Promotion and Disease Prevention

**Vitalija Povilaityte ^1^, Pierre Duez ^2^ and Katriina Kilpi ^3^** (^1^ Faculty of Medicine and Pharmacy, University of Mons, 25 Chemin du Champ de Mars, B-7000 Mons, Belgium; ^2^ Faculty of Medicine and Pharmacy, University of Mons, 25 Chemin du Champ de Mars, B-7000 Mons, Belgium; ^3^ Swedish University of Agricultural Sciences, SLU, Slottsvägen 5, SE-230 53 Alnarp, Sweden).

An increasing problem in cities is loneliness that directly influences people’s quality of life and susceptibility for unhealthy lifestyles and resulting sickness. According to Dr. Cindy McPherson Frantz, the need to belong is one of the most powerful motivators of human behaviour. It has been suggested that a meaningful psychological relationship with the natural world, i.e., nature connectedness can fulfil our need of belongings and can play a very important role in building human resilience, which results in connection with oneself, nature and the larger community. Places that enable enjoying and meaningfully interacting with the natural world can offer such opportunities to connect with nature. In our study, we assessed the potential of natural environments (e.g., urban gardens, parks and forest) to become a therapeutic landscape in Brussels city and their possible positive health outcomes for human health promotion and disease prevention. Our study showed that constructing a therapeutic landscape becomes possible when using nature-based interventions and nature therapies as efficient practices associated with human health and healing. The evidence of the efficacy of green exercise, garden therapy, eco-therapy and forest therapy are starting to build up, though further studies are required. Nature based solutions for health and wellbeing can produce supportive nature and social spaces that can function as restorative places for human health. Significant impacts of connectedness with nature, one-self and social interactions transform the cultural and social urban fabric into a therapeutic landscape with numerous benefits for human health. Aesthetic qualities and social networks offering a sense of security and inclusion, play a key role in every day green care spaces through the transformative power of making urban nature spots into therapeutic landscapes.

### 6.15. A Biography of Richard St. Barbe Baker’s 1950 New Earth Charter; An Ecological Manifesto Calling for Harmony between People and Nature

**Camilla Allen** (Department of Landscape Architecture, The University of Sheffield, Sheffield S10 2TN, UK).

In 1950, the Men of the Trees, a society formed by the British forester and environmentalist Richard St. Barbe Baker (1889–1982), published a document called the New Earth Charter which set out the foundations of the society’s ecological philosophy. The society had been founded in Kenya as the Watu Wa Miti (People of the Trees) in 1922 when Baker was working as a colonial forester and which aimed to foster a culture of tree-planting to counter deforestation. In 1924 Baker founded a Men of the Trees group in Britain and furthering the aims of the society—to plant and protect trees across the world—became his life’s work. He was a prolific self-publicist and unofficial diplomat, whilst publishing numerous books about his exploits and philosophy, including three autobiographies.

The New Earth Charter declares, “We believe in the development of a fuller understanding of the true relationship between all forms of life in an endeavour to maintain a natural balance between minerals, vegetation, animals and mankind”. This plea for knowledge, peace, and reconciliation preceded many of the twentieth century’s environmental proclamations and publications and followed the 1945 Charter of the United Nations. Within this context, the New Earth Charter represents an important distillation of his ethos, and his desire to mediate between people and nature. Instead of Baker’s biography taking precedence, the paper presents two biographies: that of Baker and—within and beyond that—of the New Earth Charter. It questions the intention and efficacy of the charter, as well as its later reinterpretation as the Earth Charter as important artefacts within the story of human nature relationships.

### 6.16. Important Park Features for Encouraging Adolescents’ Park Visitation, Physical Activity and Social Interaction: A Conjoint Analysis

**Elise Rivera ^1^, Jenny Veitch ^2^, Anna Timperio ^1^, Venurs Loh ^1^ and Benedicte Deforche ^3^** (^1^ Institute for Physical Activity and Nutrition at Deakin University, 221 Burwood Highway, Burwood, VIC 3125, Australia; ^2^ Institute for Physical Activity and Nutrition at Deakin University, 221 Burwood Highway, Burwood, VIC 3125, Australia; ^3^ Department of Public Health and Primary Care, Faculty of Medicine and Health Sciences, Ghent University, Corneel Heymanslaan 10, 9000 Ghent, Belgium).

Parks are key health-supportive settings that offer opportunities to be active, socially interact and connect with nature. Despite their benefits, park visitation and physical activity levels during park visits are low among adolescents—a highly inactive sub-population. Little is known about what park features may encourage adolescents to visit parks and be active and social whilst visiting. This study examined the relative importance of park features for encouraging adolescents’ visitation, physical activity and social interaction in parks.

Adolescents (13–18 years) were recruited in 2019–2020 from five secondary schools located in socio-economically diverse areas of Melbourne, Australia. As part of an in-class activity, participants completed an online survey, which included adaptive choice-based conjoint (ACBC) tasks. This methodology enabled the relative importance of park features for encouraging park visitation and active and social park use to be determined. Included park features (n = 13) for each ACBC task were based on findings from a previous study with adolescents. For each feature, part-worth utility and relative importance scores were estimated with Hierarchical Bayes analyses using Sawtooth Software. 

Participants (n = 244) had a mean age of 14.7 years (SD = 1.3), and 54% were male. The three most important features for park visitation were large swings, large grassy open space and a café; for physical activity, were sports courts, large grassy open space and outdoor fitness equipment; and for social interaction, were a café, barbecue/picnic areas and sports courts. Gender differences were observed; sports features were more important for males, while slides and swings were more important for females across the outcomes.

These results can inform stakeholders regarding which park features to prioritise for optimal park development and refurbishment that maximises visitation and active and social park use among adolescents. Findings highlight the importance of considering specific needs of adolescents when planning park (re)design.

### 6.17. Urban Landscape Multifunctionality: Integrating Socio-Cultural Values to Ensure Sustainable Urban Futures

**Elizabeth Schrammeijerand Peter Verburg** (Vrije Universiteit Amsterdam, 1081 HZ Amsterdam, The Netherlands).

Green spaces are essential for sustainable cities. They provide social-cultural as well as biophysical functions, such as importance for biodiversity, habitat provision and recreation, and are considered important for human wellbeing. Densification of urban areas are thought to provide more efficient infrastructure, reducing traffic and related pollution. In reality, more compact urban forms have a negative impact on the quality of life. Amongst other impacts, green, public, open spaces are lost, while demand for their important functions increase due to an increased number of residents.

Ensuring sufficient and good quality urban green space faces a number of challenges in compact cities and urban development areas. Improved knowledge of green space qualities and how they fulfil the needs of residents is essential for socially sustainable development. However, trade-off analysis that integrates different forms of values and benefits (such as biophysical, social and ecological) and appropriately considers relevant cultural ecosystem services continues to be a major challenge.

Public participatory GIS (PPGIS) is an accepted way to identify citizens’ values and has been used at a city level to inform city-wide planning decisions regarding densification. Building on these studies, we zoom in on a specific case in Amsterdam where densification is being planned. We present a framework whereby insights obtained with PPGIS can be combined with ecological and physical spatial attributes of the landscape in an integrated analysis of urban multifunctionality. Using PPGIS we define the needs of current residents and workers and identify their perception of and preferences for existing green spaces. With spatial analysis we map the demand and supply of social and ecological functions and the benefits provided by the current landscape. We then analyse the mismatches between social expectations and ecological functions in different development scenarios and assess how different green space qualities could be utilised to alleviate trade-offs.

## 7. Targeting Ecological Foundations: Rewilding Environmental Microbiomes: Implications for Human Health and Microbial Ecology

**Figure 6:**
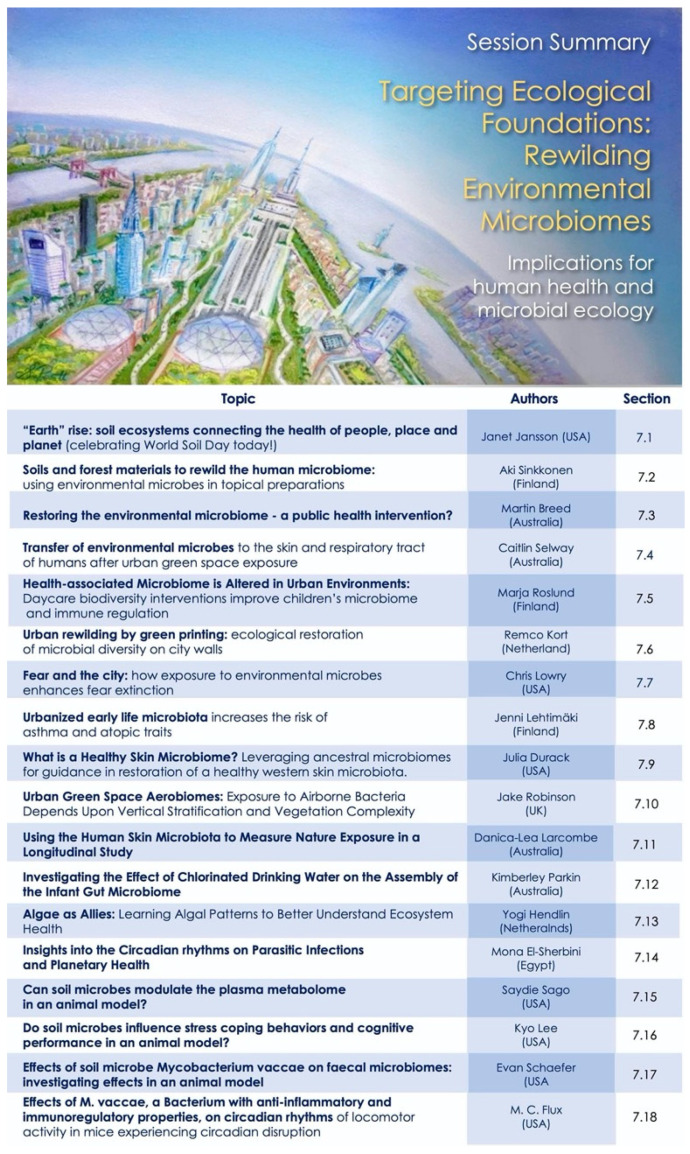
Targeting Ecological Foundations: Rewilding Environmental Microbiomes. Overview of topics and speakers in the session on microbial ecology for health at all scales.

In recent decades, we have begun to see that microbes are the foundation of all life, not only in evolutionary terms, but in every modern ecosystem. Virtually every aspect of human health also depends on microbes for healthy functioning. The health of “our” microbes depends on the health of the wider ecosystems in which we reside. With this knowledge, we can work with nature to restore ecosystems from “the bottom up”. Moreover, microbial symbiosis exemplifies collaboration in nature. A simple unifying principle that is much needed across every domain of human society today. This session explored creative ways in which understanding and harnessing microbial ecology can provide benefits for healthier systems on all scales (Figure 6).

### 7.1. “Earth” Rise: Soil Ecosystems Connecting the Health of People, Place and Planet (Celebrating World Soil Day)

**Janet Jansson** (Pacific Northwest National Laboratory, Biological Sciences Division, Richland, Washington, DC 99352, USA).

Soil is one of Earth’s most precious resources. It supports the growth of plants and the animals and humans that feed on them. Soil is also a living resource with a huge biodiversity of microorganisms. A single teaspoon of soil contains billions of microorganisms. These microbes are largely responsible for cycling of essential nutrients that are needed for growth of plants and animals. In addition, soil microbes are the funnel through which soil organic carbon is cycled. Depending on the soil properties and soil microbial activity, soil organic carbon is either retained in the soil, which is beneficial for soil fertility, or lost as CO_2_ to the atmosphere, which is deleterious for climate warming.

Our research focuses on the impact of climate change on functions carried out by soil microorganisms that are essential for ecosystem functioning. This research spans different ecosystem types, from permafrost in the Arctic to native prairie soils. As the climate warms permafrost is starting to thaw. Permafrost is a huge reservoir of terrestrial carbon and contains as much carbon as the Earth’s atmosphere and vegetation combined. As the permafrost is thawed this enormous carbon freezer starts to warm and microbial activity increases as a result. Microbes can survive in frozen permafrost but have low activity and thus the soil organic carbon turns over very slowly. However, in thawed permafrost active microorganisms start to metabolize the organic carbon, churning out CO_2_ and methane. This leads to aggravation of the greenhouse gas effect and results in increased warming.

The other ecosystem that we study are prairie grasslands. As the climate changes there are shifts in temperature and precipitation patterns that result in either increased precipitation in northeast grasslands or decreased precipitation and drought in southwest grasslands. The resulting shifts in soil moisture have profound impacts on soil microorganisms and their ability to cycle carbon and other nutrients.

By using a “multi-omics” approach, we were able to assess not only how changes in soil moisture impact the microbial community composition, but also their functional genes and expression. For example, we found that genes and pathways for production of stress protecting compounds “osmolytes” were enriched in dry soil when compared to wet soil. These results indicate that soil microorganisms respond in specific ways to changes in their environment. Whether these shifts will be permanent, or not, depend on the resilience of the soil microbial community to short and long-term changes in their environment.

This knowledge is important for being able to model and predict how future climate change will influence soil microorganisms and their ability to support plant and animal life.

### 7.2. Soils and Forest Materials to Rewild the Human Microbiome: Using Environmental Microbes in Topical Preparations

**Aki Sinkkonen** (Head of Adele consortium *, Natural Resources Institute Finland, Itäinen Pitkäkatu 4, 20520 Turku, Finland). * Adele research group members: Cerrone D, Grönroos M, Mäkelä I, Nurminen N, Oikarinen S, Puhakka R, Roslund MI, Saarenpää M, Soininen L, Sun Y, Laitinen OH, Rajaniemi J, Hyöty H, Sinkkonen A.

Before World War Two, green infrastructure and dirt were linked to serious illnesses and child mortality. When serious pathogens and pests were largely eliminated in westernized societies, immune-mediated non-communicable diseases became common. Nowadays, evidence suggests poor contacts with environmental microbial communities are a reason for the high incidence of immune-mediated diseases (Haahtela et al., 2021).

In urban environments, typical playground sand contains magnitudes less microbes than natural forest soils do. We recently realized how humans carry less microbes indoors in urban compared to rural environment. We also found an association between diverse yard vegetation and gut microbial diversity (Vari et al., 2021). Finally, we performed a biodiversity intervention among daycare children (Roslund et al., 2020). In the intervention, diverse vegetation and environmental bacterial communities were associated with enhanced immune modulation (Roslund et al., 2020).

Based on these associations and findings, we designed a diverse microbial inoculant that was manufactured from commercially available, microbiologically diverse gardening soil and plant materials. We tested that the inoculant diversifies skin and gut microbiota (Grönroos et al., 2019), and that its use is associated with immune response (Nurminen et al., 2018). Today, we are performing a randomized double-blind biodiversity intervention study among infants. In the trial, 2 m old infants receive either placebo or lotion and linen containing high microbial biodiversity. Children participate the intervention until 12 m age, and they are followed for two additional years. Thus far, the results show that the trial is safe. The vision is that those who have no access or who are not interested in exposing themselves to rich environmental microbial communities, will eventually have a change to receive the benefits of diverse environmental microbiota indoors.

Suggested reading:

Grönroos M, Parajuli A, Laitinen OH, Roslund M, Vari H, Hyöty H, Puhakka R, Sinkkonen A. 2019. Short-term direct contact with soil and plant materials leads to an immediate increase in diversity of skin microbiota. MicrobiologyOpen 8: e645. doi:10.1002/mbo3.645

Haahtela T, Alenius H, Lehtimäki J, Sinkkonen A, Fyhrquist N, Hyöty H, Ruokolainen L, Mäkelä M. 2021. Immunological resilience and biodiversity for prevention. Authorea preprint. doi:10.22541/au.161670861.12608109/v1

Nurminen N, Lin J, Grönroos M, Puhakka R, Kramna L, Vari HK, Viskari H, Oikarinen S, Roslund M, Parajuli A, Cinek O, Laitinen OH, Hyöty H, Sinkkonen A. 2018. Nature-derived microbiota exposure as a novel immunomodulatory approach. Future Microbiology, 13 (7): 737–744. doi:10.2217/fmb-2017-0286

Roslund M, Puhakka R, Grönroos M, Nurminen N, Oikarinen S, Gazali AM, Cinek O, Kramná L, Siter N, Vari HJ, Soininen L, Parajuli A, Rajaniemi J, Kinnunen T, Laitinen OH, Hyöty H, Sinkkonen A. 2020. Biodiversity intervention enhances immune regulation and health-associated commensal microbiota among daycare children. Science Advances 6 (42): eaba2578. DOI: 10.1126/sciadv.aba2578

Vari HK, Roslund MI, Oikarinen S, Nurminen N, Puhakka R, Parajuli A, Grönroos M, Siter N, Laitinen OH, Hyöty H, Rajaniemi J, Anna-Lea Rantalainen A-L, Sinkkonen A. 2021. Associations between land use types, gaseous PAH levels in ambient air and endocrine signaling predicted from gut bacterial metagenome among the elderly. Chemosphere 265 (February 2021): 128965. DOI: 10.1016/j.chemosphere.2020.128965.

### 7.3. Restoring the Environmental Microbiome—A Public Health Intervention?

**Martin Breed** (Flinders University, Bedford Park, SA 5042, Australia).

It is widely accepted that ecosystem restoration—the repaid of degraded ecosystems—offers multiple values across the peoples-places-planet continuum. Accordingly, the role of environmental microbiota in ecological restoration, particularly in the context of urban ecosystems, has received international attention. Diverse and functional microbial communities are essential for soil formation, nutrient cycling and decomposition, symbiotic relationships between flora and fauna, and are highly biodiverse in their own right. Given that environmental microbiota are also essential to human health, it raises a series of important questions. How does human exposure to environmental microbiota change with ecosystem restoration? What types of restoration interventions promote the restoration of health-associated environmental microbiota? Can ecological restoration—specially restoration that targets the environmental microbiota—have positive effects on human health via environmental microbiota?

### 7.4. Transfer of Environmental Microbes to the Skin and Respiratory Tract of Humans after Urban Green Space Exposure

**Caitlin Selway ^1^, Jacob Mills ^1^, Philip Weinstein ^1^, Chris Skelly ^2^, Sudesh Yadav ^3^, Andrew Lowe ^1^, Martin Breed ^4^ and Laura Weyrich ^5^** (^1^ University of Adelaide, Adelaide, SA 5005, Australia; ^2^ Public Health Dorset, Prince’s St, Dorchester DT1 1TP, UK; ^3^ Jawaharlal Nehru University, Delhi 110067, India; ^4^ Flinders University, Bedford Park, SA 5042, Australia; ^5^ The Pennsylvania State University, State College, PA 16802, USA).

In industrialized countries, non-communicable diseases have been increasing in prevalence since the middle of the 20th century. While the causal mechanisms remain poorly understood, increased population density, pollution, sedentary behaviour, smoking, changes in diet, and limited outdoor exposure have all been proposed as significant contributors. Several hypotheses (e.g., Hygiene, Old Friends, and Biodiversity Hypotheses) also suggest that limited environmental microbial exposures may underpin part of this rise in non-communicable diseases.

In response, the Microbiome Rewilding Hypothesis proposes that adequate environmental microbial exposures could be achieved by restoring urban green spaces and could potentially decrease the prevalence of non-communicable diseases. However, the microbial interactions between humans and their surrounding environment and the passaging of microbes between both entities remains poorly understood, especially within an urban context.

Here, we survey human skin (n = 90 swabs) and nasal (n = 90) microbiota of three subjects that were exposed to air (n = 15), soil (n = 15), and leaves (n = 15) from different urban green space environments in three different cities across different continents (Adelaide, Australia; Bournemouth, United Kingdom; New Delhi, India). Using 16S ribosomal RNA metabarcoding, we examined baseline controls (pre-exposure) of both skin (n = 16) and nasal (n = 16) swabs and tracked microbiota transfer from the environment to the human body after exposure events. Microbial richness and phylogenetic diversity increased after urban green space exposure in skin and nasal samples collected in two of the three locations. The microbial composition of skin samples also became more similar to soil microbiota after exposure, while nasal samples became more similar to air samples. Nasal samples were more variable between sites and individuals than skin samples.

Our study improves our understanding of human-environmental microbial interactions and suggests that increased exposure to diverse outdoor environments may increase the microbial diversity, which could lead to positive health outcomes for non-communicable diseases.

### 7.5. Health-Associated Microbiome Is Altered in Urban Environments: Daycare Biodiversity Interventions Improve Children’s Microbiome and Immune Regulation

**Marja Roslund ^1^, Anirudra Parajuli ^2^, Riikka Puhakka ^1^, Anna-Lea Rantalainen ^1^, Mira Grönroos ^1^, Laura Soininen ^1^, Sami Oikarinen ^3^, Noora Nurminen ^3^, Olli H. Laitinen ^3^, Heikki Hyöty ^3^ and Aki Sinkkonen ^4^** (^1^ University of Helsinki, FI-15140 Helsinki, Finland; ^2^ Karolinska Institutet, SE-17177 Solna, Sweden; ^3^ Tampere University, FI-33014 Tampere, Finland; ^4^ Natural Resources Institute, FI-20520 Helsinki, Finland).

Today, the vast majority of people are living in urban environments where the environmental microbiota is different from that found in biodiverse nature. In urban areas, there is a lack of natural green spaces with diverse microbiota. At the same time, the pollution levels in urban areas alter microbial communities. We asked what happens to the human commensal microbiota when living under pollution stress and how does this affect our health? We have studied how biodiversity and polycyclic aromatic hydrocarbons (PAH) from traffic emissions and domestic wood burning alter environmental and human commensal microbiota. Importantly, we have estimated how these alterations affect immune response and endocrine signalling pathways. First, we studied PAH induced microbiota shifts in urban environments, and estimated associations between PAHs, microbiota and endocrine signalling. Secondly, we compared environmental and commensal microbial communities in typical urban versus biodiverse environments. The study participants included elderly people living in urban and rural environments, and small children living in urban environment.

Pollution studies showed that PAHs levels are associated with both environmental and commensal microbiota. Microbiota alterations in the gut were associated with endocrine signalling, which can affect hormonally mediated processes. Since commensal microbes are able to affect PAH metabolism and detoxification, future research should find out if certain microbial taxa can improve human health by reducing the negative health outcomes induced by PAHs. The results support the altered environmental microbiome hypothesis; both biodiversity loss and pollution contribute to altered microbiomes in the environment and in the human body.

### 7.6. Urban Rewilding by Green Printing: Ecological Restoration of Microbial Diversity on City Walls

**Remco Kort ^1^,Joris Laarman ^1^, Tim Geurtjens ^1^, Olivier de Gruijter ^1^, Jasper Buikx ^2^ and Remco Kort ^2,3^** (^1^ Joris Laarman Studio, Ottho Heldringstraat 3, 1066 AZ Amsterdam; ^2^ Artis-Micropia, Plantage Kerklaan 38-40, 1018 CZ Amsterdam; ^3^ Systems Biology Lab, Vrije Universiteit, De Boelelaan 1108, 1081 HZ Amsterdam).

According to recent estimates, 68% of the global population will live in cities in 2050. An important prerequisite for liveable and sustainable cities is the presence of green infrastructure. This assists in mitigating effects of climate change, the “urban heat island” effect in cities and has a profound effect on the aesthetics of a city. In addition, there is a clear health benefit, as green infrastructures are associated with and enhance exposure to microbes, which is important for the development of our immune system and contributes to our physical and mental wellbeing. The current epidemic of inflammatory diseases in modern urban environments is partly resulting from diminished exposure to microbes, which educate our immune system leading to the suppression of inappropriate inflammation responses. Such diseases include allergies, asthma, and neurodevelopmental disorders, including anxiety and depression. Here we propose to contribute to the ecological restoration of microbial diversity by transforming grey concrete city facades into living green walls. In order to realize our ambition, we plan to develop a robot, which is able to reach the entire surface of the wall by vertical movements. The robot is able to apply water, substrate, algae, fungi and bacteria on the wall, allowing the creation of a living, colourful and dynamic piece of microbial art. The purpose of our project is twofold. On one hand we aim to contribute to an improved living environment in the city, on the other we would like to create awareness for the importance of a diverse microbial environment for our own health, which may contribute to a change in mindset leading to restoration of natural ecosystems.

### 7.7. Fear and the City: How Exposure to Environmental Microbes Enhances Fear Extinction

**Christopher A. Lowry** (University of Colorado Boulder, Boulder, CO 80309, USA).

Anxiety disorders, affective disorders, and trauma and stressor-related disorders, such as posttraumatic stress disorder (PTSD) represent a significant social and economic burden to individuals, their families, and society. Chronic low-grade inflammation has been identified as a risk factor for these stress-related psychiatric disorders and, indeed, machine learning approaches have identified biological signatures of inflammation as among the highest-ranking features predicting subsequent development of a diagnosis of PTSD (Schultebraucks et al., 2020).

The “Old Friends” hypothesis posits that a lack of exposure to diverse microbial environments in modern Western societies is resulting in impaired immunoregulation and, consequently, inappropriate inflammation. The “Old Friends” hypothesis may be relevant to risk of developing stress-related psychiatric disorders. We have shown that individuals raised in an urban environment without daily exposure to pets respond to the Trier Social Stress Test (TSST) with an exaggerated stress-induced inflammatory response, relative to individuals raised on a farm with daily contact with farm animals (Böbel et al., 2018). 

Thus, exposure to environmental microorganisms with anti-inflammatory and immunoregulatory properties has promise for the prevention and treatment of inflammatory conditions, such as allergic asthma, and stress-related psychiatric disorders, in which inflammation is a risk factor. *Mycobacterium vaccae* NCTC 11659 is an environmental saprophyte isolated from mud along the shores of Lake Kyoga in Uganda. It has previously been shown to prevent allergic airway inflammation in murine models of allergic asthma (Zuany-Amorim et al., 2002). More recent preclinical work from our lab also shows that immunization with *M. vaccae* NCTC 11659 induces a number of behavioural responses to stress consistent with prevention of a PTSD-like syndrome. These effects of *M. vaccae* NCTC 11659 include: (1) decreases in stress-induced anxiety-like defensive behavioural responses; (2) prevention of surgery-induced microglial priming and cognitive impairment; (3) prevention of stress- and sleep-deprivation induced behavioural impairments; (4) prevention of stress-induced cortical hyperarousal; and (5) enhancement of fear extinction in the fear-potentiated startle model. 

Together, these data support the hypothesis that exposure to single strains of environmental bacteria with anti-inflammatory and immunoregulatory properties is sufficient to promote stress resilience and prevent endophenotypes relevant to stress-related psychiatric disorders.

Suggested Reading:

- Schultebraucks K, Qian M, Abu-Amara D et al. Pre-deployment risk factors for PTSD in active-duty personnel deployed to Afghanistan: a machine-learning approach for analyzing multivariate predictors. Mol Psychiatry 2020; https://doi.org/10.1038/s41380-020-0789-2.
- Böbel TS, Hackl SB, Langgartner D et al. Less immune activation following social stress in rural vs. urban participants raised with regular or no animal contact, respectively. Proc Natl Acad Sci U S A 2018; 115: 5259–5264.
- Zuany-Amorim C, Sawicka E, Manlius C et al. Suppression of airway eosinophilia by killed Mycobacterium vaccae-induced allergen-specific regulatory T-cells. Nat Med 2002; 8: 625–629.

### 7.8. Urbanized Early Life Microbiota Increases the Risk of Asthma and Atopic Traits

**Jenni Lehtimäki ^1^, Jonathan Thorsen ^2^ and Jakob Stokholm ^2^** (^1^ Finnish Environment Institute, 00790 Helsinki, Finland; ^2^ Clinical Research Unit COPSAC, Ledreborg Alle 34, 2820 Gentofte, Denmark).

Urban populations have higher prevalence of asthma and atopic diseases than rural populations. Accumulating evidence suggests that differing microbial exposures between urban and rural areas can be underlined reason for this difference. However, most of this evidence comes from research focusing on the effect of farming related microbes, especially considering indoor microbiota. 

We studied differences in the infant microbiota and indoor microbiota infants are exposed to in rural and urban children. We utilized birth cohort data from Denmark (COPSAC2010) including 700 prospectively followed children. Asthma, allergic rhinitis, eczema and sensitization are defined by COPSAC paediatricians at six years of age following common criteria. Cohort included hardly any children from farms. 

We found that children born in urban areas had higher risk of asthma and atopic traits at six years of age than rural children. Both airway and gut microbiota differed between urban and rural children, but differences were more pronounced in airways. Indoor microbiota was clearly dissimilar between rural and urban areas. Urbanization of all these microbial communities increased the risk of later developing asthma, and associations with allergic rhinitis, eczema and sensitization were also discovered. Urbanized airway microbiota also produced major shifts in the immune marker concentrations on the lung epithelium. 

With comprehensive data, we show that urban associated microbial communities in infants can mediate increased risk of asthma and atopic traits in urban areas. The potential mechanism for this is disturbed immune development.

### 7.9. What Is a Healthy Skin Microbiome? Leveraging Ancestral Microbiomes for Guidance in Restoration of a Healthy Western Skin Microbiota

**Julia Durack ^1^, Yvette Piceno ^1^, Brian Fanelli ^2^, David Good ^3^ and Larry Weiss ^1^** (^1^ Symbiome, Indiana St, San Francisco, CA 94107, USA; ^2^ CosmosID, Rockville, MD 20850, USA; ^3^ The Good Project 13 Appian Way Cambridge, MA 02138, USA).

Skin microbiota, similar to all other niche-specific human microbial assemblages, are shaped by environmental exposures influenced by lifestyle choices. Growing evidence implicates skin microbiome perturbation in a number of dermatological conditions impacting western society, but how much do we really know about what defines a healthy skin microbiome? To answer this question, we examined the human skin microbiome of individuals from a semi-nomadic hunter–gatherer tribe in the Amazon. Metagenomic sequencing provided insights to the composition and function of a healthy ancestral human microbiome before we were impacted by the urban-industrialized lifestyles of the modern world.

### 7.10. Urban Green Space Aerobiomes: Exposure to Airborne Bacteria Depends upon Vertical Stratification and Vegetation Complexity

**Jake Robinson ^1,2,3^, Christian Cando-Dumancela ^2,3^, Craig Liddicoat ^2,3,4^, Philip Weinstein ^3,4^, Ross Cameron ^1^ and Martin F. Breed ^2,3^** (^1^ Department of Landscape, The University of Sheffield, Sheffield S10 2TN, UK; ^2^ College of Science and Engineering, Flinders University, Bedford Park 5042, Australia; ^3^ The Healthy Urban Microbiome Initiative (HUMI), Adelaide 5005, Australia; ^4^ School of Public Health and the Environment Institute, University of Adelaide, Adelaide 5005, Australia).

Exposure to diverse environmental microbiota is thought to have an important role in “training” the immune system and maintaining human health and wellbeing. Vegetation and soil are both known to be important sources of airborne microbiota, i.e., constituents of the aerobiome. A limited number of studies have attempted to characterise the spatiotemporal dynamics of the aerobiome; however, no known studies have investigated these dynamics from a vertical perspective. Support for its existence can be drawn from studies of pollution and allergenic pollen where vertical stratification occurs at various scales. The existence of aerobiome vertical stratification could have important implications for public health and for the design and management of urban green spaces. For example, do children receive the same exposure to airborne microbiota as adults (or taller humans), and is this influenced by vegetation composition, structure and surrounding land-use?

Furthermore, the potential differences in aerobiome composition based on different land cover types are still poorly understood. In this study we developed and combined an innovative columnar sampling method (using passive petri dish sampling stations), remote sensing techniques, and high-throughput sequencing of the bacterial 16S rRNA gene to assess whether significant vertical stratification of the aerobiome occurs. We also assessed whether there were differences in the aerobiome between land cover types in the Adelaide Park Lands, in South Australia. The land cover types included in the study were as follows: 1. Amenity grassland/lawn; 2. Scrub/trees; 3. Bare ground. Site selection was determined using a combination of remote sensing approaches (such as habitat classification indices and random point algorithms), and ground-based site assessments. We also installed dataloggers to record microclimate data. 

Results presented provide evidence of vertical stratification in both alpha and beta (compositional) diversity of airborne bacterial communities, with diversity decreasing roughly with height. We also found significant vertical stratification in potentially pathogenic and beneficial bacterial taxa. 

Although additional research is needed, these preliminary findings point to potentially different exposure attributes that may be contingent on human height and activity type. Our results lay the foundations for further research into the vertical characteristics of urban green space aerobiomes and their implications for public health and urban planning. 

Further Reading: Jake Robinson, Christian Cando-Dumancela, Craig Liddicoat, Philip Weinstein, Ross Cameron, and Martin Breed. (2020). Vertical Stratification in Urban Green Space Aerobiomes. Environmental Health Perspectives. 128. 10.1289/EHP7807.

### 7.11. Using the Human Skin Microbiota to Measure Nature Exposure in a Longitudinal Study

**Danica-Lea Larcombe ^1^, Eddie Van Etten ^1^, Alan C. Logan ^2^, Susan L. Prescott ^2,3^ and Pierre Horwitz ^1^** (^1^ Edith Cowan University, Joondalup, WA 6027, Australia; ^2^ The Institute for Integrative Health, Baltimore, MD 21231, USA; ^3^ University of Western Australia, Nedlands, WA 6009, Australia).

Evidence indicates that soil (which contains a rich diversity of microbes) is an important component in maintaining human health, and indoor plants have been shown to stabilise the indoor environment. As high-rise apartment dwellers are at a disadvantage in being further away from environmental biodiversity (and reducing the transfer of microbes onto their skin from soil and plants) the question was asked “do soil and plants cause changes in skin microbial diversity and species richness over time”?

This study investigated relationships between high rise apartments, environmental biodiversity and the biodiversity of the human skin microbiota. Fifty-nine eligible participants living in Perth, Western Australia randomly received either three real or artificial indoor plants and were tested for communities of skin bacteria (16S DNA Sequencing) over a 12-month period. Skin microbiota (16S DNA) results from a pilot sample of ten real and fake plant recipients were analysed for respondent’s microbial diversity before and after the study.

The main bacterial phyla were Thermi, Actinobacteria, Bacteroidetes, Firmicutes, Fusobacteria and Proteobacteria. Skin microbiome profiles were unique to each person, and the genus Staphylococcus was found to be dominant (>30%) in the study population, in line with studies from China, Venezuela and Brazil but not the USA. A near significant interaction between time and treatment was found, with participants receiving real plants having a greater increase in OTU richness on average than those receiving fake plants, significant at the ~*p* < 0.1 level. In addition, there was a putative increase in abundance of the Kocuria environmental genus for real plant respondents. 

The intervention appears to have resulted in an increase in OTU richness, and as humans (as a holobiont) are a microcosm of the ecological environment it makes sense that their microbiome would also reflect this habitat that includes bacteria normally residing in soil and plant roots.

### 7.12. Investigating the Effect of Chlorinated Drinking Water on the Assembly of the Infant Gut Microbiome

**Kimberley Parkin ^1^,David Martino ^1^ and Claus Christophersen ^2^** (^1^ Telethon Kids Institute, Nedlands, WA 6009, Australia; ^2^ Curtin University, Bentley, WA 6102, Australia).

The gut microbiome has been particularly interesting in recent years due to its links to health and disease. In infants, the gut microbiome is highly plastic, and easily influenced by environmental factors. The development of a healthy gut microbiome is essential for a stable, diverse, and species-rich gut microbiome as an adult, and improper development of the gut microbiome in this sensitive period can lead to gut dysbiosis. Chlorine is one of the most effective ways to deliver safe drinkable water to the public free from microbial contamination because it produces a residual disinfectant that persists in the distribution system. Due to the antimicrobial effects of chlorine in tap water, this raises the question if persistent exposure to low levels of chlorinated tap water may have a mild antibiotic effect to the diverse ecosystem of microorganisms that colonise the gastrointestinal tract. Thus, residual chlorine in tap water could be a potential unrecognised risk factor of gut dysbiosis. This project will investigate whether chlorine effects the natural spatial and temporal organisation of the gut microbiome in infancy and early childhood.

We hypothesise that persistent exposure to low levels of residual chlorine in early childhood may alter stereotypical colonisation of the infant gut microbiome, by reducing richness and diversity. We will use a randomised control trial study design to install water filters in participant’s homes that remove residual chlorine from tap water. 

We will compare longitudinal changes in the infant gut microbiome from 6 months of age to 18 months of age via faecal samples between the intervention (unchlorinated water) and control (chlorinated water) groups. We will use metagenomics technology to identify novel links between the two groups and early childhood health outcomes, such as asthma and allergies.

### 7.13. Algae as Allies: Learning Algal Patterns to Better Understand Ecosystem Health

**Yogi Hendlin ^1^,Sergio Mungai ^1^, Johanna Weggelaar ^2^ and Nathalia Derossi ^1^** (^1^ Erasmus University College, Burgemeester Oudlaan 50, 3062 PA Rotterdam, The Netherlands; ^2^ Het Nieuwe Instituut, Museumpark 25, 3015 CB Rotterdam, The Netherlands).

Algae are the most prominent generators of oxygen and absorbers of CO_2_ on planet earth. The various forms of algae such as unicellular heterokont algae, diatoms, are also one of the most diverse groups of protists and in addition to fixing carbon and producing oxygen, they generate through photosynthesis basic lipids key to planetary food webs. This presentation surveys human-algae relations through non-instrumental means. Rather than focusing on using algae for fuel production, the dominant technological-scientific discussion of algal applications currently, we ask what do algae need from humans in order to sustain their life-giving ecosystem services?

One of the most underappreciated organisms, discourses around algae currently focus on control, such as geoengineering schemes using iron filing dumping to precipitate plankton algal blooms to draw out masses of oxygen. Such manipulative actions carry unintended counteractions that ignore the complex relationships algae have with sea pH, salinity, water turbidity, and other organisms. Algal quorum sensing allows them abilities to simultaneously be separate organisms, superorganisms, and interspecies conglomerations of organisms. We stand to learn as much or more, we claim, from their communicative and cooperative capacities, as we do from exploiting their metabolic processes.

Looking at Gregory Bateson’s concept of “mindedness” in all living organisms, we inquire into the various ways in which algae mind their habitats, in both senses of the term. How they take care of and create their habitats and circulate resources and energy through directed and passive action is key to understanding how (especially marine) ecosystems sustain and renew themselves. By learning from algae, we suggest that human processes that impinge on algal processes can and must be attenuated through biomimetic technologies and cyclical rather than continuous extraction schedules.

### 7.14. Insights into the Circadian Rhythms on Parasitic Infections and Planetary Health

**Mona El-Sherbini** (Department of Parasitology, Cairo University, Gisa 12613, Egypt).

Planetary health explores the constant interaction between natural and human forces and the resultant state of harmony that arises as an outcome. Of especially increasing importance are the circadian rhythm and night and day cycles governed by the planet earth’s rotation; such automated rhythm appears to play a significant role in system-wide biotics in the atlas of biodiverse species.

We take a look at how modern-day life may have affected human–parasite interactions by studying the effect of circadian rhythms on human immunity and parasite adaptations. Timing matters in all cells of the immune system, orchestration of circadian oscillators as cytokines and chemokines that drive rhythms in synthesis and regulations of various immune responses, influence time-of-day susceptibility to pathogens. For example, the onset of Leishmania infection during late day led to a larger lesion compared to late night infection. Conversely, rhythms in Leishmania parasite load were notably observed in night-time collected blood samples. Clinical and experimental observations have highlighted circadian rhythms of parasites as either intrinsic, generated by endogenous clocks as indicated in Trypanosoma brucei infection or conferred by a rhythmically active vector as in the case of Plasmodium species and microfilariae of Wucheraria bancrofti infections. Rhythmic pattern in the course of infection potentially encodes and predicts circadian environment system and coordinates the parasite’s metabolism, life cycle and transmission with the host’s circadian rhythm.

Therefore, appreciating how external ecosystems interface with internal ecosystems to differentially influence physiology, we can consider circadian biology at the core of human physiology as to anticipate the time of parasitic infection and optimize cellular defence. Hence, better knowledge about host-parasite dynamics may help with new preventive care and personalized treatment strategies in hopes of reducing morbidity and mortality associated with parasitic infections and to achieve harmony between our body clocks and challenges in the outside world.

### 7.15. Can Soil Microbes Modulate the Plasma Metabolome in an Animal Model?

**Saydie Sago ^1^, Ahmed Elsayed ^1^, Christine Foxx ^1^, Jared Heinze ^1^, Antonio Gonzalez ^2^, Fernando Vargas ^2^, Jessica Stewart ^1^, Philip Siebler ^1^, Kyo Lee ^1^, Sandra Appiah ^1^, Morgan Panitchpakdi ^2^, Nicole Sikora ^2^, Kelly Weldon ^2^, Christopher Stamper ^1^, Dominic Schmidt ^1^ and Christopher A. Lowry ^1^** (^1^ University of Colorado Boulder, Boulder, CO 80309, USA; ^2^ University of California at San Diego, La Jolla, CA 92093, USA).

Regulation of the immune system by biodiversity from the natural environment may play an ecosystem service essential to physical and emotional health, in part, through effects on microbiome–gut–brain axis signalling. *Mycobacterium vaccae* NCTC 11659 (*M. vaccae*), a soil-derived bacterium with anti-inflammatory and immunoregulatory properties, is a potentially useful countermeasure against negative outcomes to stressors.

Here, we used male C57BL/6NCrl mice to determine if repeated immunization with *M. vaccae* is an effective countermeasure in a “two hit” stress exposure model of chronically disrupted circadian rhythms (CDR) followed by acute social defeat (SD). On day 28, mice received implants of telemetric recording devices for monitoring 24 h rhythms of locomotor activity. Mice were subsequently treated with a heat-killed preparation of *M. vaccae* (0.1 mg administered subcutaneously on days 21, 14, 7, and 27) or borate-buffered saline (BBS) vehicle. Mice were then exposed to either stable normal 12:12 light:dark (LD) conditions or CDR, consisting of 12 h reversals of the LD cycle every 7 days, for 8 weeks (days 0–56). Finally, mice were exposed to either a 10 min SD or a novel cage control condition on day 54. All mice were exposed to object location memory testing 24 h following SD. The gut metabolome was assessed in faecal samples collected on days 1, 48, and 62 and the host plasma metabolome was assessed on day 64 using LC-MS/MS spectral data. Network-based analysis of the host plasma metabolome suggested that immunization with *M. vaccae* increased the relative abundance of a molecular family of lysophosphatidylcholines, including, 1-heptadecanoyl-sn-glycero-3-phosphocholine.

Together, these data support previous literature demonstrating that increased plasma concentrations of lysophospholipids may be biomarkers of exposures to mycobacterial strains such as *M. vaccae*. Additionally, lysophosphatidylcholines emerge as interesting candidates for mediating some of the physiological and behavioural responses that have been described following immunization with *M. vaccae*.

### 7.16. Do Soil Microbes Influence Stress-Coping Behaviours and Cognitive Performance in an Animal Model?

**Kyo Lee, Jessica Stewart, Christine Foxx, Jared Heinze, Michael Baratta, Ahmed Elsayed, Kelsey Loupy, Mathew Arnold, Philip Siebler, Lauren Milton, Margaret Lieb, James Hassell, Sandra Appiah, Dominic Schmidt, David Duggan and Christopher A. Lowry** (University of Colorado Boulder, Boulder, CO 80309, USA).

Regulation of the immune system by biodiversity from the natural environment may play an ecosystem service essential to health. *Mycobacterium vaccae* NCTC 11659 (*M. vaccae*), a soil-derived bacterium with anti-inflammatory and immunoregulatory properties, is a potentially useful countermeasure against negative outcomes to stressors. Here we used male C57BL/6NCrl mice to determine if repeated immunization with *M. vaccae* is an effective countermeasure in a “two hit” stress exposure model consisting of chronically disrupted circadian rhythms (CDR) followed by acute social defeat (SD). On day 28, mice received implants of telemetric recording devices for monitoring 24 h rhythms of locomotor activity. Mice were subsequently treated with a heat-killed preparation of *M. vaccae* (0.1 mg administered subcutaneously on days 21, 14, 7, and 27) or borate-buffered saline (BBS) vehicle. Mice were then exposed to either stable normal 12:12 light:dark (LD) conditions or CDR, consisting of 12-h reversals of the LD cycle every 7 days, for 8 weeks (days 0–56). Finally, mice were exposed to either a 10 min SD or a novel cage control condition on day 54. All mice were exposed to object location memory (OLM) testing 24 h following SD. Immunization with *M. vaccae* induced a shift toward a more proactive behavioural coping response to SD as measured by increases in scouting and avoiding an approaching male CD-1 male aggressor and decreases in submissive upright defensive postures. In the OLM test, exposure to SD increased cognitive function in *M. vaccae*-immunized CDR mice. Consistent with the hypothesis that immunization with *M. vaccae* altered microbiome–gut–brain axis signalling relevant to stress resilience, *M. vaccae* altered serotonergic gene expression in the dorsal raphe nucleus (DR). Together, these data support the hypothesis that immunization with *M. vaccae* induces a shift toward a more proactive response to stress exposure and promotes stress resilience through microbiome-gut-brain axis signalling.

### 7.17. Effects of Soil Microbe Mycobacterium vaccae on Faecal Microbiomes: Investigating Effects in an Animal Model

**Evan Schaefer ^1^, Sandra Appiah ^1^, Christine Foxx ^1^, Jared Heinze ^1^, Antonio Gonzalez ^2^, Ahmed Elsayed ^1^, Saydie Sago ^1^, Philip Siebler ^1^, Christopher Stamper ^1^, David Duggan ^1^, Kadi Nguyen ^2^, Chloe Gates ^2^, K’loni Schnabel ^2^, Martha Vitaterna ^3^, Fred Turek ^3^ and Christopher A. Lowry ^1^** (^1^ University of Colorado Boulder, Boulder, CO 80309, USA; ^2^ University of California at San Diego, La Jolla, CA 92093, USA; ^3^ Northwestern University, Evanston, IL 60208, USA).

Regulation of the immune system by biodiversity from the natural environment may play an ecosystem service essential to physical and emotional health, in part through effects on microbiome-gut-brain axis signalling. *Mycobacterium vaccae* NCTC 11659 (*M. vaccae*), a soil-derived bacterium with anti-inflammatory and immunoregulatory properties, is a potentially useful countermeasure against negative outcomes to stressors.

Here we used male C57BL/6NCrl mice to determine if repeated immunization with *M. vaccae* is an effective countermeasure in a “two hit” stress exposure model of chronically disrupted circadian rhythms (CDR) followed by acute social defeat (SD). On day 28, mice received implants of telemetric recording devices for monitoring 24 h rhythms of locomotor activity. Mice were subsequently treated with a heat-killed preparation of *M. vaccae* (0.1 mg administered subcutaneously on days 21, 14, 7, and 27) or borate-buffered saline (BBS) vehicle. Mice were then exposed to either stable normal 12:12 light:dark (LD) conditions or CDR, consisting of 12 h reversals of the LD cycle every 7 days, for 8 weeks (days 0–56). Finally, mice were exposed to either a 10 min SD or a novel cage control condition on day 54. All mice were exposed to object location memory testing 24 h following SD. The gut microbiome was assessed in faecal samples collected on days 1, 48, and 62 using 16S rRNA gene sequencing.

Immunization with *M. vaccae* stabilized the gut microbiome, attenuating CDR-induced reductions in alpha diversity. Analysis of microbial beta-diversity revealed community-level separation between the faecal microbiomes of *M. vaccae* and vehicle-treated mice and demonstrated that *M. vaccae* immunization decreased within-group measures of beta diversity.

Together, these data support the hypothesis that immunization with *M. vaccae* stabilizes the gut microbiome in a “two hit” stressor model over several months and is an effective countermeasure against chronic circadian disruption.

### 7.18. Effects of M. vaccae, a Bacterium with Anti-Inflammatory and Immunoregulatory Properties, on Circadian Rhythms of Locomotor Activity in Mice Experiencing Circadian Disruption

**M. C. Flux ^1^, Kyle Kent ^1^, Jared Heinze ^1^, Philip Siebler ^1^, Ahmed Elsayed ^1^, David Duggan ^1^, Dominic Schmidt ^1^, Martha Vitaterna ^2^, Fred Turek ^2^, Monika Fleshner ^1^, Pieter Dorrestein ^3^, Rob Knight ^3^, Kenneth White ^1^ and Christopher A. Lowry ^1^** (^1^ University of Colorado Boulder, Boulder, CO 80309, USA; ^2^ Northwestern University, Evanston, IL 60208, USA; ^3^ University of California at San Diego, La Jolla, CA 92093, USA).

Modern urban lifestyles are frequently associated with chronic disruption of circadian rhythms (CDR). CDR, in turn, has been associated with a persistent exaggeration of inflammatory responses to immune stimulation, which may have negative health outcomes. The soil-derived bacterium *Mycobacterium vaccae* NCTC 11659 (*M. vaccae*), a bacterium with immunoregulatory properties, has been shown to provide protection against negative stress-related outcomes.

In this study, we explored whether these stress-protective effects extend to stress associated with CDR. Adult C57BL/6NCrl mice (N = 112) were equipped with telemetric recording devices on day 28 to continuously monitor locomotor activity throughout the experiment. Mice were immunized with a heat-killed preparation of *M. vaccae* (0.1 mg subcutaneously on days 21, 14, 7, and 27) or borate-buffered saline vehicle. On days 0–56, mice underwent an eight-week CDR paradigm or stable normal 12:12 light:dark (NLD) conditions. Actigraphy data recorded during 8 weeks of CDR/NLD were transformed into weekly periodograms, as calculated via the χ2 method. Dominant periods for each periodogram were identified and modelled as a function of *M. vaccae* and circadian treatment conditions, experimental week, the interaction of circadian treatment and week, and random effects of animal modelled on the intercept.

While there was a significant effect of circadian treatment (F(1,572.0) = 291.97, *p* < 0.001), week (F(1,585.8) = 14.62, *p* < 0.001), and the interaction of week and circadian treatment (F(1,586.0) = 9.41, *p* = 0.002), there was no significant effect of *M. vaccae* treatment on dominant period (F(1,79.4) = 0.002, *p* = n.s.). Overall, dominant periods in the CDR group were higher than the NLD group (CDR mean = 25.6 h, SEM = 0.043; NLD mean = 23.9 h, SEM = 0.007). Simple slope analysis revealed that dominant period increased over the course of the experiment in the CDR group (slope = 0.058, *p* < 0.001) but not the NLD group (slope = 0.006, *p* = n.s.).

These findings suggest that period length was altered by circadian treatment and that this change grew over the course of the experiment but was not altered by *M. vaccae* treatment.

## 8. Personal Ecology in a Rapidly Changing Exposome—What Next!? Health and Fulfilment in a Changing Physical, Emotional, Social and Political Environment

**Figure 7:**
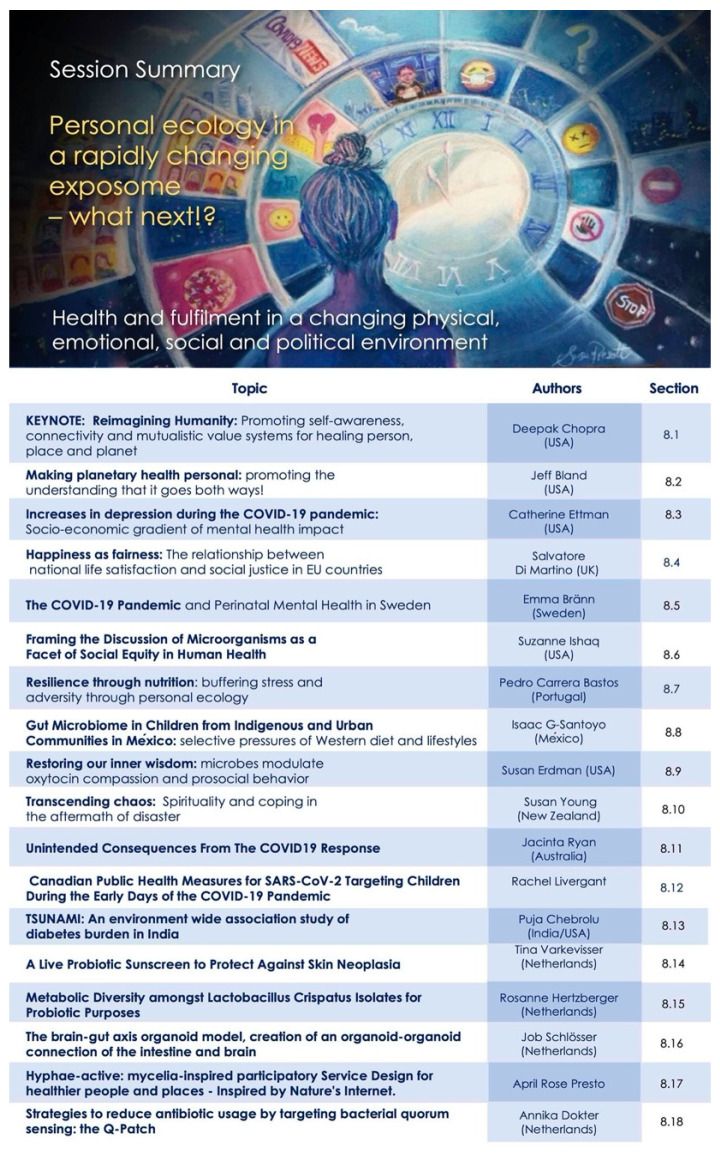
Health and fulfilment in a changing physical, emotional, social and political environment. Overview of topics and speakers in the session on personal ecology in a rapidly changing exposome.

The “exposome” is the total environmental exposures affecting all living systems and their genomes. This occurs in an interdependent, bidirectional manner across the continuum from personal ecology to the wider ecology of physical environments in which all life resides, including the social, economic and political ecology that influences the health of these systems. Personal health therefore depends on addressing both the adverse exposures eroding health, and promote positive exposures for resiliency, which extend from biological influences to psychological assets and value systems that determine policies and practices. This session explores these interrelationships and opportunities for meaningful change (Figure 7).

### 8.1. KEYNOTE: Reimagining Humanity: Promoting Self-Awareness, Connectivity and Mutualistic value Systems for Healing Person, Place and Planet

**Deepak Chopra** (Clinical Professor of Family Medicine and Public Health at the University of California, San Diego; founder of The Chopra Foundation, La Costa, CA 92009, USA).

No abstract available: a recording of Dr Deepak Chopra’s inspirational Keynote Lecture is available for viewing through our online proceedings [12].

### 8.2. Making Planetary Health Personal: Promoting the Understanding That It Goes Both Ways!

**Jeffrey Bland** (President, Personalized Lifestyle Medicine Institute, Bainbridge Island, Seattle, WA 98110, USA).

The viral infection known as SARS-CoV-2 and the worldwide COVID-19 pandemic have been a frame-shifting event for all humans living on planet Earth. One of the key things we have learned is that not all people exposed to the SARS-CoV-2 virus have equal risk to the COVID-19 disease. The presence of certain reducible risk factors associated with chronic disease—specifically obesity, diabetes, hypertension, and dyslipidemia—are considered to be significant factors that increase the pathogenicity of the virus. Because these risk factors are common in the modern world, we essentially were already experiencing a pandemic of chronic disease when the SARS-CoV-2 virus began to spread. This widespread state of compromised health is what to led to the concerning progression to COVID-19 disease and the serious pandemic we have witnessed over the past year—in essence, COVID-19 is a pandemic within a pandemic.

Researchers have determined that risk to COVID-19 is associated not only with biological factors, but also social issues, such as isolation, compromised access to good nutrition, poverty, loneliness, and poor physical environment, which can include exposure to toxins, as well as stressful social and community context. What is the connection? All of these factors contribute to altered immune status, which can lead to increased vulnerability to viral infection and disease severity.

Is there a root cause that links disturbances of immune system function to the comorbidities that have become a hallmark of the COVID-19 pandemic? I propose that it relates to disturbances in the planetary immune system—that essential network that connects microbes, plants, animals, and humans. Each living thing has its own immune system, and we are all interconnected through 24/7 communication networks. When disturbances occur at the planetary level in the systems that maintain homeodynamic balance of the macrocycle, they work their way downstream into perturbations in the regulatory systems that control the defence, repair, and regeneration processes of every microbe, plant, and animal. In a sense, there is a planetary immune system that connects all systems together, both living and non-living.

This concept I am describing is based on a field of study called macroimmunology. It is now well recognized that the function of plant immune systems is connected to both biotic and abiotic factors. Environmental pollution, global warming, and climate change modify the function of a plant’s innate immune system through changes in genetic expression of stress-responsive genes. The immune systems of plants consist of the type and amounts of various phytochemicals within them, and these substances can impact human immune systems when they are consumed as food. Diets of highly processed foods that are devoid of immune-active phytochemicals can result in compromised immune system reserve. The fact that low quality dietary habits have become commonplace has led to the increasing prevalence of the immune-stressed phenotypes of obesity, diabetes, and inflammatory disorders. Stressed immune systems have been linked to inflammaging, which is a metabolic condition associated with all of the reducible comorbidities that have been found to be significant to COVID-19. Dr. Francis Collins, Director the National Institutes of Health in the United States, has stated the following: “Exploring how diet and nutritional status modify the immune response could help explain some of the variability in the COVID-19 morbidity and mortality, even in individuals without diet-related disease” (see Rodgers et al. in suggested reading).

The takeaway is this: the planet, microbes, plants, animals, and humans are all interconnected through the integrity of their shared immune systems. Planetary health should therefore be aligned with a pledge to unite health professionals in the age of the Anthropocene in supporting the connection among planetary abiotic and biotic systems and making a commitment to establishing the functional integrity of the immune systems of all people.

Suggested Reading:

Sheldon TA, Wright J. Twin epidemics of covid-19 and non-communicable disease. BMJ. 2020 Jun 30; 369: m2618.

Belanger MJ, Hill MA, Angelidi AM et al. Covid-19 and Disparities in Nutrition and Obesity. N Engl J Med. 2020 Sep 10; 383 (11): e69.

Franceschi C, Bonafè M, Valensin S, et al. Inflamm-aging. An evolutionary perspective on immunosenescence. Ann N Y Acad Sci. 2000 Jun;908: 244–54.

Rodgers GP, Collins FS. Precision Nutrition-the Answer to “What to Eat to Stay Healthy”. JAMA. 2020 Aug 25; 324 (8): 735–736.

### 8.3. Increases in Depression during the COVID-19 Pandemic: Socio-Economic Gradient of Mental Health Impact

**Catherine Ettman** (Boston University School of Public Health, Boston, MA 02118, USA).

While the COVID-19 pandemic has upended daily life for all people, the economic and mental health consequences of the moment have not been borne equally. This presentation reported on some of the first findings published in the US on depression severity experienced during the pandemic relative to before COVID-19, and emerging trends for increasing inequities. This talk explores leading edge data on mental health, assets, and what we can learn from this moment to build back a better world post-pandemic.

Further Reading:

Ettman et al. Prevalence of Depression Symptoms in US Adults Before and During the COVID-19 Pandemic. JAMA Network Open. 2020; 3 (9): e2019686

### 8.4. Happiness as Fairness: The Relationship between National Life Satisfaction and Social Justice in EU Countries

**Salvatore Di Martino ^1^and Isaac Prilleltensky ^2^** (^1^ Division of Psychology, Faculty of Management Law and Social Science, University of Bradford, Bradford BD7 1DP, UK; ^2^ Vice Provost for Institutional Culture, Erwin and Barbara Mautner Chair in Community Wellbeing, University of Miami, Coral Gables, FL 33124, USA).

In recent years, we have witnessed an ever-growing release of books, TED talks, websites, and scientific articles that promote strategies to achieve long-lasting happiness. Some are based on the idea of self-help, self-improvement, and life coaching, some draw from ancient and modern philosophical teaching, some promote spiritual practices such as Buddhism, others rely on modern scientific findings—from neuroscience to positive psychology—and some a combination of all these.

What most of these approaches have in common, is the idea that happiness is something we can achieve if we put our mind and efforts into it, because ultimately happiness is a choice, and individuals are the only ones responsible for their own actions. This also means that what matters most to enjoy a happy life is less about the objective world outside of us, and more about the way we look at the world. It is our attitude towards life that must change if we want to be happy.

This kind of approach also finds confirmation in the mainstream wellbeing scholarship, which has often treated happiness as a personal matter, an individual state of mind that comes more from within than from without. Some scholars, for instance, attach an extremely low weight to external circumstances in determining life satisfaction, believing that what it counts most is people’s right predisposition towards life. This attitude has resulted in a strong focus on the individual determinants of life satisfaction and an overlook of the environmental, social, and political factors that play a significant role in either fostering or hindering people’s happiness. Conversely, we argue that, when it comes to happiness, we cannot shy away from considering conditions of social justice. When we talk about social justice, we refer to the equal distribution of resources and life-fulfilling opportunities (distributive justice), fair and transparent decision-making (procedural justice), and the rightful allocation of rewards and punishments (retributive justice) in society.

To demonstrate the thesis that social justice is beneficial to people’s happiness, we presented results from a study that compared the EU Social Justice Index (a measure of social justice developed by the Bertelsmann Stiftung), with levels of life satisfaction taken from several waves of the Eurobarometer, across 28 EU countries. Our analyses also included relevant predictors of national happiness including Gross Domestic Product (GDP), social capital, level of individualism versus collectivism, and being or not a post-communist country. Our findings offer strong evidence that social justice has an impact on people’s happiness, much more than GDP. In fact, those countries that score lower on the EU Social Justice Index tend to show lower levels of life satisfaction, and vice versa.

These findings are relevant to raise awareness within the wellbeing scholarship and beyond of the importance of promoting conditions of social justice for the betterment of societies and the happiness of their people.

### 8.5. The COVID-19 Pandemic and Perinatal Mental Health in Sweden

**Emma Bränn ^1^,Emma Fransson ^1^, Mary Kimmel ^2^, Fotios C. Papadopoulos ^3^ and Alkistis Skalkidou ^1^** (^1^ Uppsala University Women’s and Children’s Health, Akademiska sjukhuset, 751 85 Uppsala, Sweden; ^2^ Department of Psychiatry, School of Medicine, University of North Carolina, Campus Box #7160, Chapel Hill, NC 27599, USA; ^3^ Department of Neuroscience, Psychiatry, Uppsala University, 751 85 Uppsala, Sweden).

The COVID-19 pandemic has thus far claimed the lives of more than 1 million people worldwide. Today, more than 36 million people have been infected and among them pregnant women and new parents. For this group, the pandemic could take on additional dimensions including worry for the pregnancy and the infant, which implies additional risk for developing mental ill-health during the already critical perinatal period. Perinatal depression is an overlooked and underdiagnosed condition affecting 12% of the pregnant population. The need for early detection and intervention have become evident and the pandemic generates further obstacles to an already vulnerable population.

Therefore, the aim of this study was to investigate the impact of the COVID-19 pandemic on the mental health of pregnant women in Sweden using the research mobile application, Mom2B (mom2b.se). Data on COVID-19 related symptoms, testing, diagnosis and how much the pandemic has affected everyday life, as well as symptoms of depression and anxiety administrated by the Edinburgh postnatal depression scale (EPDS and EPDS-A), were assessed in 1340 pregnant women since the end of 2019. No direct association between a possible COVID-19 diagnosis and depression was observed. However, the percentage of women scoring higher than the validated cut-off (≥13 points) increased during the months with high death tolls and restrictions in maternity care due to the pandemic in Sweden, from ~16% in November–January to ~23% in April-June. Furthermore, increased anxiety was observed among women reporting everyday life to be moderately to highly affected by the pandemic.

Our results indicate pregnant women’s mental health being significantly affected during months with the greatest pandemic related impact in Sweden, despite the absence of lockdown measures. As mental ill-health during pregnancy is a major contributor to postpartum depression, these results need to be taken into consideration when planning for future societal resource distribution.

### 8.6. Framing the Discussion of Microorganisms as a Facet of Social Equity in Human Health

**Suzanne Ishaq** (University of Maine, College Ave, Orono, ME 04469, USA).

What do “microbes” have to do with social equity? Microorganisms are integral to our health, that of our natural environment, and even the “health” of the environments we build. The loss, gain, and retention of microorganisms—their flow between humans and the environment—can greatly impact our health. It is well known that inequalities in access to perinatal care, healthy foods, quality housing, and the natural environment can create and arise from social inequality. Do we have a right to microbes? In the age of host-associated microbiomes, diet and gut health, urbanization and the indoor generation, and human influence on microbial communities, perhaps we need to re-imagine public policy which takes microorganisms into account.

In a four-week course in the summer of 2019, I introduced 15 undergraduates from the University of Oregon Clark Honors College to microorganisms and the myriad ways in which we need them. We talked about how access to nutritious foods (and especially fibre), pre- and postnatal health care, or greenspace and city parks, could influence an individual’s microbial exposures over a lifetime. Inequalities in that access—such as only putting parks in wealthier neighbourhoods—creates social inequity in resource distribution, but it also creates inequity in microbial exposure and the effect on health. By the end of the that four weeks, the students, several guest researchers, and I condensed these discussions into a single essay.

Importantly, nearly all the students were from diverse humanities fields. The course itself became a model on the feasibility of connecting science to the humanities. Similarly, this presentation explores the idea of microbes and social equity, and sparks discussion on our role as scientists in public policy, with the goal of connecting researchers to generate actionable items on how we can use our understanding of microbial ecology to inform public policy.

### 8.7. Resilience through Nutrition: Buffering Stress and Adversity through Personal Ecology

**Pedro Carrera Bastos** (Department of Clinical Sciences, Lund University, Malmö, Sweden).

Psychological and physical resilience can be affected by many factors, which although apparently unrelated can act through common mechanism—inflammation. Nevertheless, it should be mentioned that acute inflammation is actually an evolutionarily conserved process whose goal is to protect the host from infections and other insults and also to promote tissue repair, restoring homeostasis. Inflammation is thus a crucial physiological response characterized by the recruitment of various immune and non-immune cells. Since these activated cells will have increased energy requirements, as well as specific nutrient needs, there will be competition for those resources between the immune system and other organs and systems (such as the muscle, the adipose tissue and the central nervous system, among others). Therefore, various metabolic, and neuroendocrine changes must occur to supply more nutrients to the activated immune system at the expense of other organs, while at the same time limiting nutrient (such as iron) access to infectious organisms. These adaptations, which include insulin resistance, dyslipidaemia, increased blood pressure, nutritional perturbations, bone and muscle losses, anorexia, fatigue, decreased libido and fertility, depression-like symptoms, and sleep disturbances cannot be chronic or risk compromising survival and reproduction. As such, a healthy acute inflammatory response is time-limited and resolves once the trigger ceases.

Nevertheless, variety of lifestyle and environmental factors, such as smoking, pollution, psychological stress, sleep disturbance and circadian disruption, physical inactivity, and western-type hypercaloric diets (which are high in sugar, refined grains, alcohol, salt, trans fatty acids, oxidized lipids, and advanced glycation end-products, and low in various micronutrients, fibre, prebiotics, omega-3 fatty acids and phytochemicals), coupled with excessive adipose tissue, increased intestinal permeability and dysbiosis can persistently activate numerous inflammatory pathways, leading to a state of low-grade chronic inflammation (LGCI). In turn, LGCI can compromise resilience directly or indirectly, since it might cause hypertension, type 2 diabetes, non-alcoholic fatty liver disease, coronary heart disease, stroke, osteoporosis, sarcopenia, various types of cancer, endocrine disorders, depression, and neurodegenerative diseases. Moreover, it contributes to the exacerbation and perpetuation of osteoarthritis and chronic kidney disease, disturbs the status of various micronutrients, and compromises the adaptive immune function, precluding an adequate response to infectious agents and vaccines and the establishment of immunological tolerance to self-antigens (thereby increasing the risk for autoimmune diseases).

Therefore, lifestyle and nutritional interventions that prevent, decrease, and resolve inflammation are of paramount importance, especially under the current stressful context (e.g., the coronavirus pandemic).

### 8.8. Gut Microbiome in Children from Indigenous and Urban Communities in México: Selective Pressures of Western Diet and Lifestyles

**Isaac G-Santoyo** (National Autonomous University of México, University City, Coyoacán, Mexico City 04510, Mexico).

We are holobionts, which means that we are a biological unit formed by our cells plus associated microbes. In this relation, the human gut microbiome (GM) needs to be considered an essential component that defines host health. Lifestyle practices that we have experienced recently may impact the ecosystem dynamics of our GM. These practices can result in selective pressures for the GM that has co-evolved with us since our origins as species. For instance, measures to kill or limit exposure to pathogenic microbes, such as the excessive use of antibiotics, combined with other factors such as ultra-processed foods containing preservatives and additives. These selective pressures are significant for Childhood since it is a sensitive period for establishing and developing this microbial ecosystem. In this study, we sequenced the 16S rRNA gene from faecal samples of children between 5 and 10 years old in two Mexican communities with contrasting lifestyles; inhabitants of Mexico City, the most industrialized in Mexico, and an indigenous pre-Hispanic population of the ethnic group named Mep’haa. Births in these communities are through natural delivery, and children are breastfed to the age of two. There is almost no access to allopathic medications, and there is no health service, or water purification system. Food is based on beans, lentils and corn. Wild edible plants are also collected, and some fruits and vegetables are cultivated in garden plots. Meat is consumed almost entirely during special occasions and is not part of the daily diet.

The main differences found were in bacteria associated with different diets, high animal protein, refined sugars, and high fibre food. Besides, the GM of Me’phaa children showed higher total diversity and the exclusive presence of 11 phyla. In contrast, the children from México City showed less diversity and the exclusive presence of 1 phylum associated with the degradation of sugar compounds. Hence, there was a clear GM separation between these populations sharing only a quarter of the total species. The higher diversity and dissimilarity found in Me´phaa children could induce GM stability after disturbance periods because it is possible to have different bacteria groups with similar functions, resulting in a Functional Redundancy. These results provided further knowledge about how changes in sociocultural practices have been selective pressures for the GM, and how these changes are associated with pathophysiological states usually present in westernization lifestyles.

### 8.9. Restoring Our Inner Wisdom: Microbes Modulate Oxytocin Compassion and Prosocial Behaviour

**Susan Erdman** (Massachusetts Institute of Technology, Cambridge, MA 02139, USA).

Our inner wisdom helps us navigate traumatic life events during social and environmental upheavals. Mounting scientific evidence implicates a gut–immune–brain axis in our sense of self, raising the possibility that our microbial passengers and hormone oxytocin offer both resiliency and a sense of connectedness to sustain life during challenging times. Earlier work in animal models revealed that exposure to a commensal human milk microbe, *Lactobacillus reuteri*, served to elevate endogenous levels of oxytocin, a neuropeptide hormone otherwise associated with reproduction and feelings of sociability, trust, and empathy. We postulate that our holobiont, including symbiotic microbiota, together with hormone oxytocin stimulate nurturing near birth with a cascade of individual, familial, and societal outcomes of wellbeing. Additionally, oxytocin emerges as a candidate key to unlocking our navigational inner wisdom. In this paradigm, oxytocin-mediated neuronal engagement forms the basis of our spirituality, gut feelings and intuitions. Through our ancestral experiences, microbes and microRNAs build a vast universal wisdom in neurological archives to help us navigate through difficult emotional and spatial journeys to find our way back to the safety of community. In this way, distant forebear holobionts gift us with resiliency and inner wisdoms that take care of us all.

### 8.10. Transcending Chaos: Spirituality and Coping in the Aftermath of Disaster

**Susan Young** (Lincoln University, Lincoln 7647, Christchurch, New Zealand).

When an “I thought I was going to die quake” occurs amidst four additional major earthquakes and 15,000 aftershocks during a sixteen-month period, it challenges people’s ability to cope and recover. Residents of Canterbury, New Zealand endured this extended, chaotic state in 2010/11; and continue to deal with lingering effects on their devastated central city, Christchurch. Stress and coping theory suggests that finding meaning in such situations can help people recover, and that religion and spirituality often play a role in post-disaster resilience. This research focuses on spirituality in its most general form and explored how it may have contributed to people’s coping and recovery after the earthquakes. Sixteen people were interviewed and their storied accounts thematically analysed to understand each individual’s meaning construction and coping/recovery process and identify connective themes and patterns amongst their experiences. Four core elements of acceptance, clarity and choice, connection, and transcendence emerged from the thematic analysis to conceptualize a model of transcendent coping. This research contributes to the growing interest in spirituality as an important facet of human nature that can support wellbeing in the face of ongoing stress. Harnessing this innate wisdom and strength is easily done by thinking about what each of us does, in our own way, to bring us peace.

When dealing with stress or even during good times, living well could involve tapping into these transcendent moments more often which ultimately results in more kindness—for ourselves and others.

### 8.11. Unintended Consequences from the COVID-19 Response

**Jacinta Ryan ^1^,Jeffrey Craig ^2^ and Wayne Leifert ^3^** (^1^ RMIT University, La Trobe St, Melbourne, VIC 3000, Australia; ^2^ Deakin University, Burwood, VIC 3125, Australia; ^3^ CSIRO, University of Adelaide, Adelaide, SA 5000, Australia).

As significant resources are committed in the race for a pharmacological solution to the COVID-19 emergency, questions need to be asked as to the potential risks a treatment or preventative could pose to the individual and the community due to unintended consequences.

What do we know? We know is that pharmaceuticals can be toxic to biological systems, resulting in disease. This toxic effect can be through damage to DNA, otherwise known as a “genotoxic effect”. This has been linked to major chronic diseases (e.g., cancer, cardiovascular, neurodegenerative diseases) as well as generational changes contributing to disease in offspring. OECD Guidelines for Testing of Chemicals require genotoxicity testing for potential new and existing pharmaceuticals. This currently does not apply to pharmaceuticals classed as biologics (e.g., whole vaccines).

What are we getting to know? New insights into the genome’s 4D landscape are emerging raising new concerns relating to potential genotoxic risks created by the introduction of pharmaceuticals. As our growing understanding of the complexity of the genome architecture unfolds, the mechanism by which pharmaceuticals can create risk to the biological system through genome damage are increasingly being revealed.

Why the need for caution? If what we know provides cause for concern, what we are getting to know should create even greater concern as it relates to pharmacological responses to the COVID-19 crisis. It is essential that, in our haste to find a solution to the immediate threat created by COVID-19, the solution does not establish the cellular conditions that could create long term and generational disease risk for other serious health conditions. It is essential that we ensure potential interventions are appropriately understood and evaluated as not to create immediate and long-term unintended consequences not only in otherwise healthy individuals but also to their offspring.

### 8.12. Canadian Public Health Measures for SARS-CoV-2 Targeting Children during the Early Days of the COVID-19 Pandemic

**Rachel Livergant ^1^, Julie Polisena ^2^, Omolara Sanni ^1^, Brittany Matenchuk ^1^, Sana Amjad ^1^, Liz Dennet ^1^, Igor Zoric ^1^, Nisrine Haddad ^3^, Andra Morrison ^2^, Wilson Kumana ^3,4,^, Isaac Bogoch ^5^, Vivian Welch ^6^ and Maria Ospina ^7^** (^1^ School of Public Health, University of Alberta, Edmonton, Alberta, Canada; ^2^ Canadian Agency for Drugs and Technologies in Health, Ottawa, Ontario, Canada; ^3^ School of Epidemiology and Public Health, University of Ottawa, Ottawa, Ontario, Canada; ^4^ Clinical Epidemiology Program, Ottawa Hospital Research Institute, Ottawa, Ontario, Canada; ^5^ Divisions of General Internal Medicine and Infectious Diseases and Department of Medicine, University Health Network, Toronto, Ontario, Canada; ^6^ Centre for Global Health, Bruyere Research Institute, Ottawa, Ontario, Canada; ^7^ Faculty of Medicine and Dentistry, University of Alberta, Edmonton, Alberta, Canada).

Introduction: On March 11, 2020, the World Health Organization declared a global pandemic in response to the severe acute respiratory syndrome coronavirus 2 (SARS-CoV-2), which is the causative agent of the novel coronavirus disease 2019 (COVID-19). Accordingly, health officials in Canada issued public health measures at the federal and provincial/territorial (PT) level to contain and mitigate SARS-CoV-2 transmission in the population and prepare the health. Children were identified as a critical group during the pandemic, due to their propensity to act as asymptomatic carriers and transporters of the virus.

Objective: This scoping review describes public health measures implemented at the start of the global COVID-19 pandemic in Canada, with an emphasis on the extent to which issued public health measures targeted children.

Methods: We conducted a comprehensive search strategy of federal and P/T websites on COVID-19 public health preparedness strategies that were released between 30 January 30 and 30 April 2020. Specific measures targeting children under the age of 18 were identified and examined. A mixed-methods approach for analysis of results was conducted, using both quantitative and qualitative methods.

Results: Of the 722 public health measures implemented during the study period, 8.7% (63) targeted children. Prince Edward Island and Quebec issued the most measures (8) while Yukon, Nunavut, Northwest Territories, British Columbia and Manitoba issued the least (three each). The majority of measures focused on school and university closures (28, 44%), with daycare closures the next most common measure (14, 22%). Most of the public health measures targeting children were mandatory orders issued by governments (53, 84%). Non-mandatory measures focused on social supports for children and parents, including daycare provisions for essential workers and recommendations to keep children home from school after travelling in the first few weeks of the pandemic. Most of the public health measures were issued in March 2020 (45, 71%), while none were issued in January and only one measure was introduced in February. Eleven (17%) public health measures aimed at mitigating the psychological, social and/or educational stressors of the pandemic on children.

Conclusion: This scoping review provides insights on the effects of public health measures issued by the Canada federal and P/T government in the first 90 days of the pandemic on children. Several of these measures directly impacted children’s education and care, however, an analysis on the evolution and impact of these measures merits further investigation.

### 8.13. TSUNAMI: An Environment Wide Association Study of Diabetes Burden in India

**Puja Chebrolu ^1^, Anjali Radkar ^2^, Atul Kumar Sahai ^3^, Morteza Khafaie ^4^, Pooja Arora ^5^, Shraddha Surana ^5^, Susmitha Joseph ^3^ and Chittaranjan Yajnik ^6^** (^1^ Weill Cornell Medicine, 1300 York Ave, New York, NY 10065, USA; ^2^ Gokhale Institute of Politics and Economics, Pune, Maharashtra 411004, India; ^3^ Indian Institute of Tropical Meteorology, Panchawati, Pashan, Pune, Maharashtra 411008, India; ^4^ Ahvaz Jundishapur University of Medical Sciences Khuzestan Province, Ahvaz 61357, Iran; ^5^ ThoughtWorks Technologies, Gurgaon 122002, India; ^6^ Diabetes Unit, King Edward Memorial Hospital and Research Center, Pune, Maharashtra 411011, India).

Background: Type 2 diabetes (T2D) has complex aetiology, including genetics, intrauterine epigenetic programming and life course environmental exposures. Traditional risk factors (diet and physical activity) explain only a part of the susceptibility. Substantial proportions of Indian diabetic patients are non-obese and are physically active. Environmental factors may play a significant role but are poorly studied. An Environment-Wide Association Study (EWAS) such as that performed in the US by Patel et al. (PLoS ONE 2010) may provide direction for future studies. We aim to conduct an India-wide EWAS of T2D using data on air, water and soil pollution, weather patterns and population health-developmental indices.

Methods: We will use conventional statistics and machine learning methods and visualizations to correlate rise in T2D prevalence to changing environmental exposures from 1990 to 2016. Data will be obtained from multiple public sources. Diabetes prevalence will be obtained from the GBD India and INDIAB studies conducted between 1990 and 2016. Other data sources are: the Indian National Family Health Survey, air pollution data from the Indian Central Pollution Control Board (PM 2.5 and 10, NO_2_, and SO_2_ levels) by state from 2000 to 2016, POP exposure estimated using a composite measure of levels of individual POPs in groundwater and milk from 1970 to 2000, water pollution data including heavy metal contamination of groundwater between 1970 and 2000, and weather data including mean rainfall and average temperature from 2000 to 2016. Given the long exposure time to cause disease, POPs and heavy metals (and other exposures) will be assessed 20 years prior to available T2D data.

Expected Findings: Outcomes will include the association of environmental factors with rise in T2D prevalence between 1990 and 2016. This will provide vital data for generation of novel hypotheses to study the association of these factors with T2D risk.

### 8.14. A Live Probiotic Sunscreen to Protect against Skin Neoplasia

**Tina Varkevisser** (Vrije Universiteit, 1081 HV Amsterdam, The Netherlands).

The skin microbiome has been studied for its role in protecting the host against invading pathogens, educating our immune system, and breaking down natural products on the skin. *Staphylococcus epidermidis* is a prominent bacteria found on our skin, and recent findings have elucidated its role in skin cancer protection. A commensal strain of *S. epidermidis* produces the compound 6-N-hydroxyaminopurine (6-HAP) which was shown to selectively inhibit DNA polymerase function of skin tumour cells, leaving primary keratinocytes unharmed. Topical treatment with this strain of *S. epidermidis* in mice has shown a significant reduction in the incidence of UV-induced tumours. Moreover, intravenous injection of 6-HAP in mice was able to inhibit the growth of B16F10 melanoma without systemic adverse drug reaction. With this knowledge, this project aims to develop a safe and effective sunscreen containing a live commensal strain of *S. epidermidis* to reduce the risk of UV-induced skin cancer. We hypothesise that the topical application of this product will potentially reduce the incidence of skin cancer through the dual benefit of the 6-HAP produced by *S. epidermidis* and sun protection factor.

### 8.15. Metabolic Diversity amongst Lactobacillus Crispatus Isolates for Probiotic Purposes

**Rosanne Hertzberger,Heleen Eising and RemcoKort** (Vrije Universiteit, 1081 HV Amsterdam, The Netherlands).

The microbiome of the human vagina has low diversity compared to other mammals and to other bodily niches: most women are dominated by a small group of Lactobacillus species. Absence of these bacteria is regarded as a dysbiotic state with increased diversity, an elevated pH, exfoliation of the vaginal epithelium and degradation of glycogen, which is the most abundant vaginal carbohydrate. Besides abnormal discharge, vaginal dysbiosis (or “bacterial vaginosis”) is associated with adverse sexual and reproductive health outcomes. Especially women who are colonized by *Lactobacillus crispatus* are less likely to acquire sexually transmitted disease and when pregnant are at lower risk of preterm labour.

Amidst the global epidemic of antibiotic resistance, bacterial vaginosis is still commonly treated with metronidazol. Here we propose to transfer from an antibiotic approach towards a “women for women”—probiotic approach. We have isolated 19 strains of *Lactobacillus crispatus* from various reproductive-age women and studied their metabolic diversity. Thirteen out of nineteen were capable of utilizing glycogen for acidification. The six non-glycogen consuming strains all carried a deletion in the N-terminal signal peptide sequence of a putative cell surface associated type 1 pullulanase. This enzyme showed high similarity to the extracellular cell wall attached pullulanase previously found in a human gut isolate of *Lactobacillus acidophilus*.

In the future, we aim to utilize this collection of metabolically diverse strains in a probiotic mixture to facilitate women to share vaginal microbial health amongst each other.

### 8.16. The Brain–Gut Axis Organoid Model, Creation of an Organoid–Organoid Connection of the Intestine and Brain

**Job Schlösser ^1^and Remco Kort ^2^** (^1^ University of Amsterdam, 1000 GG Amsterdam, The Netherlands; ^2^ Vrije Universiteit, 1081 HV Amsterdam, The Netherlands).

The bidirectional brain–gut axis demonstrates itself as a prominent controversial mystery within the scientific community. Various associations are made between neuropathology/mental health and the gut microbiome. However, current experimental tools lack the capability of direct and robust conclusions for the brain–gut axis functioning. By introducing the possibility to connect the human brain organoid (HBO) and the human intestinal organoid (HIO), this study could provide new insights in the scientific investigation of the brain–gut axis. This brain–gut axis organoid will be connected via the vascular system, nervous system and autologous immune cells. Starting from various progenitor cell combinations, to strategical finetuning by state-of-the-art biomimetic methods. Moreover, the vascular system will be connected to a blood/vascular controlling apparatus providing the brain–gut axis organoid a steady state (including essential lung and heart functioning abilities) and quantitative abilities. This newly formed organoid connection will create various experimental approaches and applications to intervene with the organization of the brain–gut connection. Although its experimental ambitious nature, recent research provided sufficient possibilities to fulfil this brain–gut axis organoid, thereby contributing to future insight in the interesting field of the brain–gut axis research.

### 8.17. Hyphae-Active: Mycelia-Inspired Participatory Service Design for Healthier People and Places—Inspired by Nature’s Internet

**April Rose Presto** (Center for Interventions toward Development and Empowerment through Allied Services, (I.D.E.A.S.), Inc./1231, University of the Philippines, 1101 Quezon City Manila, Philippines).

Participatory service design is recognized as beneficial in learning about and engaging with clients. Individual narratives, much like a hyphae, make up a vibrant network of places, objects, and experiences—data that can be utilized to create programs that enhance quality of life of people and families with neurodiversities (i.e., family member with medical conditions such as ADHD, ASD, or other developmental challenges). By co-designing the process with clients, relationships are established and ecosystems in which the relationships take place are better understood. This mycelia-inspired journey documents the experience of a clinical facility in the creation of a food journey map linking food practices, environment, and food systems to aid in the assessment and intervention phases of the occupational therapy process.

Eating and meal preparation are seen as meaningful occupations that sustain family life. Understanding our clients with neurodiverse abilities and their families’ interaction with food not only provided information about sensory–motor challenges in eating—the typical reason for referral to occupational therapy services—but also revealed insights about food habits (e.g., where it is sourced, who prepares it, temporal dimensions, etc.) and environments (e.g., home, restaurants, parks, groceries etc.) where microbiomes can interact. Good nutrition and nature contacts have a positive influence on learning and behaviour and can therefore be incorporated as nature services in the occupational therapy process.

This service design aims to go deep and beyond grassroots interaction and reflect on the connections within a mycelium internet. From client to clinician to other members of the medical team (such as speech-language therapists, physicians, nutritionists, etc) to food systems and environments, each connection is established to inform and nurture each other’s role in the ecosystem of health and wellbeing—not only of individuals and families but of communities and our planet.

### 8.18. Strategies to Reduce Antibiotic Usage by Targeting Bacterial Quorum Sensing: The Q-Patch

**Annika Dokter** (Vrije Universiteit, 1081 HV Amsterdam, The Netherlands).

Chronic wounds containing biofilm have a major impact on today’s society, both in terms of morbidity and economic costs. Current treatment is suboptimal and lengthy use of systemic antibiotics is common, leading to resistance and unwanted side effects such as dysbiosis of the microbiome. New ways to prevent and treat biofilm formation in chronic wounds are urgently needed. A possible target is quorum sensing, a communication system between bacteria that orchestrates biofilm formation. The development of a plaster impregnated with the quorum sensing inhibitor thionine T310 can bring an effective, easy-to- use treatment for patients suffering from chronic wounds and thereby lower the use of antibiotics.

## 9. Across the Ages—Transgenerational and Life-Course Opportunities to Improve Health: Developmental Origins—Part 1

**Figure 8:**
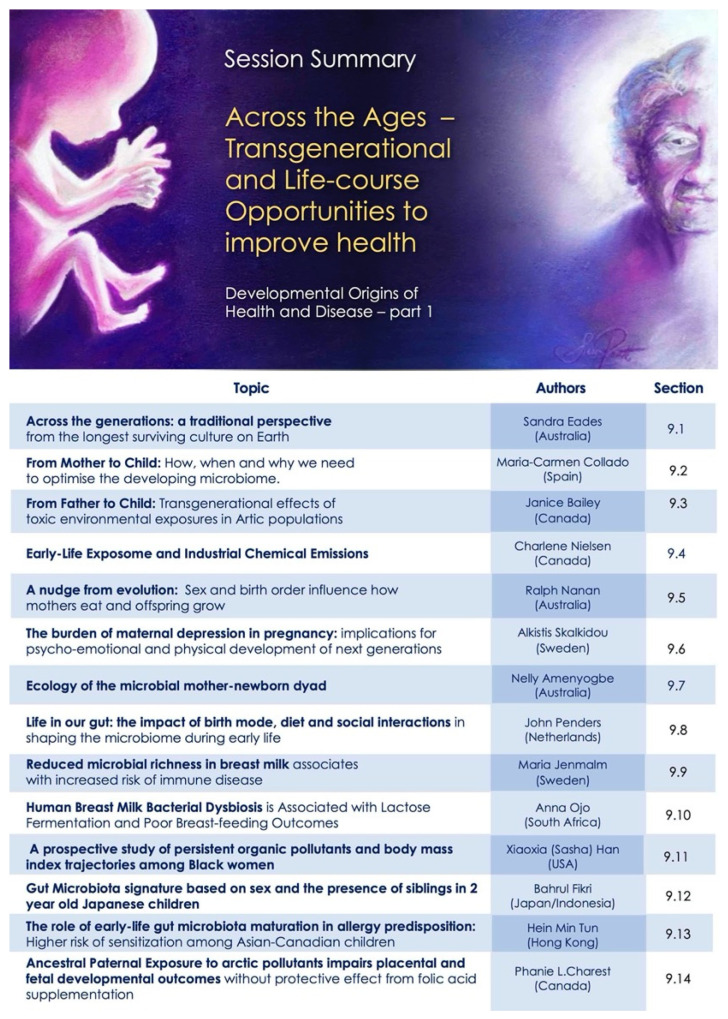
Transgenerational and Life-course Opportunities to Improve Health: Overview of topics and speakers in the session on developmental ecology (Part 1).

The long-term health of all societies depends on the health of the next generations, and the importance of taking a transgenerational and life-course approach to improving health of individuals and societies, beginning with the first moments of life—as this is when physical, mental and emotional health is established—and when personal, social and environmental attitudes and behaviours begin to form. This session explored the importance of early environmental ecology in determining lifelong health—including microbial diversity, nutrition, nature, social interactions during “critical windows” of development (Figure 8).

### 9.1. Across the Generations: A Traditional Perspective from the Longest Surviving Culture on Earth

**Sandra Eades** (Curtin University Kent St, Bentley, WA 6102, Australia).

No abstract available.

### 9.2. From Mother to Child: How, When and Why We Need to Optimise the Developing Microbiome

**Maria-CarmenCollado** (Institute of Agrochemistry and Food Technology-National Research Council (IATA-CSIC), 46980 Valencia, Spain).

Advances in the understanding of the host–microbes interactions suggest maternal microbiota plays a crucial role in infant health. Microbial colonization is essential for the immune system development and function. Intestinal microbes also affect other physiologic processes related to nutrition, metabolism, and intestinal homeostasis. Accumulating evidence suggests that the human microbial contact begins in utero and is then modulated by perinatal factors including mode of delivery and infant diet. Maternal microbiota therefore form the first and unique microbial inoculums, with microbial diversity increases and converges towards an adult-like microbiota by the end of the first years of life.

Alterations in this early microbial colonization (including delayed colonization, altered microbiota profiles and lower microbial diversity), are strong risk factors for the development of some diseases during life. Such alterations are mainly linked to C-section birth, antibiotic use and lack of breastfeeding practices. With a strong link between microbiota and risk of non-communicable diseases (NCDs), this imposes an increasing global burden that requires urgent action. It also underscores that conception, gestation and lactation are critical periods for human development where microbiota would play a role for future health.

There are several interventions targeted to modulate the microbiota. These include dietary counselling and also, probiotics, prebiotics and even antibiotics. To date, there has only been limited research to assess whether diet leads to changes in the microbiota and host interaction during gestation and thus, affect the specific strain microbial transference to the neonate both at birth and during lactation. At the same time, there is significant interest in research aimed at establishing the identity of specific microorganisms, microbial molecules and metabolites that contribute to the host’s physiology and health.

Taken together, these observations indicate that adequate nutritional and microbial environments during the perinatal period are key in promoting and supporting human health. Further research is required to determine how specific microbes, along with lifestyle, contribute to the maintenance of microbial and physiological equilibrium. This may reveal new opportunities to reduce the risk of non-communicable diseases by modulating the microbiota in early life. It therefore remains urgent to identify “how, when and why” we need to optimise the developing microbiome.

### 9.3. From Father to Child: Transgenerational Effects of Toxic Environmental Exposures in Artic Populations

**Janice Bailey ^1,2^** (^1^ Scientific Director, Fonds de Recherche du Québec en Nature et Technologies (FRQNT); ^2^ Professor, Department of Animal Sciences, Université Laval, Québec, QC G1V 0A6, Canada).

According to the World Bank (2016), the global fertility rate, defined as the number of births per woman, has declined markedly since the 1960s. This reduced fertility is often attributed to social issues related to family planning, such the successful use of effective contraception, couples making a financially based decision to have no or few children, or a shorter biological window of fertility when women choose to delay starting a family.

That environmental influences also play a role should not be ignored, and a Lancet Commission (2017) concluded that pollution is the largest environmental cause of disease and premature death in the world today. While it is well recognized that the maternal environment and lifestyle are important determinants of her children’s health, the influence of the father’s environment on his reproductive parameters and those of his subsequent generations has been overlooked. Indeed, according to a global study, sperm production has declined 60% since 1970, which could be a factor in declining fertility. Rapid biological shifts are usually considered to be environmental in nature and recent disturbing findings indicate that the effects of environmental exposures can be transmitted to future generations via fathers and his paternal lineage.

The overarching hypothesis of my team’s research is that exposure to environmental contaminants interferes with male fertility and the development and health of his future generations. To test the hypothesis, we created a multi-generation, outbred rat model during which the founder males are transiently exposed to an environmentally relevant mixture of persistent organic pollutants (POPs) contaminates the Arctic food chain and approximate levels in northern people. Our results demonstrate that sperm quality and pregnancy outcomes, including early embryo gene expression, are reduced across three, unexposed generations following a single developmental window of paternal exposure. We also showed that the sperm epigenome, specifically the profile of miRNAs and DNA methylation, is perturbed across three generations, providing insight into the molecular underpinnings of these phenotypes.

In conclusion, our research has demonstrated that (1) the environment influences male fertility across multiple, unexposed generations, and (2) the sperm epigenome exhibits marks of environmental insults. Taken together, our studies suggest that declining fertility could be related to POPs exposure of their previous generations through their fathers. The paternal environment affects the development of his descendants and must be considered for pregnancy outcomes and offspring health.

### 9.4. Early Life Exposome and Industrial Chemical Emissions

**Charlene Nielsen ^1^,Osornio Vargas ^1^ and Carl Amrhein ^2^** (^1^ University of Alberta, Edmonton, AB T6G 1C9, Canada; ^2^ Aga Khan University, Nairobi 74800, Kenya).

Industrial chemical emissions—because they are not routinely monitored—are an often-overlooked aspect of the early life exposome. Geographical Information Systems (GIS) helped us to map pollution sources, assign potential exposure, and analyse patterns of prenatal-related morbidities co-located with emissions from industrial facilities. Air pollution, irrespective of source, has a recognized association with adverse birth outcomes, such as low birth weight, small for gestational age, and preterm birth. We integrated the spatial locations and estimations of Environment and Climate Change Canada’s National Pollutant Release Inventory (NPRI) database into: (1) the identification of industrial chemical emissions co-located with adverse birth outcomes across Canada; (2) the development of a maternal ambient health hazard index for Alberta; and (3) the spatiotemporal modelling of hot spots in major Canadian metropolitan areas. We calculated spatial proxies of exposures to over two hundred industrial chemicals released to air and up to eighteen land-based hazards. For spatiotemporal analysis we estimated monthly dispersion of emissions by incorporating proximal weather station data. We assigned the modelled values to the six-character postal codes of the maternal residence at birth. Using correlation and logistic regression with pertinent covariates, we compared the resulting patterns. Over twenty of the chemicals revealed by our research are recognized or suspected developmental toxicants. We hope our research will contribute to effecting change in public perceptions and influence opinions leading to environmental health policies that will reduce the exposure of children to modifiable environmental hazards.

### 9.5. A Nudge from Evolution: Sex and Birth Order Influence How Mothers Eat and Offspring Grow

**Ralph Nanan ^1^, Sammuel Schäfer ^2^, Rosie Ribeiro ^1^, David Raubenheimer ^1^, Batool Nadim ^1^ and Anthony Liu ^1^** (^1^ The University of Sydney, Camperdown, NSW 2006, Australia; ^2^ Linköping University, Linköping, Sweden).

From an evolutionary perspective, reproductive success is proportionate to the number of offspring reared and their probability to reproduce. This implies that beyond the transmission of heritable characteristics to successive generations, biological fitness as a measure of offspring quality contributes to overall reproductive success. Early nutritional investment has been shown to influence the most important early determinants of offspring biological fitness, namely survival and growth. Higher birth weight leads to decreased infant morbidity and mortality. Furthermore, growth propensity appears to be an important determinant of lifetime reproductive fitness. This has been well described in animal models and might to some extent pertain to humans. We have explored relationships between offspring sex and birth order and maternal energy and macronutrient intake as well as the effects on the offspring weight development. Our results indicate that both gender and birth order drive distinct patterns of maternal total energy, macronutrient intake and influence offspring growth. To prevent metabolic pathologies over the lifespan these findings warrant further investigations towards targeted nutritional interventions during and after pregnancy, stratified for gender and birth order.

### 9.6. The Burden of Maternal Depression in Pregnancy: Implications for Psycho-Emotional and Physical Development of Next Generations

**Alkistis Skalkidou,Emma Bränn, Theodora Kunovac Kallak and Emma Fransson** (Uppsala University Women’s and Children’s Health, Akademiska sjukhuset, 751 85 Uppsala, Sweden).

Maternal peripartum depression (PPD) is associated not only with future maternal morbidity but also with sub-optimal child development and poor mental health. Despite the growing evidence of such an association, the mechanisms are still not fully understood. Within the population-based, prospective Biology, Affect, Stress, Imaging and Cognition (BASIC) cohort in Sweden, including 6472 pregnancies; mothers are followed from gestational week 17 throughout the first year after childbirth with web-questionnaires and biological sampling of blood, tissues, microbiota etc. We have investigated the biopsychosocial etiological processes involved in PPD and the associations of maternal depressive symptom trajectories with child development in the follow up cohort U-BIRTH, in which children are followed-up at 18 months, as well as at 6 and 11 years of age, regarding psycho-emotional and physical development.

In one study of 1093 children at 18 months, different time onset of maternal depression in the perinatal period showed distinct associations with child behavioural problems. While the effects of postpartum onset or persistent depression were mediated by maternal bonding to the infant, the effects of antenatal depression were an independent predictor of child behavioural problems. In another study, we investigated possible epigenetic mechanisms through DNA methylation using cord blood of 256 children born to mothers with or without antenatal depressive symptoms. In general, few epigenetic differences were identified, but analyses stratified for child sex showed that boys of depressed mothers, who also expressed higher internalized behavioural problems at 18 months, had lower DNA methylation at the MTH2 gene, which has been associated with brain development.

The cohorts have thus far generated 45 publications. Future studies will further investigate biological processes involved in PPD, through study of metabolomics and microbiome, the possibility of prediction of high-risk mothers, as well as the long-term health of children to mothers with peripartum depression.

### 9.7. Ecology of the Microbial Mother-Newborn Dyad

**Nelly Amenyogbe ^1^, Dennis Adu Gyasi ^2^, Yeetey Enuameh ^2^, Kwaku Poku Asante ^2^, Kaali Seyram ^2^, David Dosoo ^2^, Pinaki Panigrahi ^3^, Tobias Kollmann ^1^, WilliamMohn ^4^ and Seth Owuso-Agyei ^2^** (^1^ Telethon Kids Institute, Nedlands, WA 6009, Australia; ^2^ Kintampo Health Research Centre, Kintampo—North Municipality, Ghana, West Africa; ^3^ Georgetown University, Washington, DC 20057, USA; ^4^ University of British Columbia, Vancouver, BC V6T 1Z3, Canada).

From before birth, mothers begin nurturing their children and preparing them for a brave new world. The inheritance of specific microbes, or the microbiome, has garnered attention as a key avenue for mothers to shape the development of their children. Women’s microbiomes are also reshaped during pregnancy in a body site-dependent manner. The nature of these changes together with their consequences for her and her child’s health are only beginning to be realized. Here, we describe the ecology of both bacterial and fungal stool and breastmilk microbiomes of mothers, and the stool microbiomes of their children in the first post-partum month using molecular approaches. We observed massive differences in the bacterial stool microbiomes of women sampled in the first, compared to women sampled in the fourth post-partum week, including a lesser relative abundance Prevotella and greater abundance of Escherichia in the first, compared to fourth post-partum week. Differences of this scale were not observed for fungi. Further, while shared bacteria between mother’s breastmilk and child’s stool were readily identified, shared fungal species were sparse. Given the known ability of commensal fungi to influence immune development, the distinct pattern of their inheritance compared to that of bacteria is likely important to fully understand. Moreso, understanding the relationship between the surprisingly massive shifts in women’s bacterial microbiome around the time of birth to her and her child’s health must become a research priority.

### 9.8. Life in Our Gut: The Impact of Birth Mode, Diet and Social Interactions in Shaping the Microbiome during Early Life

**John Penders** (Department of Medical Microbiology, Maastricht UMC+ [University Medical Centre], 6202 AZ Maastricht, The Netherlands).

During infancy and early childhood, our intestinal microbiome establishes from the first inoculum to a complex and diverse ecosystem under the influence of a large number of perinatal environmental and maternal factors. Dispersal of maternal microbes is clearly limited in case of Caesarean section delivery, but also lack of social interactions with peers (e.g., with siblings or infants at daycare centres) or contact with pets have been shown to influence microbiome assembly during this critical age window. Diet plays an important role in shaping the infant microbiome both by dispersal of foodborne microorganisms (e.g., microbes in breast milk) but also by environmental selection (e.g., human milk oligosaccharides selecting for Bifidobacterium).

Perturbations in early life microbiome development as a result of our modern lifestyle has been linked to the increase in various non-communicable diseases including allergies and asthma, diabetes, inflammatory bowel diseases and overweight and obesity. These perturbations and the accompanying loss of keystone bacterial species (old friends) in the intestinal tract of the current generation of children and adolescents are driven by the increased use of antimicrobial agents, rising rates of C-section deliveries, decrease in breastfeeding, western diets (rich in animal fat and protein and low in dietary fibres), decreased family sizes, increased stress levels and our reduced contact with biodiversity in general and environmental microbes in particular.

The current COVID-19 pandemic has however resulted in an unprecedented abrupt shift in microbial exposures and stressors. Many countries across the world have experienced lock downs with playgroups, day cares and schools being closed, social interactions have been minimized and we refrain from physical contact beyond household members. Moreover, frequent hand washing is being strongly advocated and sales of disinfectants have risen more than 10-fold. Besides the aimed reduction of SARS-CoV-2 transmission, these measures have also resulted in reduced microbial transmission in general exemplified by the substantial decline in other respiratory as well as gastrointestinal viruses in many countries. The extent to which the current pandemic also affects the assembly of the commensal intestinal microbiome during infancy remains to be explored. On one hand, lack of social interactions might result in dispersal limitation of commensal microorganisms, but on the other hand, this might also result in reduced dispersal of antimicrobial resistant and opportunistic pathogenic bacteria. Additionally, the crowded forests, parcs and other green spaces during lockdowns might point to more exposure and connection to nature and thereby dispersal of environmental bacteria. Altogether, these challenging times also provide us with unique opportunities to study these forces on the development of the intestinal microbiome during early life.

### 9.9. Reduced Microbial Richness in Breast Milk Associates with Increased Risk of Immune Disease

Majda Dzidic ^1,2,3^, Alex Mira ^2^, Alejandro Artacho ^2^, Thomas R Abrahamsson ^4^, Maria Carmen Collado ^1,^*^,Ψ^ and Maria C Jenmalm ^3,Ψ^ (^1^ Institute of Agrochemistry and Food Technology (IATA-CSIC), Department of Biotechnology, Unit of Lactic Acid Bacteria and Probiotics, 46980 Valencia, Spain; ^2^ Department of Health and Genomics, Center for Advanced Research in Public Health, FISABIO, 46980 Valencia, Spain; and CIBER-ESP, Madrid; Spain; ^3^ Department of Biomedical and Clinical Sciences, Division of Inflammation and Infection, Linköping University, 581 83 Linköping, Sweden; ^4^ Department of Biomedical and Clinical Sciences and Department of Pediatrics, Linköping University, 581 83 Linköping, Sweden; ^Ψ^ Shared senior authors).

Background: Diverse microbial exposures play a crucial role for appropriate immune maturation during childhood. One important source of microbes for the infant is the breastmilk microbiota, transferred together with maternal IgA antibodies. In children developing allergies, we previously noted aberrant IgA responses to the gut microbiota already at 1 month of age, when the IgA antibodies are predominantly maternally derived in breastfed infants.

We aimed to determine the microbial composition and IgA-coating of bacteria in breastmilk in relation to allergy development in children participating in an intervention trial with pre- and postnatal *Lactobacillus reuteri* supplementation.

Methods: We characterized the bacterial recognition patterns by IgA in breastmilk samples collected one-month postpartum from 40 mothers whose children did or did not develop symptoms of allergy and asthma during the first seven years of life, using a combination of flow cytometric cell sorting and 16S rRNA gene sequencing.

Results: Children developing allergy received milk with a significantly lower bacterial richness, when compared to the milk fed to children that remained healthy, while the proportions of IgA-coated bacteria, the total bacterial load and the patterns of IgA-coating were similar in the two groups. Probiotic treatment influenced the breastmilk microbiota composition.

Conclusion: Reduced microbial richness in breast milk associates with increased risk of infant allergy development.

### 9.10. Human Breast Milk Bacterial Dysbiosis Is Associated with Lactose Fermentation and Poor Breastfeeding Outcomes

**Anna Ojo ^1^, Stefano Cacciatore ^2^, Shantelle Claassen-Weitz ^3^, Kilaza Mwaikono ^4^, Heather Zar ^5^, Dan Stein ^6^, Luiz Zerbini ^7^, MarkNicol ^8^ and Elloise du Toit ^3^** (^1^ University of Cape Town Medical School, Anzio Road, Observatory 7925, South Africa; ^2^ International Centre for Genetic Engineering and Biotechnology (ICGEB), University of Cape Town, 7700 Cape Town, South Africa; ^3^ Division of Medical Microbiology, Department of Pathology, Faculty of Health Sciences, Observatory 7925, University of Cape Town, Cape Town 7700, South Africa; ^4^ Department of Science and Laboratory Technology, Dar es Salaam Institute of Technology, P.O. Box 2958, Dar es Salaam 11000, Tanzani; ^5^ Department of Pediatrics and Child Health, Red Cross War Memorial Children’s Hospital, Rondebosch, Cape Town 7700, South Africa; ^6^ SA MRC Unit on Risk & Resilience in Mental Disorders, Dept of Psychiatry & Neuroscience Institute, Observatory 7925, University of Cape Town, Cape Town 7700, South Africa; ^7^ International Centre for Genetic Engineering and Biotechnology (ICGEB), University of Cape Town, 7700 Cape Town, South Africa; ^8^ School of Biomedical Sciences, Division of Infection and Immunity, The University of Western Australia, M504, Perth, WA 6009, Australia).

Human breast milk (HBM) is universally regarded as the optimal source of nutrition for the growing infant, however the impact of variation in the components of HBM on breastfeeding outcomes is not fully understood. In particular, the interaction between the HBM metabolome and microbiota has not been well studied. Here, we show that a dysbiotic HBM microbiota is associated with evidence of fermentation of lactose, low lactose concentration and poor breastfeeding outcomes. We identified a subset of South African lactating mothers with markedly reduced HBM lactose concentration. Mothers with low-lactose HBM breastfed exclusively for shorter periods and their infants grew less well during the period of exclusive breastfeeding. Metabolomic profiling of low-lactose HBM revealed an increase in metabolites associated with mixed acid fermentation, characteristic of microbial carbohydrate metabolism, and depletion of metabolites in the tricarboxylic acid cycle. We therefore explored the bacterial composition of HBM using 16S rRNA amplicon sequencing and showed marked differences in composition in low lactose samples, which were characterized by higher median relative abundance of Staphylococcus species, lower abundance of Streptococcus species and increased bacterial load. Our findings suggest that an aberrant HBM microbiota may result in fermentation of lactose and poor lactational outcomes. Since the HBM microbiota is potentially modifiable, if these findings are reproduced in other populations, they may identify opportunities to intervene to avert poor lactational outcomes.

### 9.11. A Prospective Study of Persistent Organic Pollutants and Body Mass Index Trajectories among Black Women

**Xiaoxia (Sasha) Han ^1^, Donna Baird ^2^, Quaker Harmon ^2^, Amelia Wesselink ^3^, Lauren Wise ^3^, Olivia Orta ^3^, Birgit Claus-Henn ^3^, Andreas Sjodin ^4^ and Ganesa Wegienka ^1^** (^1^ Henry Ford Health System, Detroit, MI 48202, USA; ^2^ National Institute of Environmental Health Sciences, Durham, NC 27709, USA; ^3^ Boston University School of Public Health, Boston, MA 02118, USA; ^4^ Centers for Disease Control and Prevention, Atlanta, GA 30333, USA).

This abstract not available; however, a recording of this presentation is available as part of the on-line proceedings.

### 9.12. Gut Microbiota Signature Based on Sex and the Presence of Siblings in 2-Year-Old Japanese Children

**Bahrul Fikri ^1^, Yumiko Nakanishi ^2^, Midori Yamamoto ^3^, Naoko Tachibana ^2^, Ayumi Ito ^2^, Ryohei Shibata ^2^, Tamotsu Kato ^2^, Taiji Nakano ^1^, Fumiya Yamaide ^1^, Kenichi Sakurai ^3^, Chisato Mori ^3,4^, Naoki Shimojo ^1^ and Hiroshi Ohno ^2^** (^1^ Department of Pediatrics, Graduate School of Medicine, Chiba University, Chiba 260-8670, Japan; ^2^ Laboratory for Intestinal Ecosystem, RIKEN Center for Integrative Medical Sciences, Yokohama City, Kanagawa 230-0045, Japan; ^3^ Center for Preventive Medical Sciences, Chiba University, Chiba 263-8522, Japan; ^4^ Department of Bioenvironmental Medicine, Graduate School of Medicine, Chiba University, Chiba 260-8670, Japan).

Background: Gut microbiota has been demonstrated to be associated with age and could be associated with the health and development of diseases such as allergy. However, less is still known about the profile of gut microbiota based on sex and the presence of siblings in early childhood. Here we studied the profile of gut microbiota based on sex and the presence of siblings in 2-year-old Japanese children.

Methods: This study is an adjunct study of the Japan Environment and Children’s Study (JECS). Faecal microbiome data were collected at 2 years of age in the Chiba regional centre. The Faecal microbiome was profiled by 16s rRNA gene sequencing. Microbiome analysis was performed using Quantitative Insight Into Microbial Ecology (QIIME) and Phyloseq in R package. Statistical analysis was performed by JMP13; *p* value was adjusted for multiple comparisons.

Results: Complete data were available for 267 children; there were 126 males and 141 females; 153 out of 267 had at least one elder sibling. In microbial diversity analysis, the genera Bifidobacterium was significantly lower abundance in male. In contrast, the genera Coprococcus, Lachnospira, and Sutterella were significantly higher abundance in males (Wilcoxon rank-sum test, *p* < 0.05). Meanwhile, in relation to the presence of elder siblings, we found that Parabacteroides was significantly overrepresented in children who had elder siblings compared to those who did not. Conversely, Dorea and Trabusiella were significantly underrepresented in children with elder siblings (Wilcoxon rank-sum test, *p* < 0.05).

Conclusions: The genera Bifidobacterium, Coprococcus, Lachnospira, and Sutterella could be the sex-associated gut microbiota in 2-year-old children. Parabacteroides, Dorea, and Trabulsiella could be gut microbiota determined in children who have elder siblings. These findings may be important for the pathomechanism of inflammatory diseases in children.

### 9.13. The Role of Early Life Gut Microbiota Maturation in Allergy Predisposition: Higher Risk of Sensitization among Asian–Canadian Children

**Hein M. Tun** (HKU-Pasteur Research Pole, School of Public Health, LKS Faculty of Medicine, University of Hong Kong, Pok Fu Lam, Hong Kong, China).

Increasing evidence supports the role of early life gut microbiota in developing atopic diseases, but ecological changes to gut microbiota during infancy in relation to food sensitization remain unclear. In this observational study, using 16S rRNA amplicon sequencing, we characterized the composition of 2844 faecal microbiota in 1422 Canadian full-term infants. Atopic sensitization outcomes were measured by skin prick tests at age 1 year and 3 years. The association between gut microbiota trajectories, based on longitudinal shifts in community clusters, and atopic sensitization outcomes at age 1 and 3 years was determined.

Four identified developmental trajectories of gut microbiota were shaped by birth mode and varying by ethnicity. The trajectory with persistently low Bacteroides abundance throughout infancy increased the risk of sensitization to food allergens, particularly peanut at age 3 years by three-fold (adjusted OR, 2.82; 95% CI, 1.13–7.01); a much higher likelihood for peanut sensitization was found if infants with this trajectory were born to Asian mothers (adjusted OR, 7.87; 95% CI, 2.75–22.55). Importantly, this trajectory of depleted Bacteroides abundance mediated the association between Asian ethnicity and food sensitization. Our study is the first to show a mediation role for infant gut microbiota in ethnicity-associated development of food sensitization.

Further reading (details now published):

Tun HM, Peng Y, Chen B, et al. Ethnicity associations with food sensitization are mediated by gut microbiota development in the first year of life. Gastroenterology. 2021 Mar 16:S0016-5085(21)00523-0. Epub ahead of print. PMID: 33741316.

### 9.14. Ancestral Paternal Exposure to Arctic Pollutants Impairs Placental and Foetal Developmental Outcomes without Protective Effect from Folic Acid Supplementation

**Phanie L. Charest ^1^, Maryse Lessard ^1^, Pauline Herst ^1^, Pauline Navarro ^1^, Mathieu Dalvai ^1^, Marie-OdileBenoit-Biancamano ^2^ and Janice Bailey ^1^** (^1^ Université Laval, Rue de l’Université, Québec, QC G1V 0A6, Canada; ^2^ Université de Montréal, Montréal, QC J2S 2M2, Canada).

Inuit people have more adverse pregnancy outcomes and shorter life expectancies. Because of their traditional diet, they are highly contaminated by Persistent Organic Pollutants (POPs), known to induce negative health effects. We hypothesize that folic acid (FA) supplementation attenuates developmental disorders in foetuses and placentas associated with prenatal paternal exposure to POPs over multiple generations.

We used a four-generation rat model, in which F0 founder females were divided into four treatment groups (n = 8) and gavaged with corn oil or an environmentally relevant Arctic POPs mixture before mating and until parturition. F0 females were fed either a base-FA diet (2 mg/kg), or a supplemented-FA diet (FAS; 6 mg/kg). Twelve F1 males/treatment were mated to untreated females to produce F2 rats, etc. until F4. Histopathological examination of the foetuses and placentas were performed at Gestational Day 19.5. The cut-off value for significance is *p* ≤ 0.05.

The foetal:placental weight ratio (FW:PW) was reduced by POPs*FAS interaction in F1–F2. In contrast, FW:PW was increased due to POPs in F3 and by both POPs and FAS in F4. Similarly, the length of the limb long bones was reduced due to POPs in F1–F2, whereas it was increased in F4. Placental histomorphometry was impaired by POPs and FAS in F1–F2, although no differences were present in F3–F4. These results suggest inadequate placental efficiency and possible compensatory mechanisms.

Prenatal paternal POPs exposure impairs development over multiple generations. FAS, however, may not represent an ideal solution to counteract the consequences of POPs. Multigenerational transmission of the paternal environment is apparent and may occur via placental disruption. Achieving our objectives will broaden current understanding of the toxicological impacts of the environment on human health and the developmental origins of disease.

## 10. Healthier Beginnings: Early Life Interventions. Making Change Early in Life (Developmental Origins—Part 2)

**Figure 9:**
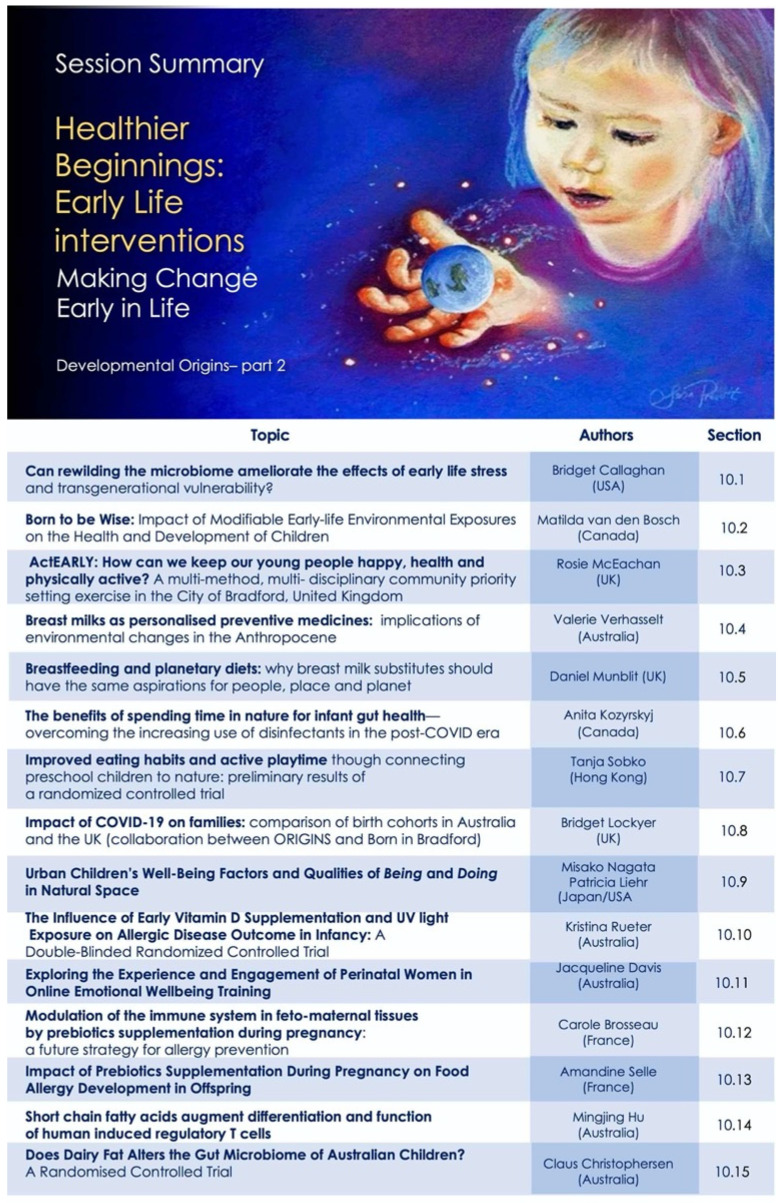
Healthier Beginnings: Early Life interventions: Overview of topics and speakers in the session on developmental ecology (Part 2).

Chronic inflammatory diseases, collectively known as non-communicable diseases (NCDs), now pose the greatest threat to human health globally. Many of these conditions have their origins in early life, and there is a pressing need to understand how the modern environment is contributing to this unsustainable health burden. There are mounting concerns that this new generation will have a shorter life expectancy than their parents, particularly in disadvantaged populations. Early interventions will ultimately be the only way of curtailing this and are an essential aspect of improving global health. This session explores different aspects of improving early life ecology (Figure 9).

### 10.1. Can Rewilding the Microbiome Ameliorate the Effects of Early Life Stress and Transgenerational Vulnerability?

**Bridget Callaghan** (Brain and Body Lab, University of California-Los Angeles, Los Angeles, CA 90095, USA).

No abstract available.

### 10.2. Born to Be Wise: Impact of Modifiable Early Life Environmental Exposures on the Health and Development of Children

**Matilda van den Bosch ^1^, Brauer Michael ^1^, Rick Burnett ^2^, Hugh Davies ^1^, Zoe Davis ^1^, Martin Guhn ^1^, Ingrid Jarvis ^1^, Lorien Nesbitt ^1^, Tim Oberlander^1^, Hind Sbihi ^1^, Jason Su ^2^ and Michael Jerrett ^3^** (^1^ The University of British Columbia, Vancouver, BC V6T 1Z4, Canada; ^2^ Health Canada, Vancouver, BC K1A 0K9, Canada, University of California, Berkeley, CA 94720, USA; ^3^ University of California, Los Angeles, CA 90095, USA).

Deficiencies in childhood development is a major global issue and inequalities are large. The influence of environmental exposures on childhood development is currently insufficiently explored. The Born to be Wise project analyses the impact of modifiable early life environmental exposures on different dimensions of childhood development.

Our cohort contains approximately 34,000 children who have completed an early development test at the age of five. Land use regression models of air pollution and spatially defined noise models will be linked to geocoded data on early development to investigate harmful effects. The potentially beneficial effect on early development of early life exposure to natural environments, will also be explored. The project will analyse overall and age specific impact, including variability depending on cumulative exposure by assigning time-weighted exposure estimates and by studying subsamples who have changed residence and exposure. Potentially moderating effects of natural environments on air pollution or noise exposures will be studied by mediation analyses. A matched case-control design will be applied to study moderating effects of natural environments on the association between low socioeconomic status and early development. The main statistical approach will be mixed effects models, adjusting for multilevel random effects of nested data. All models will be adjusted for a priori selected confounders.

The Born to be Wise project is relevant to a key set of contemporary challenges related to rapidly changing living environments and harmful exposures that may influence early childhood development. In many areas, these exposures are ubiquitous, thus affecting large populations with the potential for major impacts on population health. Results from the project will provide empirical evidence on the impact of environmental exposures on early development and may ultimately inform effective policies and interventions to support healthy urban planning for vulnerable populations, particularly children in socioeconomically deprived areas.

### 10.3. ActEARLY: How Can We Keep Our Young People Happy, Health and Physically Active? A Multi-Method, Multi-Disciplinary Community Priority Setting Exercise in the City of Bradford, United Kingdom

**Rosie McEachan, Aamnah Rahman and Chris Cartwright** (Bradford Institute for Health Research, Bradford Teaching Hospitals NHS Foundation Trust, UK).

Economic, physical, built, cultural, learning, social and service environments have a profound effect on lifelong health. However, policy thinking about health research is dominated by the “biomedical model” which promotes medicalisation and an emphasis on diagnosis and treatment at the expense of prevention. Prevention research has tended to focus on “downstream” interventions that rely on individual behaviour change, frequently increasing inequalities. Preventive strategies often focus on isolated leverage points and are scattered across different settings. Born in Bradford is part of a new ActEarly research consortium aims to create City Collaboratory testbeds to support the identification, implementation and evaluation of upstream interventions within a whole system city setting to improve children’s health.

Community co-production is vital to ensure that preventive strategies are acceptable and feasible to implement which in turn will increase their effectiveness. A multi-method priority setting exercise led by a community steering group was conducted with communities in 2019 to inform the focus of the consortium. Adapting a James Lind Alliance approach to research priority setting we conducted a survey with 1000 community members and over 20 community events asking people what they felt was important to keep children “happy and healthy”. We found that people identified healthy eating, physical activity and healthy lifestyles as priorities for keeping children healthy most often; whilst they identified mental health, socialising and nurturing environments most often as priorities for keeping children happy. Communities’ priorities reflected both individual determinants (e.g., education and attitudes) and structural determinants (e.g., access, environment) of health as important. The findings are being used to co-design prevention strategies to improve the health and happiness of families in Bradford and London (Tower Hamlets), UK. For more information see: https://actearly.org.uk/ accessed on 1 August 2021.

### 10.4. How Changes in the Anthropocene May Influence the Role of Breast Milk as Child’s Best Personalised Medicine?

**Valerie Verhasselt** (University of Western Australia, and Telethon Kids Institute, Nedlands, WA 6009, Australia).

Breast milk contains thousands of bioactive compounds, which have the potential to influence infant immune health. The composition of breast milk is highly dependent on maternal environment, and we propose to consider breast milk as a mini-exposome dedicated to preparing the child to his future exposome. We suggest this key function of breast milk is hampered by new practices of breastfeeding and by extreme changes of breast milk composition in the Anthropocene. Here, we will review some evidence of this working hypothesis.

The personalized medicine afforded by breastfeeding is best illustrated by the immune defences that are provided to the breastfed newborn. A mother exposed to respiratory or enteric pathogens will produce antibodies that will protect her from infection. Importantly, those antibodies will also be secreted in breastmilk through the gut–respiratory–breast axis and provide passive immunity to the newborn against pathogens that are specific to the environment where the newborn is raised. Our recent data also highlight that pathogens’ antigen shedding in maternal milk may be a way to naturally vaccinate the offspring. In the era of globalization, newborns are now receiving human milk from milk bank where breast milk was collected at distant places and may not have the appropriate composition for the child.

A striking observation is that breast milk is not protecting from allergic disease, and this is not expected for the child’s physiological food that is endowed with multiple immune modulatory properties. To understand this conundrum, we hypothesis that extreme change in maternal exposome may have affected the anti-allergenic properties of breast milk. Supporting this hypothesis, our data both in rodents and in human cohorts showed that the presence of house dust mite allergen in human milk (found in about 50% of milk samples) was associated with increased risk of allergy in offspring. House dust mite have been living on our planet for million years and have probably always been present in maternal milk. To address the hypothesis that maternal exposome has changed the milk composition and its capacity to inhibit allergic priming in offspring, we are now exploring the influence of maternal diet on milk anti-allergenic properties. Within the ORIGINS birth cohort, we aim to reveal whether maternal intake of prebiotics can restore such a profile.

Knowing that more than 80% of the children will be receiving human milk, there is a critical need to provide mothers with recommendations that increase the chance of child healthy development. The understanding of how the multiple breast milk bioactives will influence immune outcomes in offspring is fundamental to instruct evidence-based maternal interventions that will contribute to increase child health through breastfeeding.

### 10.5. Breastfeeding and Planetary Diets: Why Breast Milk Substitutes Should Have the Same Aspirations for People, Place and Planet

**Daniel Munblit** (Imperial College London, St Mary’s Hospital, Praed St, London W2 1NY, UK).

No abstract available.

### 10.6. The Benefits of Spending Time in Nature for Infant Gut Health—Overcoming the Increasing Use of Disinfectants in the Post-COVID Era

**Anita Kozyrskyj** (Department of Pediatrics, Faculty of Medicine & Dentistry, Alberta, Edmonton Clinic Health Academy, Edmonton, AB T6G 1C9, Canada).

Natural environments (e.g., native vegetation) and biodiversity, through the influence of environmental microbes, may be beneficial for human commensal gut microbiota, especially for its development during infancy. The nature on gut health story in Dr. Kozyrskyj’s presentation was told in two parts with evidence from the CHILD Cohort Study: (i) depletion of environmental microbes and hence, infant gut microbiota in the home through greater use of disinfectant cleaning products, and (ii) proximity to natural environments in urban centres as an intervention to restore depleted gut microbes in young infants.

Main findings from the first study showed that increasing frequency of home disinfectant use successively increased the chance of an infant having high levels of Lachnospiraceae in their gut microbiota at 3–4 months of age and becoming overweight at age 3. Taking the statistical analysis one step further by conducting a mediation test, higher levels of the Lachnospiraceae bacteria were found to be responsible for the association between disinfectant use and child overweight.

The second study was based on data linkage between the unique Urban Primary Land and Vegetation Inventory (uPLVI) for the city of Edmonton and 355 infants in the CHILD birth cohort, infant exposure to natural environments (yes/no) was determined within 500 m of their home residence. Gut microbiota composition and diversity at age 4 months was assessed in infant faecal samples. Adjusted for covariates, we observed a reduced odds of high microbial alpha-diversity in the gut of infants exposed to any natural environment (Simpson index = 0.63 (0.41, 0.98)). In stratified analyses, these associations remained only among infants not breastfed or living with household pets.

When doubly stratifying by these variables, the reduced likelihood of high alpha-diversity was present only among infants who were not breastfed and lived with household pets (9% of the study population, Simpson diversity index = 0.11 (0.02, 0.66)). Differences in beta-diversity was also seen (*p* = 0.04) with proximity to a nature space in not breastfed and pets-exposed infants. No associations were observed among infants who were fully formula-fed but without pets at home. When families and their pets had close access to a natural environment, Verrucomicrobiales colonization was reduced in the gut microbiota of formula-fed infants, the abundance of Clostridiales was depleted, whereas the abundance of Enterobacteriales was enriched.

Our double-stratified results indicate that proximity to a natural environment plus pet ownership has the capacity to alter the gut microbiota of formula-fed infants. 

### 10.7. Improved Eating Habits and Active Playtime though Connecting Preschool Children to Nature: Preliminary Results of a Randomized Controlled Trial

**Tanja Sobko** (University of Hong Kong, Pok Fu Lam, Hong Kong, China).

The urgency to develop and test novel and more diverse lifestyle intervention programs for pre-school children is obvious. We feel our study breaks new ground in terms of demonstrating the additional benefits accrued by strategically integrating short, simple, creative, and interactive natural world engagements for young children and their caregivers participating in lifestyle intervention programs aimed at promoting healthier eating and nature practices. The Connectedness to Nature (CN) component of the researched Play & Grow programme shows to amplify the effects of some of the standardised nutrition education of the intervention. We further explored new direct links regarding feeding and eating habits and show that the Play & Grow intervention positively increased caregivers and children CN, which improved children eating behaviours. This indicates a higher autonomy in children’s eating behaviours if they are exposed to nature. Further, the methodology described could help researchers/practitioners to better understand how connectedness to nature affects child health when it comes to feeding, eating and wellbeing. We therefore believe that our paper would be of particular interest in the context of health behaviours, health promotion in early childhood and early environmental education. Future research should include and further test the CN module in promotion of healthy lifestyles in pre-schoolers, especially its possible long-term effect.

Suggested Reading: T. Sobko, G. Brown and W.H.G Cheng. Does Connectedness to Nature improve the eating behaviours of pre-schoolers? Emerging evidence from the Play & Grow randomised controlled trial in Hong Kong. Appetite.2020 Nov 1;154:104781. 

### 10.8. Impact of COVID-19 on Families: Comparison of Birth Cohorts in Australia and the United Kingdom (Collaboration between ORIGINS and Born in Bradford)

**Bridget Lockyer ^1^, Lisa Gibson ^2,3,4^, Josie Dickerson ^1^, Charlotte Endacott ^1^, Sally Bridges ^1^, Rosemary McEachan ^1^, Kate Pickett ^7^, Sarah Whalan ^2^, Natasha Bear ^8^, Desiree Silva ^2,3,4,5^, Susan Prescott ^2,3,4,5^ and Jacqueline Davis ^2,3,5^** (^1^ Bradford Institute for Health Research, Bradford Teaching Hospitals NHS Foundation Trust, UK; ^2^ Telethon Kids Institute, 15 Hospital Avenue, Nedlands, WA 6009, Australia; ^3^ The University of Western Australia, Perth, Australia; ^4^ Joondalup Health Campus, Perth, Australia; ^5^ Edith Cowan University, Perth, Australia; ^6^ Curtin University, Perth, Australia; ^7^ Department of Health Science, University of York, UK; ^8^ Institute for Health Research, Notre Dame University, Perth, WA 6160, Australia).

Few studies have documented the impact of the COVID-19 pandemic on the emotional wellbeing, needs and concerns of the general population. Free text questions were administered to participants in the ORIGINS (Australia) and Born in Bradford (UK) cohort studies to collect qualitative information on worries, concerns and enjoyable experiences during the pandemic. A total of 903 (400 for ORIGINS and 503 for BIB) participants completed the two surveys during April 2020. Despite varying in geography, levels of socio-economic disadvantage and the situational context during the pandemic, respondents from both cohorts reported similar worries and challenges during the lockdown period including employment/finances, health anxiety, mental health and social isolation, caring for children and child development. Families across the globe experienced both positive and negative immediate impacts of COVID-19. Population-based data can be used to inform the development of support services, public health campaigns and universal interventions to assist families in future health crises.

### 10.9. Urban Children’s Wellbeing Factors and Qualities of Being and Doing in Natural Space

**Misako Nagata ^1^and Patricia Liehr ^2^** (^1^ Department of Health Science and Nursing, Graduate School of Medicine, The University of Tokyo, 7-3-1 Hongo Bunkyo-City, Tokyo, 113-0033, Japan; ^2^ Christine E. Lynn College of Nursing, Florida Atlantic University, 777 Glades Road, Boca Raton, FL 33431, USA).

In the presentation, I discuss my mixed methods research study about urban children’s wellbeing through nature immersion—being and doing in natural space—from the perspective of Nursing. This research came from my lifelong inquiry: everyday phenomena around us can be observed in order or pattern, and this would be the conditions of natural law. Certain phenomena can occur if certain conditions meet. Then, healing could occur when nature around us meets with certain conditions for healing. Florence Nightingale (1820–1910) said that what nursing has to do was to arrange the environments of the patient in the best condition for nature to act upon him. Given a hint from her statement, I developed a concept called Nature Immersion. Nature Immersion is defined as connecting with earthy materials to generate healing force from within embodied as personal emergence. Healing emerges when nature meets the person with certain conditions. This assumption launched the research on wellbeing conditions for urban children who were distant from nature.

Battery Urban Farm, which is located at the south tip of Manhattan, New York, is surrounded with green and blue space with a community garden. The farm was chosen to explore parental reports of wellbeing conditions of urban children. Both the quantitative data (N = 174) and the qualitative data (N = 15) were collected. The research led a new model for the conditions of urban children’s wellbeing. There were space–time factors and parental appreciation of their children’s being and doing in urban nature. The results indicated the wellbeing conditions were those with nature–child immersion: higher parental appreciation, higher frequency of visits, short-lived but expansive nature exposure, learning and caring hubs, and integration that creates oasis. Environmental conditions altogether, compared to parental socioeconomic status, had a significant impact (*p* < 0.001).

From the results, it may have meant about what Nightingale said, “Health is not only to be well, but to be able to use every power we have to use”. Her statement is linked to a sort of natural law where health, healing, and wellbeing are expressed as emergent processes of rejuvenating, remembering, and reconnecting the whole system of oneself—body, mind, spirit, culture, and environment. This natural law is not fixated but flexible, for as urban children choose to be and do in nature, they are connecting their whole system, creating potential for wellbeing.

### 10.10. The Influence of Early Vitamin D Supplementation and UV Light Exposure on Allergic Disease Outcome in Infancy: A Double-Blinded Randomized Controlled Trial

**Kristina Rueter ^1,2^, Anderson P. Jones ^3^, Aris Siafarikas ^1,2,3,4^, Paola Chivers ^4,5^, Susan L. Prescott ^1,2,6^ and Debra J. Palmer ^1,3^** (^1^ School of Medicine, The University of Western Australia, 35 Stirling Highway, Crawley, WA, 6009, Australia; ^2^ Perth Children’s Hospital, 15 Hospital Avenue, Nedlands, WA 6009, Australia; ^3^ Telethon Kids Institute, University of Western Australia, 15 Hospital Avenue, Nedlands, WA 6009, Australia; ^4^ School of Medical and Health Science, Edith Cowan University, Perth, WA 6027, Australia; ^5^ Institute for Health Research, The University of Notre Dame Australia, Perth, WA 6160, Australia; ^6^ The ORIGINS Project, Telethon Kids Institute and Division of Paediatrics, University of Western Australia, 15 Hospital Avenue, Nedlands, WA 6009, Australia).

Background/Aims: The epidemic of allergic disease is a public health crisis particularly in children in developed countries. Changing human behaviour has led to less sunlight exposure and associated predisposition to vitamin D deficiency. Vitamin D is known to have immunomodulatory functions and low levels of vitamin D have been linked to allergies in children. However, randomised controlled trials (RCTs) on the effect of vitamin D supplementation during infancy as an allergy prevention strategy are lacking.

Method: We investigated the influence of oral vitamin D supplementation for the first 6 months of life in high-risk infants (n = 195). Uniquely, sunlight exposure was measured by a UV-dosimeter. Infant blood samples were taken to determine relationships between oral vitamin D supplementation and ultraviolet (UV) light exposure with blood 25(OH)D concentration and immune cell function responses to allergens. At 6 months, 1 and 2.5 years of age infants were examined for allergic disease outcomes.

Results: At 1 year of age results showed no difference in eczema (*p* = 0.17), eczema severity (*p* = 1.0), wheeze (*p* = 0.13) and food allergy (*p* = 0.98) incidence or sensitisation (*p* = 0.24) between the vitamin D supplemented and placebo groups. This finding was consistent with our 6 months outcomes (eczema (*p* = 0.68), eczema severity (0.82); wheeze (*p* = 0.79)). However, infants with eczema in the first 6 months had significantly less UV light exposure from 0 to 3 months than children without eczema (*p* = 0.023). UV light exposure was also inversely correlated with IL-2, GMCSF and eotaxin production to Toll-like receptor ligands.

Conclusions: Initial findings of this RCT reveal that early vitamin D supplementation had no influence on allergic disease outcome in infancy. However, UV light exposure in the first 3 months was independently associated with immunomodulatory effects and appeared to reduce the risk of eczema. Further follow up data are in preparation and will be presented at the conference.

### 10.11. Exploring the Experience and Engagement of Perinatal Women in Online Emotional Wellbeing Training

**Jacqueline Davis ^1,2^,Susan Prescott ^1,2^, Amy Finlay-Jones ^1^, Jeneva Ohan ^2^ and Desiree Silva ^1,2,3^** (^1^ Telethon Kids Instiute, Nedlands, WA 6009, Australia; ^2^ University of Western Australia, Nedlands, WA 6009, Australia; ^3^ Joondalup Health Campus, Joondalu, WA 6027, Australia).

Background: The perinatal (antenatal and postnatal) period can be a time of increased psychological distress. Up to 9% of women experience depression during pregnancy and up to 16% suffer from postnatal depression. Maternal psychological distress (stress, anxiety and depression) in the early postpartum period can disrupt mothers’ wellbeing, functional ability, interpersonal relationships, including those with the infant. Subjective wellbeing is crucial for positive mental health and wellbeing. Positive mental health could be a buffer during times of psychological distress, such as that experienced in the perinatal period which effects the mother and potentially the developing child. Cost-effective interventions that support lasting positive mental health, while also preventing symptoms of negative mental health, are of critical importance for public health. While there are evidence-based interventions that can be applied during the perinatal period, there is little known about enduring engagement and sustained practices.

Aim: This PhD project’s aim is to understand the engagement and experience of women undertaking perinatal minimal contact wellbeing interventions that build positive, sustained emotional assets.

Methods: A series of studies will be undertaken within the ORIGINS Project, an existing longitudinal pregnancy cohort study in Western Australia. These include a pilot Randomised Controlled Trial (RCT), “Mums Minds Matter” that will explore women’s experience and engagement of online mindfulness and self-compassion training using a mixed methods design. Women will be followed up at various timepoints, post-intervention. A second study will assess willingness to engage by type of available online wellbeing program. Third, “Community Wellbeing during COVID-19” will explore pregnant women’s emotional wellbeing information and support needs during the pandemic period. Additionally, a systematic review will explore the extant literature on engagement and retention in digital mental health and wellbeing programs by perinatal women.

Status: Ethics approval has been provided, pending confirmation of the third study. Data collection is underway.

### 10.12. Modulation of the Immune System in Feto-Maternal Tissues by Prebiotics Supplementation during Pregnancy: A Future Strategy for Allergy Prevention

**Carole Brosseau** (French National Institute for Agriculture, Food, and Environment [INRAE], Centre de Recherche Angers-Nantes 44000, France).

Allergies are multifactorial diseases related to the dysfunction of the microbiota, epithelial barriers and the immune system leading to a failure in the establishment of immune tolerance. Pregnancy represents an optimal window of intervention in the regulation of the allergic process by modulating the immune and microbial systems of the foetus. Prebiotics can modulate the immune system, the microbiota and the intestinal barrier.

A preclinical study carried out in our laboratory shows that prebiotics supplementation during pregnancy and lactation reduces the development of food allergy in offspring. The aim of this study was to understand the immunological processes of prebiotics administered during pregnancy only on foetal and maternal tissues. Pregnant mice received a standard diet or a diet enriched with prebiotics (GOS/inulin). After 18 days of gestation, the frequency of the different lymphoid and myeloid cell populations was determined in the different gestational (decidua, placenta, uterus), maternal (spleen) and foetal (intestine, blood) tissues. The effect of prebiotics on the frequency of hematopoietic stem cells from mother and foetus femur was also determined. 

Supplementation with prebiotics during gestation increases significantly the frequency of IL-10-secreting regulatory B lymphocytes in the placenta compared to mice on a standard diet. They are also found in the intestine and bone marrow of the foetus. The rate of regulatory T cells is also significantly increased in the placenta of mice supplemented with prebiotics compared to control. Prebiotics have no effect on the frequency of dendritic cells nor on the homeostasis of hematopoietic stem cells. 

In conclusion, prebiotic supplementation during pregnancy leads to the establishment of a tolerogenic environment which could protect the foetus against future allergies.

### 10.13. Impact of Prebiotics Supplementation during Pregnancy on Food Allergy Development in Offspring

**Amandine Selle, Carole Brosseau, Angéline Duval and Marie Bodinier** (French National Institute for Agriculture, Food, and Environment (INRAE), Centre de Recherche Angers-Nantes 44000, France).

Allergies are multifactorial diseases related to the dysfunction of three biological actors: the microbiota, the epithelial barriers and the immune system. These alterations lead to a defect in the establishment of immune tolerance. Compelling evidence for the early role of gut microbiota dysbiosis and barrier integrity with allergies is emerging. In this context, pregnancy represents an optimal window of intervention in the regulation of the allergic process through a modulation of the immune and microbial systems, which makes it a promising avenue for the prevention of allergy. Prebiotics are able to act on the immune system, the microbiota and the intestinal barrier. The aim of our study is to assess the effect of prebiotic supplementation during pregnancy on the development of food allergy in offspring. To do this, mice received a diet enriched in prebiotics (GOS/inulin) during pregnancy. Then, food allergy to wheat was induced in their offspring by two intraperitoneally sensitization and one oral challenge with the allergen (gliadins). Subsequently, we analysed clinical symptoms and immunological and physiological biomarkers of allergy. In the context of prebiotic supplementation, we observed in offspring: 1) a tendency to decrease allergic symptoms; 2) no decrease of allergic markers (TH2, IgE); 3) a tendency to increase tolerance biomarkers; 4) a decrease in the degradation of the intestinal barrier in the presence of food allergies. These results show that prebiotics could induce tolerogenic environment on the offspring when they are used during pregnancy, but this strategy is not enough to reduce the allergy.

### 10.14. Short Chain Fatty Acids Augment Differentiation and Function of Human Induced Regulatory T Cells

**Mingjing Hu ^1^, Bilal Alashkar Alhamwe ^2^, Brigitte Santner-Nanan ^1^, Sarah Miethe ^2^, Hani Harb ^3^, Harald Renz ^2^, Daniel P. Potaczek ^2^ and Ralph Nanan ^1^** (^1^ Charles Perkins Centre Nepean, Sydney Medical School Nepean, The University of Sydney, Sydney, NSW 2747, Australia; ^2^ Institute of Laboratory Medicine, Member of the German Center for Lung Research (DZL), and the Universities of Giessen and Marburg Lung Center (UGMLC), Philipps-University Marburg, Germany; ^3^ Division of Immunology, Boston Children’s Hospital, Harvard Medical School, Boston, MA 02115, USA).

Regulatory T cells (Tregs) control immune system activity and inhibit inflammation. While in mice short-chain fatty acids (SCFAs) are known to be essential regulators of naturally occurring and in vitro induced Tregs (iTregs), data on their contribution to the development of human iTregs are sparse, with no report of the successful SCFA-augmented in vitro generation of fully functional human iTregs. Likewise, markers undoubtedly defining human iTregs are missing. Here, we aimed to generate fully functional human iTregs in vitro using protocols involving SCFAs and to characterize the underlying mechanism. Besides, our target was to identify the potential phenotypic markers best characterizing human iTregs. Naïve non-Treg CD4^+^ cells were isolated from peripheral blood of 16 healthy adults and cord blood of 12 healthy term newborns. Cells were subjected to differentiation towards iTregs using a transforming growth factor β (TGF-β)-based protocol, with or without SCAFAs, acetate, butyrate, or propionate. Thereafter, they were subjected, with or without preceding restimulation, to flow cytometric phenotyping or suppression assay. During differentiation, cells were collected for chromatin-immunoprecipitation-based analysis of histone acetylation. Enrichment of the TGF-β-based protocol with butyrate or propionate potentiated the in vitro differentiation of human naïve CD4^+^ non-Tregs towards iTregs and augmented the suppressive capacity of the latter. These were at least partly underlain by the effects of SCFAs on histone acetylation levels in differentiating cells. GITR, ICOS, CD39, PD-1, and PD-L1 proved to be potential markers of human iTregs. Our results might boost further development of Treg-based therapies against autoimmune and chronic inflammatory disorders.

### 10.15. Does Dairy Fat Alter the Gut Microbiome of Australian Children? A Randomised Controlled Trial

**Claus Christophersen ^1,2^, Kane E. Deering ^2^, Analise Nicholl ^2^, Amanda Devine ^2^, Rose Lines ^2^, Johnny Lo ^2^, Mary C. Boyce ^2^ and Therese A. O’Sullivan ^2^** (^1^ Curtin University, Bentley, WA 6102, Australia; ^2^ Edith Cowan University, Joondalup, WA 6027, Australia).

There is a paucity of knowledge on the effect of dairy fat on the gut microbiota in young children. This study, therefore, examines the impact of whole-fat dairy intake, compared to reduced-fat dairy intake, on the paediatric gut microbiome.

Forty-nine healthy Australian children who were regular consumers of whole-fat dairy were recruited for a 12-week double-blind randomised clinical trial (ANZCTR: 12616001642471). Participants were distributed using block randomisation into either a whole-fat dairy group (WFDG) or reduced-fat dairy group (RFDG). Participants were provided with ad libitum dairy products based on their habitual intake. Three-day weighed food records, physical measurements, blood and stool samples were collected at baseline and post-intervention.

Forty-nine participants (mean (SD) age = 5.26 (0.88) years, female n = 23) were randomised (WFDG = 25; RFDG = 24); 46 children completed the intervention. The two groups consumed similar amounts of dairy, however, participants in the WFDG consumed significantly more fat (grams) (pFalseDiscoveryRate < 0.001) and saturated fat (pFDR < 0.001) per day. All participants met the adherence guideline (1 serving) of dairy (300 mg calcium) per day with an overall median serve of 2.01. The microbial analysis revealed that there was no overall significant change in richness, -diversity (Shannon diversity) or -diversity in either group at the phylum or genus level. While there was a significant increase in the relative abundance of Actinobacteria (pFDR = 0.012) and Subdoligranulum (pFDR = 0.048) in children allocated to the RFDG group. In the WFDG, relative abundance of Bacteroides increased significantly (pFDR = 0.036) and Massiliomicrobiota decreased significantly (pFDR = 0.005). The relationship between the faecal microbiota, metabolites and the consumed diet is still being investigated.

Modifying dairy fat intake in young Australian children may alter the relative abundance of specific bacteria; however, it is unlikely to significantly change alpha- or beta-diversity.

## 11. From the Grassroots: Change through Communities and Networks

**Figure 10:**
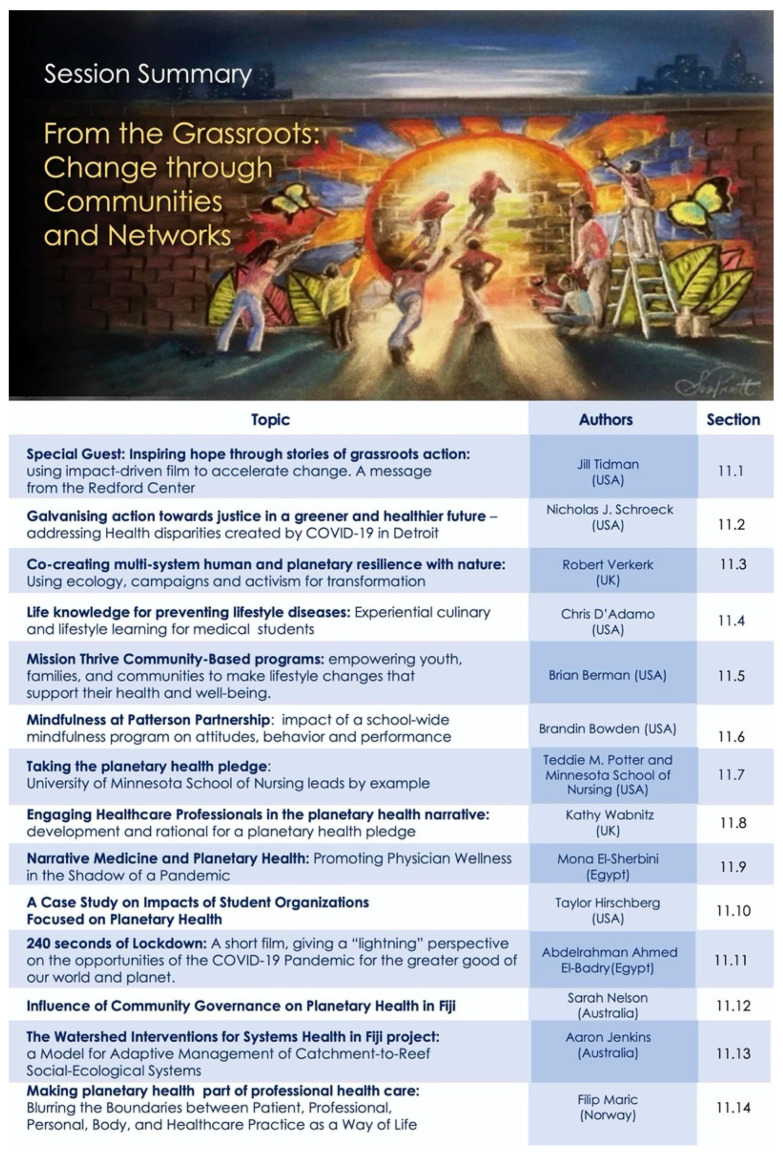
Making change through communities and networks: Overview of topics and speakers in the session on grassroots engagement.

Meaningful change increasingly depends on grassroots strategies that improve the health of people and places—and ultimately our planet. These strategies empower communities, engender optimism, and encourage collaboration among different sectors of government, industry, science, and the public and increase opportunities for people to engage in change personally and collectively. This session explores the importance of empowering and connecting communities—that people everywhere can have more effect than they realize by telling their stories of change, no matter how small they seem, to empower others—for a contagion of positive change (Figure 10). Connecting all these many stories of change is how we build large scale change. This requires imagination, creativity, courage and inspiration—and shared stories to help create just that!

### 11.1. Special Guest: Inspiring Hope through Stories of Grassroots Action: Using Impact-Driven Film to Accelerate Change. A Message from the Redford Center

**Jill Tidman** (Executive Director. The Redford Center, The Presidio, San Francisco, CA 94129, USA).

This is a message of hope from The Redford Center and includes three short films telling stories of change in number of communities, including a message from co-founder Robert Redford, *Uniontown Votes*, *Hunger for Change* and *Closing the Gap*. This recording (including the films) is available through our online proceedings [12].

### 11.2. Galvanising Action towards Justice in a Greener and Healthier Future—Addressing Health Disparities Created by COVID-19 in Detroit

**Nicholas J. Schroeck** (University of Detroit Mercy School of Law, 651 East Jefferson Ave., Detroit, MI 48226, USA).

This presentation examines drinking water regulations under the United States Safe Drinking Water Act pertaining to bacteria and the type of public notice required when a disease outbreak related to public water supply occurs. Then, the focus shifts to lessons learned from the failure to provide public notice of the Legionnaires’ disease outbreak in Flint and Genesee County, Michigan during the Flint water crisis. Additionally, I analyse the recent proposed changes to the federal Safe Drinking Water Act, Lead & Copper Rule. I provide a brief overview of the Lead & Copper Rule and the Environmental Protection Agency’s proposed changes to the Rule. Finally, I identify remaining gaps in the Lead & Copper Rule, particularly in relation to environmental justice communities, and compare and contrast the Safe Drinking Water Act with regulations in other countries.

### 11.3. Co-Creating Multi-System Human and Planetary Resilience with Nature: Using Ecology, Campaigns and Activism for Transformation

**Robert Verkerk** (Science Unit, Alliance for Natural Health International, Chilworth, Surrey, Chilworth, Surrey GU4 8NS, UK).

Planetary, ecosystem and human resilience are degrading rapidly because ecosystem services utilised by humans are exceeding their natural capacity for sustainability. Increasing recognition of the linkage between the challenges facing human health and those facing the environment offer unique opportunities for the resolution of both crises. Such solutions require a widescale recognition of the ecological interactions both within and outside the human body within the context of a systems approach. This contrasts with the siloed, reductionist approach that has been central to rapid technological development in industrialised countries that has also been, directly or indirectly, central to the loss of integrity and resilience of human and natural systems.

Any transition towards sustainable health systems requires the development of proactive, eco-centric health systems that are focused on creating, maintaining or regenerating adaptive capacity, health and resilience throughout the lifespan of individuals in communities. Such an approach needs to extend well beyond the scope of existing, largely top-down public health support. Successful implementation of such a proactive, salutogenesis-based systems would greatly reduce net disease burdens on existing, largely reactive, disease-centric, “health care” systems, while also reducing net costs and productivity losses incurred by society.

Two essential prerequisites are: (a) a universal language of communication between citizens, health professionals, health authorities, businesses, educational establishments and other organisations and institutions; and (b) a multi-disciplinary scientific framework that extends beyond the current and limited scope of the medical sciences. Extensive consultation with experts, health professionals from diverse backgrounds, communities and industry has concluded with a consensus view that the language should be based around physiological, psychological and social function, while systems ecology provides the appropriate multi-disciplinary framework. Based on this approach, a two-tiered, bottom-up model for community-based, sustainable health systems has been created by the Alliance for Natural Health International.

The first tier of the model allows for self-assessment, guided assessment or biomedical assessment of resilience of individuals via 12 domains of an individual’s “ecological terrain”. Within communities, individuals are guided by health and fitness professionals (“health guides”) in ways that optimise function while minimising stressors that limit the adaptive and transformative capacity across all domains. Both citizens and their health guides would subscribe to shared values, goals and priorities, including use of the common language around system function. The second tier of the model includes 10 hallmarks for health system sustainability that must be fulfilled to ensure compliance by health guides and citizens alike. The model lends itself to extensive digitisation and would contribute substantially to the democratisation of health and care systems.

Real-world testing of the community-based sustainable heath system model is now urgently required, particularly in the wake of COVID-19 which has further widened health inequalities and emphasised the importance of high quality, measurable, self-care in communities. Such seismic changes to the predominantly reactive nature of existing healthcare systems will not be triggered without compelling pressure and powerful activism by influential individuals, communities, organisations and forward-thinking, purpose-driven companies.

### 11.4. Life Knowledge for Preventing Lifestyle Diseases: Experiential Culinary and Lifestyle Learning for Medical Students

**Chris D’Adamo** (Department of Family and Community Medicine, Center for Integrative Medicine, University of Maryland School of Medicine, Baltimore, MD 21201, USA; The Institute for Integrative Health, 1407 Fleet Street, Baltimore, MD 21231, USA).

Nutrition education for medical students has generally been limited in both curriculum time and relevance to patient care. This paucity of nutrition training carries through into residency and ultimately clinical practice. Thus, physicians are often left with limited knowledge and tools to help patients eat healthier diets known to reduce the risk of chronic disease. “Culinary medicine” education has become increasingly popular in medical schools across the United States to help fill this important gap in training. Medical students electing to participate in experiential culinary medicine education have demonstrated improvements in nutrition knowledge, skills, and attitudes for both personal and patient care. While promising, most culinary medicine education is currently only offered on an elective basis to relatively small cohorts of medical students.

Following a series of successful elective pilot sessions, culinary medicine training was incorporated into the core medical student curriculum and was provided to all first-year medical students at the University of Maryland School of Medicine in the 2019–2020 academic year. The training was held at the community-based teaching kitchen of the Institute for Integrative Health in Baltimore, Maryland. The three-hour training included a combination of: (1) evidence-based nutrition lecture supporting a unifying “nutrient density” approach to numerous popular dietary approaches (Mediterranean, low-carbohydrate, low-fat, palaeolithic, plant-based) while offering practical strategies to help overcome personal and patient barriers to healthy eating, (2) cooking simple recipes based upon tenets of the lecture, and (3) eating the meal together and discussing the applications of the training to personal and patient care. A mixed-methods outcomes analysis was conducted and statistically significant improvements were noted in mean scores of all nutrition knowledge, skills, attitudes and patient counselling outcomes that were assessed.

Themes of being better prepared to address barriers to healthy eating in patient care, familiarity with multiple evidence-based popular diets to help offer choices to patients in how to eat healthier, and ability to make practical healthy eating behaviour changes in the medical students’ own lives were noted in qualitative analysis.

In light of the success of the culinary medicine training for medical students, an interprofessional culinary medicine course has been developed and is currently being offered at the Institute for Integrative Health to medical, nursing, dentistry, social work, pharmacy, and law students at the University of Maryland, Baltimore graduate campus. The course has been similarly well received and plans for expansion of this experiential training are underway.

### 11.5. Mission Thrive Community-Based Programs: Empowering Youth, Families, and Communities to Make Lifestyle Changes That Support Their Health and Wellbeing

**Brian Berman ^1,2^, Brandin Bowden ^1^ and Christopher R. D’Adamo ^1,2^** (^1^ The Institute for Integrative Health, 1407 Fleet Street, Baltimore, MD 21231, USA; ^2^ Department of Family and Community Medicine, Center for Integrative Medicine, University of Maryland School of Medicine, Baltimore, MD 21201, USA).

This was a video presentation based on the work of the Mission Thrive Summer (MTS) program, that was developed to address the physical inactivity, poor nutrition, and chronic stress, which threaten the health of African American youth in urban environments. The program was targeting in the summer period when conditions often worsen with diminished access to healthy foods and safe venues for physical activity. MTS was an integrative health intervention developed as a public-private partnership—based at an urban farm and adjacent school in a low-income community in Baltimore, Maryland. The intervention included farming, nutrition education, cooking, physical activity, yoga, mindfulness, and employment. Mixed-methods outcomes evaluation was conducted. Quantitative measures included accelerometery and self-reported health behaviours, using the Child and Adolescent Mindfulness Measure, Perceived Stress Scale, Physical Activity Questionnaire for Adolescents (PAQA), CDC Youth Risk Behavior Survey, and Block Kids Food Screener (BKFS). Participants experienced statistically significant improvements in self-reported physical activity (PAQA) and dietary habits (BKFS). Surveys did not detect changes in stress or mindfulness. Qualitative data demonstrated new knowledge and skills, increased self-efficacy, health behaviour change, and program enjoyment. These findings show that MTS was feasible among African American high school students in Baltimore. Mixed-methods outcomes evaluation provided preliminary evidence of health behaviour change during the summer and at follow-up.

Further reading: B. Pierce et al. A Summer Health Program for African-American High School Students in Baltimore, Maryland: Community Partnership for Integrative Health. Explore (NY). 2017; 13 (3): 186–197.

### 11.6. Mindfulness at Patterson Partnership: Impact of a school-wide mindfulness program on attitudes, behaviour and performance

**Brandin Bowden ^1^,Brian Berman ^1,2^ and Christopher R. D’Adamo ^1,2^** (^1^ The Institute for Integrative Health, 1407 Fleet Street, Baltimore, MD 21231, USA; ^2^ Department of Family and Community Medicine, Center for Integrative Medicine, University of Maryland School of Medicine, Baltimore, MD 21201, USA).

The Mindfulness at Patterson Partnership (MAPP), an initiative of the Institute for Integrative Health, evaluated the Holistic Life Foundation’s Mindful Moment program at Patterson High School in Baltimore, Maryland. The 15 min mindfulness practice included seated yoga, breathwork and silent reflection. The University of Maryland School of Medicine’s Center for Integrative Medicine helped identify a combination of school-wide and student-level outcomes to explore the research question: “Is implementing a mindfulness program in an entire urban high school feasible and effective”. The research team concluded this primarily intercom-based delivery of a school-wide mindfulness intervention feasible, with limited effectiveness.

The feedback and lessons learned during MAPP highlighted some of the nuances of collecting outcomes in a real-world setting. The Institute’s Building Bridges Defining Metrics Forum convened a variety of stakeholders to amplify similar lessons and inspire collaborative solutions. These rich discussions informed a set of guiding principles for creating and evaluating community-based programs that impact youth health in Baltimore City.

Further reading: Building Bridges, Defining Metrics: Envisioning an integrative approach to community-based programs to improve the health of urban and disadvantaged youth. Forum Report, 3–4 February 2016, Baltimore: found at: https://tiih.org/files/9614/6921/8738/tiih_2016BBDMreport_final_lowres.pdf accessed on 1 August 2021.

### 11.7. Taking the Planetary Health Pledge: University of Minnesota School of Nursing Leads by Example

**Teddie M. Potter** and the students and staff at Minnesota School of Nursing provide a video message “taking the pledge” (School of Nursing University of Minnesota 308 Harvard St. SE 6-189B Weaver Densford Hall Minneapolis, MN 55455, USA).

The 2020 planetary health pledge published in the Lancet was recited:

“I solemnly pledge to dedicate my life to the service of humanity, and to the protection of natural systems on which human health depends. The health of people, their communities, and the planet will be my first consideration and I will maintain the utmost respect for human life, as well as reverence for the diversity of life on Earth”.

“I will practise my profession with conscience and dignity and in accordance with good practice, taking into account planetary health values and principles. To do no harm, I will respect the autonomy and dignity of all persons in adopting an approach to maintaining and creating health which focuses on prevention of harm to people and planet. I will respect and honour the trust that is placed in me and leverage this trust to promote knowledge, values, and behaviours that support the health of humans and the planet. I will actively strive to understand the impact that direct, unconscious, and structural bias may have on my patients, communities, and the planet, and for cultural self-awareness in my duty to serve. I will advocate for equity and justice by actively addressing environmental, social, and structural determinants of health while protecting the natural systems that underpin a viable planet for future generations. I will acknowledge and respect diverse sources of knowledge and knowing regarding individual, community, and planetary health such as from Indigenous traditional knowledge systems while challenging attempts at spreading disinformation that can undermine planetary health. I will share and expand my knowledge for the benefit of society and the planet; I will also actively promote transdisciplinary, inclusive action to achieve individual, community, and planetary health. I will attend to my own health, wellbeing, and abilities in order to provide care and serve the community to the highest standards. I will strive to be a role model for my patients and society by embodying planetary health principles in my own life, acknowledging that this requires maintaining the vitality of our common home. I will not use my knowledge to violate human rights and civil liberties, even under threat; recognising that the human right to health necessitates maintaining planetary health”.

“I make these promises solemnly, freely, and upon my honour. By taking this pledge, I am committing to a vision of personal, community, and planetary health that will enable the diversity of life on our planet to thrive now and in the future”.

Full reference: K. Wabniz et al. A pledge for planetary health to unite health professionals in the Anthropocene. The Lancet 2020; 396 (10261): 1471–1473.

### 11.8. Engaging Healthcare Professionals in the Planetary Health Narrative: Development and Rational for a Planetary Health Pledge

**Kathy Wabnitz** (Department of Public Health and Primary Care, The Primary Care Unit, University of Cambridge CB2 0SR, UK).

In 1948, the Declaration of Geneva was passed by the recently founded World Medical Association, aiming to promote ethical principles underpinning medical practice worldwide. It was grounded in the thinking and practice of the Greek physician Hippocrates and aims to serve as a universally valid set of principles to which doctors should adhere. Reciting the Declaration with her fellow graduates at the Medical Faculty of Tübingen, Germany in 2018, was a significant moment for Katharina Wabnitz, the lead author of a recently published commentary in The Lancet: “A pledge for planetary health to unite health professionals in the Anthropocene”. This experience sparked the idea of formulating a new pledge, which is broader in scope and audience and engages not only doctors but all health professionals in protecting the health of people and planet. It includes principles such as intergenerational justice and a new interpretation of what it means to Do no harm in the Anthropocene. Health professionals are among the most trusted members of society. They can play a crucial role in igniting and becoming agents themselves of individual and systemic transformative changes. These changes include a shift from the current predominant focus on acute care to promoting and protecting the health of people and the state of natural systems that underpin the latter through a life-course and intergenerational approach to health and a focus on primary prevention. The authors of this pledge emphasize its suggestive character, published to be used in graduation ceremonies and other events but also to spark further discussion and development of adapted versions to fit local and institutional purposes. We hope that this contribution will be a powerful tool to spark individual and collective action for planetary health.

Further reading: K. Wabnitz et al. A pledge for planetary health to unite health professionals in the Anthropocene. The Lancet 2020; 396(10261):1471–1473

### 11.9. Narrative Medicine and Planetary Health: Promoting Physician Wellness in the Shadow of a Pandemic

**Mona El-Sherbini** (Department of Parasitology, Cairo University, Gisa 12613, Egypt).

In the face of mental health challenges secondary to the global pandemic of COVID-19 and the larger malaise narratives that operates around them. It is important for physicians to pay attention to their baseline stress levels, their emotions, and the effect their work is having on them. A magnitude of sharp career demands, strains in the work settings, with barriers to help-seeking such as time constrains and concerns about confidentiality, inevitably may aggravate mental health consequences such as depression, anxiety and post- traumatic stress disorder among physicians and frontline healthcare providers, impacting the wellbeing and productivity of billions. 

Early interventions may mitigate the mental health impact on its providers. The core tenets of “Narrative medicine” have emerged to recognize vulnerability (in the self and others), cultivate emotional equilibrium and foster self-awareness, placing the human subject at the centre, as a harmonic system continuously interacting with himself and his environment. Planetary Health coordinates interdependent sustainable vitality of all natural and human ecosystems. In synchronizing Planetary Health principles with Narrative practices in the form of mindfulness and reflection, we may have an opportunity in making physician mental wellbeing revitalized with just a few seconds focus micro-practices or strategies to manage the emotional aspects of stress and leverage positive psychology. Physicians are encouraged to find their own methods for cultivating mindfulness in their daily lives, applying mindfulness micro-practices as to STOP (Stop, Take a breath, Observe, Proceed) before seeing new patients or starting new activity, at bedtime or before getting up. Through physician’s self- regulated attention and capacity for living in resonance with the outside world, becoming conscious of thoughts, feelings and possible prejudices. Linking narrative medicine to planetary health approaches may help physicians achieve mindset equilibrium for concomitant personal and professional growth as crucial aspects in sustaining the quality of care and wellbeing.

### 11.10. A Case Study on Impacts of Student Organizations Focused on Planetary Health

**Taylor Hirschberg and Christopher Lowry** (University of Colorado-Boulder, Boulder, CO 80309, USA).

As stated in the Canmore Declaration, “Planetary health is vital to and inextricably interlinked with high-level wellness...and humankind must advocate for planetary health”. There is a need for dialogue in pre-health care educational settings on the relationships between planetary health and human health. Consequently, we are developing a student organization at the University of Colorado Boulder to address this topic. This ethnographic case study is being used to develop an understanding of changes in student development and quality of life after joining. In Spring of 2020, a student organization was created to bring awareness, inter-institutional collaboration, and advocacy. A pedagogical approach aligned with social events and community service will be the initial structure of the organization with the intent to broaden the understanding of planetary health and to promote self-identification of what planetary health means to its members. We will administer three surveys at baseline and again at the end of each semester for a one-year period. First, to assess the impact of the initial programming on student perspectives of planetary health, an online qualitative survey will be given to members. This survey will determine if the student: (1) felt engaged and connected to other students; (2) modified patterns of behaviour around protection of the environment; (3) explored new career opportunities; (4) changed previously held understanding of planetary health; and (5) responded with increased interest in the field of planetary health. We will use the Connectedness to Nature Scale (CNS) to determine if engagement in the program affected connectedness to nature, which has been shown to impact quality of life. Furthermore, to test the hypothesis that a sense of nature connectedness may impact the quality of life of the participants, we will use the 36-item short form survey (SF-36), a validated survey that assess physical, mental, and overall health.

### 11.11. 240 s of Lockdown: A Short Film, Giving a “Lightning” Perspective on the Opportunities of the COVID-19 Pandemic for the Greater Good of Our World and Planet

**Abdelrahman Ahmed El-Badr** (Cairo University, Gisa 12613, Egypt).

“The consequences of a pandemic could be modulated by the positive narrative we make of it”. Building upon this notion, the featured 4 min film with a birds-eye view beyond individual physical lockdown or isolation to deeper narratives portraying the qualitative similarities and demarcations between today’s COVID-19 pandemic and the 1918 Spanish Flu pandemic, highlighting and conceptualizing “Wisdom of Pandemics” as opportunities during adversity for the greater good of our world and planet.

Throughout history, grand narratives have weaved together humanities and natural sciences shaping the future of civilizations. Thus, offering insights into the origins and impacts of pandemics on human experience and the ability to give voices to the complex malaise we must heal, changing the way we live on Earth: from ecology to social coexistence from a holistic approach.

The 1918 pandemic emerged during World War One with great social, economic and mega-planetary upheaval. COVID-19 pandemic and global warming were mentioned as a few examples of the fragility and unsustainability of human activities and ecosystems.

Overwhelmed by the observable details, we may have turned a blind eye to the positive human relationships represented by the courage, faith and solidarity of healthcare workers, and the hints of planetary revival seen in the return of wildlife to places where they were long lost.

In conclusion, planet Earth will “go on with or without us”, and we must delve deeper into the hidden ecological webs in addition to the physical measures taken during the pandemic, with humanity in our line of sight.

### 11.12. Influence of Community Governance on Planetary Health in Fiji

**Sarah Nelson Joel Negin, Aaron Jenkins and Seye Abimbola** (University of Sydney, Camperdown, NSW 2006, Australia).

Background: Fijian village committees play an important role in managing ecological ecosystem, human health and planetary broader societal outcomes within communities, and the wider catchment. The actions and practices of individual communities and their committees impact the health of the humans and their ecosystems. Understanding these actions and practices provides understanding how communities manage their natural resources.

Methods: A mixed method approach was used. A quantitative survey was conducted on rules, structure and governance of community committees for planetary health. Descriptive analysis was conducted. Semi-structured interviews and focus group discussions were conducted in communities to understand structure and management of water resources from leadership and water committees. Thematic analysis was conducted using NVIVO.

Results: Community governance and committee practices, varied amongst committees: some committees had written rules, and organisational practices whereas others had unwritten rules with ad hoc practices. Without meetings, issues are not raised and discussed, and planetary health practices are unable to change. Regular meetings and greater community involvement in decision-making practices were wanted by community members. Women’s roles were found to vary among committees and the community members felt a stronger role was required by women. Committee and community behaviours were found to impact the state of ecosystem, especially the state water.

Conclusion: Current community governance and committee practices do not always consider planetary health and are typically considered in a reactive nature. Stronger governance and practices were wanted by the community. Creating appropriate understanding of community planetary health actions, can help create a healthier ecosystem, reducing disease, improving farming techniques, land use practices and improving water quality.

### 11.13. The Watershed Interventions for Systems Health in Fiji Project: A Model for Adaptive Management of Catchment-to-Reef Social-Ecological Systems

**Aaron Jenkins ^1,2^, Stacy Jupiter ^3^, Pierre Horwitz ^2^, Jacqueline Thomas ^1^, Rachel Devi ^1^, Samuela Lagataki ^3^, Sangeeta Manghubai ^3^ and Joel Negin ^1^** (^1^ Wildlife Conservation Society; ^2^ University of Sydney, Camperdown, NSW 2006; ^3^ Edith Cowan University, Bentley, WA 6027, Australia).

Research from Fiji during the past decade has linked watershed modification to the spread of waterborne bacterial disease, such as typhoid, in human populations, as well as degradation in downstream freshwater and coral reef communities. Since declines in downstream human health and ecological condition and share several of the same ultimate drivers (e.g., forest loss, amount of area of high erosion risk, density of creek crossings), well-designed and coordinated watershed interventions should enable achievement of multiple objectives across sectors at reduced expense.

The Watershed Interventions for Systems Health in Fiji (WISH Fiji) project provides a model case for transdisciplinary cooperation to: evaluate initial catchment conditions; co-design management interventions with Fijian landowners and industry; and monitor downstream responses in indicators of public health and ecosystem wellbeing through iterative cycles of adaptive management. We present on the WISH Fiji project design, initial diagnostic analysis of catchment and reef condition, and the participatory process of co-designing management interventions to address factors affecting whole-of-system health at nested scales. Data informing management choice selection include indicators of: human behavioural characteristics; water and sanitation infrastructure condition; water and soil contamination; catchment land use; coral reef condition; fisheries catch; and community governance.

This proof-of-concept initiative, which showcases collaboration from academia, government, civil society, and public and private philanthropy, has the potential to scale nationally through: (1) predictive models that can identify targeted areas for investment to improve water quality and reduce disease burden; and (2) innovative platforms of blended finance. We share lessons to enable proliferation of integrated watershed management that moves beyond project-based implementation to mainstreamed practice for enduring coral reef and human health and wellbeing.

### 11.14. Making Planetary Health Part of Professional Health Care: Blurring the Boundaries between Patient, Professional, Personal, Body, and Healthcare Practice as a Way of Life

**Filip Maric** (Environmental Physiotherapy Association (EPA); University of Trondhein, Høgskoleringen 1, 7491 Trondheim, Norway)

Coming to planetary health as a physiotherapist, I explore the potential of an alternative understanding of physiotherapy, both to ground my own planetary healthcare practice, and as a contribution to this burgeoning healthcare movement. This alternative conception of physiotherapy draws on two sources of inspiration:

First is the notion of a “Way of Life” that can be traced through ancient traditions across the Globe, including Buddhism, Daoism, Greek and Roman philosophy, and others. The strengths of this notion are the way in which it highlights the synergistic embodiment of practice and philosophy and, therefore, places the focus on the question of how we live, before the question of what we do professionally. The embodied synergy between philosophy and practice and the ensuing shift in focus supports the undoing of the boundary between personal and professional practice and, importantly, also between healthcare recipient and healthcare professional. The undoing of these boundaries is critical at a time that requires broad collaboration across all sectors of society and “the work of the 7.5 billion people currently alive” (Lancet Countdown 2019 Report), and thus a time when private, professional and collective health and action can no longer be thought about and “treated” separately.

The second source of inspiration is a radical, etymological play on “physiotherapy” as a service for all and everything that grows. I trace my arrival at this terminological dis/juncture with my profession through a critique of its hitherto understanding of self and other by drawing on the ethics of 20th century philosopher Emmanuel Levinas. Particularly the resultant view of subjectivity further underscores the way in which physiotherapy is an embodied practice that precedes and undermines the personal–patient–professional boundaries, but therewith also common notions of climate/health activism and interventionism.

## 12. Taking It Forward: Elements and Actions for Meaningful Change?

**Figure 11:**
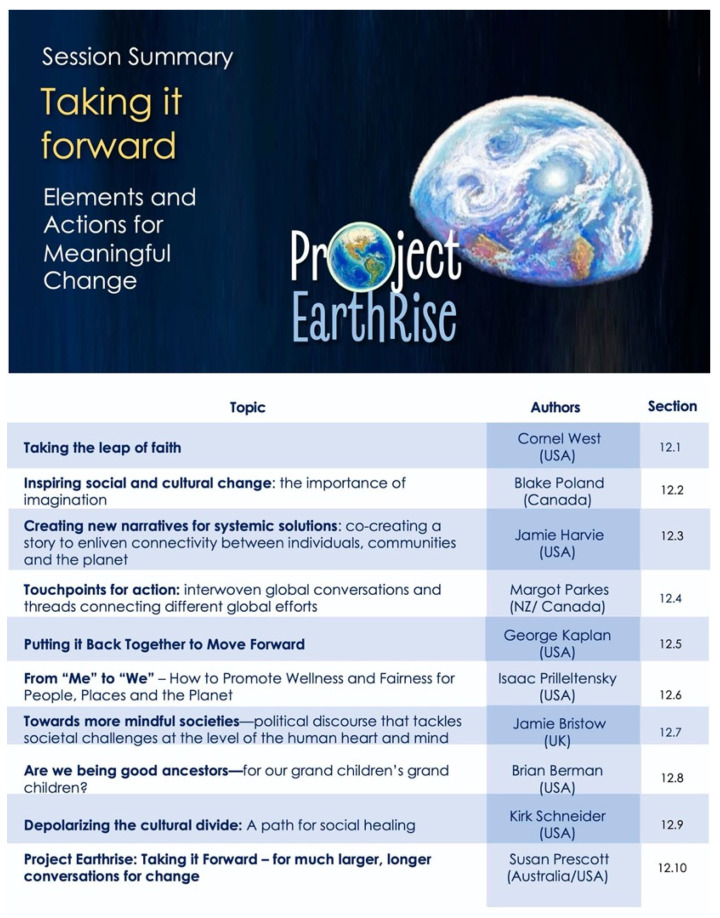
Elements and Actions for Meaningful Change: Overview of topics and speakers in the session considering the “next steps” for *Project Earthrise*.

The *Project Earthrise* agenda is about changing the stories that have created many failing systems, by promoting integrated approaches in the broadest possible sense—recognising connectivity across all scales, from deep biology to the spiritual purpose that inspires us towards discovery—both self-discovery and the way that we can improve opportunities and experience for all people, and the health of our places and planet, always remembering the far-reaching transgenerational implications. This session (Figure 11) explored the potential directions of these evolving narratives, and the next steps.

### 12.1. Taking the Leap of Faith

**Cornel West** (Professor of the Practice of Public Philosophy, Harvard Divinity School, Cambridge, MA 02138, USA; Professor Emeritus at Princeton University, Princeton, NJ 08544, USA).

No abstract available: a recording of Dr. Cornel West’s inspirational words is available for viewing through our online proceedings [12].

### 12.2. Inspiring Social and Cultural Change: The Importance of Imagination

**Blake Poland** (Dalla Lana School of Public Health, University of Toronto, Toronto, ON M5T 3M7, Canada)

The contemporary sustainability crisis is arguably, at its core, a crisis of (limited) imagination. As Fredric Jameson famously put it, “it has become easier to imagine the end of the world than the end of capitalism”. TINA (there is no alternative) reigns supreme. Oscar Wilde reminds us that a map of the world that does not include Utopia is not worth even glancing at. Surely Martin Luther King would concur.

It is indeed time to dream a better dream than the so-called “American Dream” that left many behind and many strung out on Prozac. In addition to stopping further destruction, we are invited to be known by what we stand for, and not just what we stand against. We must animate ourselves with dreams worth reaching for. By Paul Hawken’s reckoning, thousands if not hundreds of thousands of groups are doing just that.

Sometimes named as “prefigurative”, social movements have been experimenting with modes of organization, social relationships, and behaviours that make real a desired future in the current moment and context. The Transition movement is but one of these. This “positive turn” is evident all around us, including in academia, spanning work on appreciative inquiry, empowerment evaluation, capability theory, positive deviance, resilience, hope studies, Buddhist notions of “basic goodness”, quantum social theory, Indigenous cultural renewal and resurgence, gay (and LGBTQi) pride, fat-positive, nonviolent communication, and more.

These movements reflect the understanding that energy flows where attention goes, and that a deficit orientation and constant diet of negativity and fighting against is wearing and sometimes counterproductive, and that something magical happens when we collectively focus on peace, our universal humanity, and what Charles Eisenstein refers to as “the more beautiful world our hearts know is possible”. What if, in so doing, we call what we most fervently desire into being through a kind of morphic resonance, in much the same way as quantum science shows how electrons phase-shift from wave to particle by the mere act of being observed? A whole new kind of “sacred activism”, to borrow Andrew Harvey’s term, becomes possible; one that could inspire and unleash untold collective energies for “The Great Turning” (from an industrial growth paradigm to a life-centred approach predicated on a capacity to listen to and work with nature, flourishing through interdependence, “inter-being”, and sacred reciprocity).

Dreams are encoded in the stories we tell ourselves about who we are and where we are headed, individually and collectively. Daring to dream of a better future means telling different stories that enliven and animate us to match the size of the challenges we face with the boldness of our vision for a better world and our faith in the power of a collective change of heart and united collective action that can change worlds in the face of even the most recalcitrant vested interests.

### 12.3. Creating New Narratives for Systemic Solutions: Co-Creating a Story to Enliven Connectivity between Individuals, Communities and the Planet

**Jamie Harvie** (Institute for a Sustainable Future, Duluth, MN 55802, USA).

For the last three to four centuries humanity has experimented with a mechanistic mental model of the world. The model assumes certainty and is dependent on a belief in a hierarchy of human value and a hierarchy of all life. Yet, we dwell on a planet, fully alive, self-healing, hurtling through the cosmos with all life, sensing, probing and communicating. There is much more we do not see than what we do see. The world as a machine is at odds with the reality of the complexity of life. This incongruity results in planetary inflammation, racial inequality, species collapse, ecological grief, and more.

We are in the midst of a great transition in which humanity is reawakening to the interconnectedness of all life, of non-duality. We are being called to step into the unknown, the mystery and to the original story, ancient, first told by our shared ancestors that comes from the universe itself. Through models and approaches, this story is being written around us using language of resilience, mutuality, interconnectedness, holism, co-operation, reciprocity, shared ownership, self -organization, agency and the commons.

Our collective job is to call these new examples out, invest in them, share them, and seek others out. We have to be curious, and we have to listen to our language such that we do not reinforce old patterns. Are we advancing power over or power within? It will require us to go to and trust our hearts and our sensing. To acknowledge that as economists understand and complexity science explains, we are not rational beings, not predictable machines. That we feel. We need elders, to call on ancestors and need ritual to help us move through birth, adolescence, life and death. This is not a call for a technological fix but rather a shift in mindset and a reconnection to one’s heart, to one another, to spirit.

### 12.4. Touchpoints for Action: Interwoven Global Conversations and Threads Connecting Different Global Efforts

**Margot W. Parkes** (University of Northern British Columbia, Prince George, BC V2N4Z9, Canada; University of Otago, Dunedin 9016, Aotearoa/New Zealand).

Learning how to be “better together” is an essential element of our collective efforts to address a healthy, just and ecologically viable future on our shared planetary home. Drawing on insights from eco-social elders, and recent decades of (re)learning about interrelated efforts, this presentation reflects on how we respond to connections, confluence and convergence among complementary global efforts, and the opportunity to turn these into “touchpoints for action”. Emphasis is given to the interweaving global conversations that have been emerging across overlapping fields such as ecohealth, one health, planetary health and other allied efforts. Identifying the threads connecting these different efforts is enhanced by an ecological and systems orientation, that encourages reflection and awareness of what we are connecting, and important related questions of why? where? when? how? for and with whom? who benefits? from these connections. We are challenged to ask so what? in relation to respectfully enhancing collective co-benefits, for equity and ecosystems? As we look toward “next generation” options for cultivating connections and conversations, this ecological orientation also asks what we need to do to design for “emergence”?

Emergence arises from the ways in which the parts of a system influence one another, expressing consequences and leading to outcomes that would not have occurred if the components had not been interacting. With many different global efforts, there is a risk of dispersal and competition, rather than intentionally designing for co-benefits and “emergence” that address the imperatives of people, place and planet. “Both/and” thinking encourages us to value both attention to different parts, and connections among these parts. Indigenous leadership approaches also offer guidance, as exemplified by the word “Rangatira”, which in Māori means “chief” and is also closely linked to “raranga”, the act of weaving. Leadership that weaves people together to achieve their common goal, requires a shift in emphasis from power and authority, towards humility (meeting challenges by working together) and courage (bringing people together to achieve that). This act of weaving demands specific intention, bringing together threads of collective effort for more robust conversations, learning and actions for the benefit of our shared planetary home and a thriving future that realises the potential arising when we focus on being better together.

### 12.5. Putting It Back Together to Move Forward

**George A. Kaplan** (Professor Emerita, University of Michigan, Ann Arbor, MI, USA; The Institute for Integrative Health, Baltimore, MD 21231, USA).

Understanding what determines the health of individuals and populations is no easy task because it is a complex, sticky problem. The “slicing and dicing” approach with various actors competing about what matters the most does not really work for complex problems, which more often than not, involve multiple determinants ranging from the cellular to the societal level, at different scales of space and time, all interacting with each other. A good example is the COVID-19 pandemic, where many dozens of factors are associated with the development of the virus, susceptibility to infection, and its consequences. We see the same complex pattern for the determinants of obesity, inequalities in health, diabetes, maternal morbidity and mortality, healthy child development—indeed, wherever we look. We need a new approach that looks at the totality of causes of population health and health inequalities in an integrative way, not one that simply dissects and supports silos of knowledge and practice. Looking backwards at Galileo’s Observatory (ca. 1609) and his attempt to look at the celestial bodies could provide a way forward towards a new kind of Observatory—a Responsive Integrative Health Observatory focused on the earth and its inhabitants. Imagine an organized and integrated approach to data from schools, health agencies, social media, environmental monitors, city planning, public agencies, police, medical care, pharmacies, the arts, etc., all being viewed in a coordinated way by such an Observatory, and providing inputs to citizens, NGOs, government agencies, funders, communities, researchers, and others! Such an observatory could help us put the puzzle pieces of what determines population health and health inequalities back together.

### 12.6. From “Me” to “We”—How to Promote Wellness and Fairness for People, Places and the Planet

**Isaac Prilleltensky** (Erwin and Barbara Mautner Chair in Community Wellbeing, University of Miami, Coral Gables, FL 33124, USA).

In order to promote health and wellness for people, places, and the planet, there is an urgent need to transform our culture. We live in a “Me Culture” in which the dominant assumption is that “I have the right to feel valued such that I may be happy”. This is an inadequate conception of a healthy society. To create a healthier planet for everyone, we must embrace a “We Culture”, according to which “We all have the right and responsibility to feel valued and add value, such that we may all experience wellness and fairness”. In a “We Culture” we balance rights with responsibilities, feeling valued with adding value, care for self and others, and a focus on wellness with a focus on fairness. A “We Culture” has the potential to correct many of the ills that currently assail our societies; among them, narcissism, inequality, xenophobia, depression, occupational disengagement and ecological entitlement. There is evidence that cultures oriented towards fairness achieve higher levels of life expectancy, literacy, knowledge of math, trust and social mobility. At the same time, these “We” cultures experience lower levels of infant mortality, homicides, imprisonment, teenage births, obesity, and mental illness. In order to move society forward, we must embrace a “We Culture” and promote its tenets in our relationships, at work, in the community, in politics, and in our relationship to non-human animals and the planet. Furthermore, we must abandon paradigms of health that are reactive, based on deficits, and that blame individuals for their misfortune. Instead, we must advance approaches that build on people’s strengths, prevention, empowerment, and community change.

### 12.7. Towards More Mindful Societies—Political Discourse That Tackles Societal Challenges at the Level of the Human Heart and Mind

**Jamie Bristow** (The Mindfulness Initiative, Secretariat to the Mindfulness All-Party Parliamentary Group, Sheffield S1 2JP, UK).

This discussion explored how mindfulness might help us meet the existential crises of the 21st Century. It seems that humanity is currently unable to handle the consequences of its own progress. From the transgression of planetary boundaries to the collapse of shared values and public trust, it is becoming that greater sophistication and maturity are required in order for us to collectively handle the increased complexity of the world we have created.

In this context, mindfulness training is emerging as one of the most promising approaches for building important inner capacities and shifting mindsets. In addition to supporting elements associated with climate action, such as increased nature connection and pro-environmental attitudes, mindfulness works more broadly to help us reclaim and reorient attention towards what matters, reflect more wisely, and act from a place of collective purpose.

In this session, we shared the work of the Mindfulness Initiative in politics and public policy and explored a three-fold model for understanding how mindfulness supports human agency, our ability to act intentionally, as proposed in our recent discussion paper Mindfulness: Developing Agency in Urgent Times (details below). 

Suggested Reading:

Bristow J. Bell R, Nixon D, Mindfulness: Developing Agency in Urgent Times. 2020, The Mindfullness Initiative, Sheffield, United Kingdom.

Bristow J. Mindfulness in politics and public policy. Curr Opin Psychol. 2019 Aug;28:87–91. doi: 10.1016/j.copsyc.2018.11.003. Epub 2018 Nov 22. PMID: 30529976.

### 12.8. Are We Being Good Ancestors—For Our Grand Our Granddaughters’ Granddaughters?

**Brian Berman ^1,2^** (^1^ The Institute for Integrative Health, 1407 Fleet Street, Baltimore, MD 21231, USA; ^2^ Department of Family and Community Medicine, Center for Integrative Medicine, University of Maryland School of Medicine, Baltimore, MD 21201, USA).

The Seventh Generation Principle of the Native American Iroquois people asks—are the decisions we are making today resulting in a sustainable world seven generations into the future? In the midst of the extraordinary challenges of our current times—a global coronavirus pandemic layered on top of a worldwide tsunami of life-threatening chronic diseases, we have to reflect on this and ask: “Are we being good ancestors for our granddaughters’ granddaughters”?

Across the globe, if health was a core value, we would all recognize there is no health without clean air, clean water, access to nutrition-rich foods, and the ability to live in safe neighbourhoods, with adequate housing and access to quality education. The policies of every department of our governments and every practice of the corporate world, from farming and agriculture to the food industry, from transportation to urban planners would be built on this value. Furthermore, within the healthcare sector, models of care would shift from a predominant focus on treatment of disease to greater emphasis on prevention and health promotion. We would remove profit as the driving force for delivery models, and in the US, we would join other developed countries in providing healthcare for all.

COVID-19, and its disproportionate impact on the poor and on communities of colour; severe environmental disasters, including wildfires and devastating floods; and social unrest due to deep underlying inequities are exposing the profound cracks in our society.

We are at an important decision point. As the Chinese character for crisis Wei Ji implies—the top part of the character meaning fear and chaos, and the bottom half, opportunity—we can let the current crisis paralyze us with fear or we can use our innate sense of infinite possibility and our incredible capacity for creativity, to actually imagine and then manifest the kind of world we DO want for our grandchildren’s grandchildren.

We have a choice. We can choose to ignore science, let our leaders feed into our fear, and allow greed to be the captains of our political and economic ships, or, we can throw out the old rule book, listen to our inner voice, our higher selves, and race to beat the clock of destruction by imagining a new reality based on our values of love, compassion, empathy…and our belief in justice and equity.

### 12.9. Depolarizing the Cultural Divide: A Path for Social Healing

**Kirk Schneider** (President of the Existential–Humanistic Institute San Francisco, CA 94102, USA; Adjunct Faculty, Columbia University, New York, NY 10027; and Saybrook University and Teachers College, Pasadena, CA 91103 USA).

America and the world need healing dialogues—especially now. In the wake of the coronavirus and George Floyd killing, many of the issues dividing the US as a nation—race, politics, class, gender, climate change, globalism, and religion—have only been magnified, and although the US Surgeon general has called for an end to bickering and partisanship, it is unclear to what extent this will take effect. What is clear, however, is that safe, mindfully structured dialogues are imperative if we are to salvage our republic and the democratic principles on which it is built. 

In this talk, I discuss my work to promote safe, mindfully structured dialogues in homes, offices, classrooms, and community centres. It is an attempt to “give away” the time-tested skills with which I have a range of intimate experience, to both laypersons and professionals; people who yearn to socially heal. The talk begins with personal observations about our polarized state, both within the United States (and by implication) the world. It follows up with a reflection on how the sense of awe toward life—issuing in part from America’s founding spirit—can serve as a counter to this polarized state. It concludes with practical strategies centred on what I call the “Experiential Democracy Dialogue”. These strategies translate awe-based sensibilities, including humility and wonder toward life, to a rediscovery of one another, a rediscovery of our potential to shape and revitalize our times. The talk finally, draws from my recent book *The Depolarizing of America: A Guidebook for Social Healing*, as well as other writings elaborating “awe-based consciousness”.

### 12.10. Taking It Forward—Project Earthrise

**Susan Prescott** (School of Medicine, University of Western Australia, the ORIGINS Projects at Telethon Kids Institute and Perth Children’s Hospital, Perth, WA, Australia; Department of Family and Community Medicine, Center for Integrative Medicine, University of Maryland School of Medicine, Baltimore; and The Institute for Integrative Health, Baltimore, MD 21231, USA)

The broad-ranging *Project Earthrise* agenda underscores the importance of ecological approaches to health on all scales—spanning from the foundational microbial ecosystems that underpin health of all ecosystems to the social, economic, and cultural value systems that drive the many risk factors for human disease and environmental destruction. It emphasises the need for integrated multilateral, multi-solving strategies that seek to understand and improve the complex relationships between human health and planetary health. This includes the socio-eco-biological interactions in our living environment (including urbanization, food systems, education, social inequity, climate change, biodiversity loss, and microbial ecology) that impact physical, mental and spiritual wellbeing, together with the wider community and societal factors that govern these. This creates many new opportunities for both personal and community actions towards grassroots positive change. It builds on the value of connectivity and efforts to promote and restore the many physical, psychological, social and environmental factors that promote resilience in every sense—recognising that even small changes signal important long-term shifts in health behaviour and in wider values. Our ongoing agenda, will promote change narratives through diverse activities that encourage greater awareness, inspire creativity, cultivate connectivity and clarify path and purpose—aimed at imagining what kind of world we want to live in, and how to make it a reality.

## 13. Conclusions

*Project Earthrise* is about sharing far more than our data, it is about sharing our passion, our spirit, and our desire for change. In the opening session, Trevor Hancock reminds us that “*we are all made of star stuff*”—an ultimate reminder of our connectivity to all things. It is a perspective that inspires our awe, our wonder, our love, our hope—respect for each other and all of life. These are the recurring themes through every session of our conference—as we are reminded of the importance of recognizing connections between the material realms we are familiar with, and the great mysteries of the spiritual realms, which are no less real because we do not understand them—realizing that these are not in fact separate. These sacred wisdoms have been preserved in traditional cultures, but they are now beginning to resurface in all societies—perhaps another measure of our connectivity and shared collective consciousness.

We must courageously go to the deep roots of where change needs to begin—not just the foundational ecosystems but the foundational value systems which govern all that we are and all that we do—our attitudes, our choices and our actions. These are the deeper conversations that are often neglected or deliberately avoided in many agendas. We must recognize that we can only solve the many interdependent challenges of our world today if we address the spiritual decay (the moral “decrepitude”, to quote Cornel West), from the deep roots, because everything flows from there.

We seek to normalize mutualistic approaches, and to place a higher value on creativity, imagination, and self-development in solving challenges at all scales. We aim to promote connectivity, where the wide-ranging work of all individuals is relevant, connected and contributing to the wider context—encouraging and inspiring purpose and meaningful progress.

Through the ongoing activities, discussions, and events of *Project Earthrise*, we hope to provide an ongoing forum to develop our strengths and our assets, to engage with others to ask meaningful questions for purposeful actions, and to create a positive, healthy, and inspiring community that creates opportunities for all who wish to be part of it. At the heart of all of these activities, we will continue to take every opportunity to return to the fundamental question of “*What kind of world do we want to live in?*” as we consider what we need to make this a reality.
